# Supplementary material for: A model for correlation-based choreographic programming
Source: PeerJ Comput Sci. 2024 Dec 24;10:e1907. doi: 10.7717/peerj-cs.1907 (PMC11784539; doi:10.7717/peerj-cs.1907)
Supplement: Supplemental Information 1 [file peerj-cs-10-1907-s001.pdf]

A Model for Correlation-based Choreographic Programming: Supplementary Material

Saverio Giallorenzo[1,2], Fabrizio Montesi[3], and Maurizio Gabbrielli[1]

[1]**Università di Bologna, Italy**
[2]**INRIA, France**
[3]**University of Southern Denmark, Denmark**

ABSTRACT

This document contains the supplementary material of the article "A Model for Correlation-based Choreographic Programming" including a full treatment of the typing system and related proofs and the proofs of the theorems on projection and compilation found in the article.

1 TYPING

In this section, we define our typing discipline for the Frontend Choreographies. Our typing checks the behaviour of sessions against protocols, given as MST (Hüttel et al., 2016). Interestingly, we retain the same syntax of traditional MST, yet we ensure that correct initial deployments do not corrupt at runtime due to inconsistencies among states and message queues.

In Section 1.1, we present the types that abstract choreographies, called global types. We define the syntax of global types and we introduce local types. The latter are abstract descriptions of the behaviour of single processes, used for type checking. We also formalise how, from a global type, we obtain a set of related local types by means of a projection procedure. In Section 1.2, we formalise the environment and the rules of our type discipline. In Section 1.3, we consider the typing of running choreographies. We illustrate why and how a choreography and its companion deployment can become inconsistent and we present a runtime typing extension to avoid inconsistencies. Finally, in Section 1.4, we present two comprehensive examples to clarify the relationship between types and running choreographies, and in Section 1.5, we formalise the properties guaranteed by our typing system.

1.1 Types and Type Projection

Global and Local types As in standard MST, we use *global types* to represent protocols from a global viewpoint and *local types* to describe the behaviour of each participant. Our type system checks that a set of local types, each abstracting the behaviour of a process in a choreography, coherently follows a global type. We report in Figure 1 the syntax of global types G and local types T.

A global type $\mathtt{A} \longrightarrow\!\!\triangleright\, \mathtt{B}.\{o_i(U_i);G_i\}_i$ abstracts a communication, where $\mathtt{A}$ can send to $\mathtt{B}$ a message on any of the operations o_i and continue with the respective continuation G_i. A carried type U types the value exchanged in the message. In local types, $!\mathtt{A}.\{o_i(U_i);T_i\}_i$ abstracts the sending of a message of type U_i to role $\mathtt{A}$ on one of the operations o_i, with continuation T_i. Dually, $?\mathtt{A}.\{o_i(U_i);T_i\}_i$ abstracts the offering of an input choice among the operations o_i, with continuation T_i. The other terms for recursion and end of types are standard. As done for FC, also in types we omit curly brackets when outputs and inputs comprise only one operation and also omit end.

As an example, we report in Figure 2 two global types, G_1 and G_2, that abstract the choreographies. In particular, G_1 types session k, created at locations $(l_{\mathtt{S}}, l_{\mathtt{B}})$ and G_2 types session k', created at location $(l_{\mathtt{D}})$. We also write operations followed by empty parentheses when the type of their message U is $\mathbf{unit}$.

Type Projection. To relate global types to the behaviour of processes in choreographies, we project a global type G into a set of local types, each corresponding to the behaviour of a single role. This is a consolidated technique of MST (Honda et al., 2016). Given a global type G that describes the expected distributed behaviour of the participants, we define a procedure (called "projection") that generates types

$$\begin{array}{llll}
\textit{Global Types} & G & ::= & \mathtt{A} \longrightarrow \mathtt{B}.\{o_i(U_i);G_i\}_i \quad (\textit{communication}) \\
& & \mid & \mathsf{rec}\ \mathbf{t}.G \ \mid\ \mathbf{t} \quad (\textit{recursion}) \\
& & \mid & \mathsf{end} \quad (\textit{end})
\end{array}$$

$$\begin{array}{llll}
\textit{Local Types} & T & ::= & \oplus\mathtt{A}.\{o_i(U_i);T_i\}_i \quad (\textit{send}) \\
& & \mid & \&\mathtt{A}.\{o_i(U_i);T_i\}_i \quad (\textit{receive}) \\
& & \mid & \mathsf{rec}\ \mathbf{t}.T \ \mid\ \mathbf{t} \quad (\textit{recursion}) \\
& & \mid & \mathsf{end} \quad (\textit{end})
\end{array}$$

$$\textit{Sort Types} \quad U \ ::= \ \mathbf{unit} \ \mid\ \mathbf{int} \ \mid\ \mathbf{bool} \ \mid\ \mathbf{str} \ \mid\ \ldots$$

Figure 1. Frontend Choreographies, syntax of Global and Local Types.

$$\begin{array}{ll}
G_1 = & \mathtt{C} \longrightarrow \mathtt{S}.\mathit{buy}(\mathbf{str}); \\
& \mathtt{S} \longrightarrow \mathtt{B}.\mathit{reqPay}(\mathbf{int}); \\
& \mathtt{C} \longrightarrow \mathtt{B}.\mathit{accPay}(\mathbf{str}); \\
& \mathtt{B} \longrightarrow \mathtt{C}.\{ \\
& \quad \mathit{ok}();\mathtt{B} \longrightarrow \mathtt{S}.\mathit{ok}(), \\
& \quad \mathit{ko}();\mathtt{B} \longrightarrow \mathtt{S}.\mathit{ko}() \\
& \}
\end{array}
\qquad
\begin{array}{ll}
G_2 = & \mathtt{S} \longrightarrow \mathtt{D}.\mathit{quoteShipping}(\mathbf{str}); \\
& \mathtt{D} \longrightarrow \mathtt{S}.\mathit{shippingCosts}(\mathbf{int}); \\
& \mathtt{S} \longrightarrow \mathtt{D}.\{ \\
& \quad \mathit{sendShipping}(), \\
& \quad \mathit{abortShipping}() \\
& \}
\end{array}$$

Figure 2. Global types G_1 (left) and G_2 (right) abstract the respective choreographies presented in Figures 4 and 5 of the article.

that programmers can use to check the conformance of the implementation of each participant. In our settings, we introduce the projection procedure from global to local types because we can have parallel compositions of choreographies (e.g., $C_1 \mid C_2$) with partial actions. Since we need to be able to type partial actions, we follow the standard practice of MST and use global-to-local-type projection to handle both complete (e.g., (*com*)) and partial (e.g., (*send*)) actions.

We report in Figure 3 the projection of global types, defined following Montesi and Yoshida (2013). $[\![G]\!]_{\mathtt{A}}$ denotes the projection of G onto the role $\mathtt{A}$. Intuitively, $[\![G]\!]_{\mathtt{A}}$ gives an encoding of the local actions expected by role $\mathtt{A}$ in the global type G. When projecting a communication, we require the local behaviour of all roles not involved in it to be merged with the merging operator $\sqcup$. Like in (Montesi and Yoshida, 2013), $T \sqcup T'$ is isomorphic to T and T' up to branching, where all branches of T or T' with distinct operations are also included, formally

$$T \sqcup T' = \begin{cases} T & \text{if } T = T' \\ \&\mathtt{A}.\left\{ \begin{array}{l} \{\, o_h(U_h);T_h \,\}_{h \in I \setminus J} \ \cup \\ \{\, o_h(U_h);T'_h \,\}_{h \in J \setminus I} \ \cup \\ \{\, o_h(U_h);T_h \sqcup T'_h \,\}_{h \in J \cap I} \end{array} \right\} & \begin{array}{l} \text{if } T = \&\mathtt{A}.\{o_i(U_i);T_i\}_{i \in I} \\ \text{and } T' = \&\mathtt{A}.\{o_j(U_j);T'_j\}_{j \in J} \end{array} \end{cases}$$

1.2 Type Checking

Now that we defined the relation between global and local types, we can proceed to present our system that guarantees that sessions in choreographies follow their types.

1.2.1 Environments

We define our typing environments $\Gamma, \Gamma', \ldots$ as reported in Figure 4.

The typing of *variables* denotes that a process $\mathtt{p}$ has in its state a variable x of type U. We assume that we can write $\Gamma, \mathtt{p}.x \colon U$ only if either x has not been typed yet in Γ or it is already associated with the

$$
\begin{aligned}
[\![\mathtt{B} \multimap \mathtt{C}.\{o_i(U_i);G_i\}_i]\!]_{\mathtt{A}} &= \begin{cases} \oplus\mathtt{C}.\{o_i(U_i);[\![G_i]\!]_{\mathtt{C}}\}_i & \text{if } \mathtt{A}=\mathtt{B} \\ \&\mathtt{B}.\{o_i(U_i);[\![G_i]\!]_{\mathtt{C}}\}_i & \text{if } \mathtt{A}=\mathtt{C} \\ \bigsqcup_i [\![G_i]\!]_{\mathtt{A}} & \text{otherwise} \end{cases} \\
[\![\mathsf{rec}\ \mathbf{t}.G]\!]_{\mathtt{A}} &= \begin{cases} \mathsf{rec}\ \mathbf{t}.\,[\![G]\!]_{\mathtt{A}} & \text{if } \mathtt{A}\in G \\ \mathsf{end} & \text{otherwise} \end{cases} \\
[\![\mathbf{t}]\!]_{\mathtt{A}} &= \mathbf{t} \\
[\![\mathsf{end}]\!]_{\mathtt{A}} &= \mathsf{end}
\end{aligned}
$$

Figure 3. Frontend Choreographies, Global Types Projection.

$$
\begin{array}{rlll}
\Gamma ::= & \varnothing & & (\textit{empty environment}) \\
| & \Gamma, \mathtt{p}.x : U & & (\textit{variable}) \\
| & \Gamma, X : \Gamma & & (\textit{definition}) \\
| & \Gamma, k[\mathtt{A}] : T & & (\textit{local session}) \\
| & \Gamma, \mathtt{p} : k[\mathtt{A}] & & (\textit{ownership}) \\
| & \Gamma, \tilde{l} : G\langle \mathtt{A}|\tilde{\mathtt{B}}|\tilde{\mathtt{C}}\rangle & & (\textit{service})
\end{array}
$$

Figure 4. Frontend Choreographies, Typing Environment.

same type U (formally let $\{u,u'\} \in U$, if $u = u'$ then $\Gamma, \mathtt{p}.x\colon u, \mathtt{p}.x\colon u' = \Gamma, \mathtt{p}.x\colon u$). We assume a similar convention for all the identifiers in Γ except for *service* typings, whose rule for set inclusion is detailed at the end of this section. The typing of *definition* of recursive procedures associates a procedure identifier X to a typing environment Γ. A *local session* typing $k[\mathtt{A}]\colon T$ states that role $\mathtt{A}$ in session k follows the local type T. An *ownership* typing $\mathtt{p}\colon k[\mathtt{A}]$ states that process $\mathtt{p}$ owns the role $\mathtt{A}$ in session k. Hence, each process can participate in multiple sessions, but can play only one role in each session and we intend $\Gamma, \mathtt{p}\colon k[A]$ valid as long as $\mathtt{p}$ is not associated with other roles in the same session k in Γ. A service typing $\tilde{l} : G\langle \mathtt{A}|\tilde{\mathtt{B}}|\tilde{\mathtt{C}}\rangle$ types with a global type G all sessions created by contacting the services at the locations $\tilde{l}$. In the typing,

- $\mathtt{A}$ is the role that the active process (the starter) should play;
- $\tilde{\mathtt{B}}$ are the roles respectively played by each service process at l in $\tilde{l}$—we assume that each l plays a unique role, so the lengths of $\tilde{\mathtt{B}}$ and $\tilde{l}$ are the same;
- $\tilde{\mathtt{C}}$ are the roles implemented by the choreography that we are typing—we assume $\tilde{\mathtt{C}} \subseteq \tilde{\mathtt{B}}$, i.e., that $\tilde{\mathtt{C}}$ contain a subset of the roles in $\tilde{\mathtt{B}}$, ordered following the order in $\tilde{\mathtt{B}}$ (as of Definition 6).

Regarding set inclusion of service typings, when we write $\Gamma = \Gamma', \tilde{l}\colon G\langle \mathtt{A}|\tilde{\mathtt{B}}|\tilde{\mathtt{C}}\rangle$ we assume that:

- $\{\mathtt{A},\tilde{\mathtt{B}}\} = \mathbf{roles}(G)$, where function **roles** returns the set of roles in G;
- the locations $\tilde{l}$ are ordered lexicographically;
- the locations in $\tilde{l}$ do not appear in any other service typing in Γ;
- that either:
  - $\tilde{l}$ does not appear in Γ' and the resulting Γ includes it, formally $\tilde{l} \notin \mathbf{dom}(\Gamma')$ and $\Gamma = \Gamma',\ \tilde{l}\colon G\langle \mathtt{A}|\tilde{\mathtt{B}}|\tilde{\mathtt{C}}\rangle$; or
  - $\tilde{l}$ appears in Γ', such that $\Gamma' = \Gamma'', \tilde{l}\colon G\langle \mathtt{A}|\tilde{\mathtt{B}}|\tilde{\mathtt{D}}\rangle$, and $\{\tilde{\mathtt{C}}\} \cap \{\tilde{\mathtt{D}}\} = \varnothing$, i.e., the roles in $\tilde{\mathtt{C}}$ do not appear in $\tilde{\mathtt{D}}$. The resulting Γ includes in the service typing of $\tilde{l}$ the merged list of roles in $\tilde{\mathtt{C}}$ and $\tilde{\mathtt{D}}$, following the lexicographic order in $\tilde{\mathtt{B}}$. We write the merge as $\tilde{\mathtt{D}} \bowtie_{\tilde{\mathtt{B}}} \tilde{\mathtt{C}}$ (see Definition 7) and $\Gamma = \Gamma'', \tilde{l}\colon G\langle \mathtt{A}|\tilde{\mathtt{B}}|\tilde{\mathtt{D}} \bowtie_{\tilde{\mathtt{B}}} \tilde{\mathtt{C}}\rangle$.

$$\frac{\Gamma,\tilde{l} : G\langle \mathtt{A}|\tilde{\mathtt{B}}|\tilde{\mathtt{B}}\rangle, \mathbf{init}(\ (\mathsf{p}[\mathtt{A}],\widetilde{\mathsf{q}[\mathtt{B}]}),k,G\ ) \vdash C \quad \tilde{\mathsf{q}} \notin \Gamma}{\Gamma,\tilde{l} : G\langle \mathtt{A}|\tilde{\mathtt{B}}|\tilde{\mathtt{B}}\rangle \vdash \mathsf{start}\ k : \mathsf{p}[\mathtt{A}] \leftrightarrow \widetilde{l.\mathsf{q}[\mathtt{B}]};C} \ \lfloor^{\mathsf{T}}|_{\mathsf{Start}}\rceil$$

$$\frac{\Gamma, \mathbf{init}(\ \mathsf{p}[\mathtt{A}],k,G\ ) \vdash C \quad \tilde{l} : G\langle \mathtt{A}|\tilde{\mathtt{B}}|\varnothing\rangle \in \Gamma}{\Gamma \vdash \mathsf{req}\ k : \mathsf{p}[\mathtt{A}] \leftrightarrow \widetilde{l.\mathtt{B}};C} \ \lfloor^{\mathsf{T}}|_{\mathsf{Req}}\rceil$$

$$\frac{\Gamma,\tilde{l}' : G\langle \mathtt{A}|\tilde{\mathtt{B}}|\varnothing\rangle, \mathbf{init}(\ \widetilde{\mathsf{q}[\mathtt{C}]},k,G\ ) \vdash C \quad \tilde{l} \subseteq \tilde{l}' \quad \tilde{\mathsf{q}} \notin \Gamma}{\Gamma,\tilde{l}' : G\langle \mathtt{A}|\tilde{\mathtt{B}}|\tilde{\mathtt{C}}\rangle \vdash \mathsf{acc}\ k : \widetilde{l.\mathsf{q}[\mathtt{C}]};C} \ \lfloor^{\mathsf{T}}|_{\mathsf{Acc}}\rceil$$

$$\frac{\Gamma \vdash \mathsf{p} : k[\mathtt{A}], \mathsf{q} : k[\mathtt{B}] \quad j \in I \quad \Gamma \vdash \mathsf{p}.e : U_j \quad \Gamma, \mathsf{q}.x : U_j, k[\mathtt{A}] : T_j, k[\mathtt{B}] : T'_j \vdash C}{\Gamma, k[\mathtt{A}] : \oplus\,\mathtt{B}.\{o_i(U_i);T_i\}_{i\in I}, k[\mathtt{B}] : \&\mathtt{A}.\{o_i(U_i);T'_i\}_{i\in I} \vdash k : \mathsf{p}[\mathtt{A}].e \rightarrow \mathsf{q}[\mathtt{B}].o_j(x);C} \ \lfloor^{\mathsf{T}}|_{\mathsf{Com}}\rceil$$

$$\frac{j \in I \quad \Gamma \vdash \mathsf{p} : k[\mathtt{A}] \quad \Gamma \vdash \mathsf{p}.e : U_j \quad \Gamma, k[\mathtt{A}] : T_j \vdash C}{\Gamma, k[\mathtt{A}] : \oplus\,\mathtt{B}.\{o_i(U_i);T_i\}_{i\in I} \vdash k : \mathsf{p}[\mathtt{A}].e \rightarrow \mathtt{B}.o_j;C} \ \lfloor^{\mathsf{T}}|_{\mathsf{Send}}\rceil$$

$$\frac{\Gamma \vdash \mathsf{q} : k[\mathtt{B}] \quad \forall j \in I.\ \Gamma, \mathsf{q}.x_j : U_j, k[\mathtt{B}] : T_j \vdash C_j}{\Gamma, k[\mathtt{B}] : \&\mathtt{A}.\{o_i(U_i);T_i\}_{i\in I} \vdash k : \mathtt{A} \rightarrow \mathsf{q}[\mathtt{B}].\{o_j(x_j);C_j\}_{j\in I\cup J}} \ \lfloor^{\mathsf{T}}|_{\mathsf{Recv}}\rceil$$

$$\frac{\Gamma \vdash \mathsf{p}.e : \mathbf{bool} \quad \Gamma \vdash C_1 \quad \Gamma \vdash C_2}{\Gamma \vdash \mathtt{if}\ \mathsf{p}.e\ \{C_1\}\ \mathtt{else}\ \{C_2\}} \ \lfloor^{\mathsf{T}}|_{\mathsf{Cond}}\rceil$$

$$\frac{\Gamma, X : \Gamma' \vdash C \quad \Gamma', X : \Gamma' \vdash C' \quad \Gamma'|_{\mathbf{locs}} \subseteq \Gamma}{\Gamma \vdash \mathsf{def}\ X = C'\ \mathsf{in}\ C} \ \lfloor^{\mathsf{T}}|_{\mathsf{Def}}\rceil$$

$$\frac{\Gamma_1 \vdash C_1 \quad \Gamma_2 \vdash C_2}{\Gamma_1, \Gamma_2 \vdash C_1 \mid C_2} \ \lfloor^{\mathsf{T}}|_{\mathsf{Par}}\rceil$$

$$\frac{\mathsf{end}(\Gamma)}{\Gamma \vdash \mathbf{0}} \ \lfloor^{\mathsf{T}}|_{\mathsf{End}}\rceil \qquad \frac{\Gamma'' \subseteq \Gamma' \quad \mathsf{end}(\Gamma)}{\Gamma, \Gamma', X : \Gamma'' \vdash X} \ \lfloor^{\mathsf{T}}|_{\mathsf{Call}}\rceil$$

Figure 5. Frontend Choreographies, Typing Rules.

We underline that the annotation $\tilde{\mathtt{C}}$ in service typings plays two important parts: it enables the composition of choreographies and it ensures that only one choreography implements a specific role. This is mirrored in the composition $\Gamma = \Gamma', \tilde{l} : G\langle \mathtt{A}|\tilde{\mathtt{B}}|\tilde{\mathtt{C}}\rangle$ where, if Γ' already contains the typing for some roles $\tilde{\mathtt{D}}$ in $\tilde{l}$, Γ will contain the additional roles defined in $\tilde{\mathtt{C}}$ (provided $\tilde{\mathtt{D}}$ and $\tilde{\mathtt{C}}$ contain distinct roles).

1.2.2 Typing Judgements and Rules

A judgement $\Gamma \vdash C$ states that the choreography C follows the specifications given in Γ. We comment on the typing rules reported in Figure 5.

Rule $\lfloor^{\mathsf{T}}|_{\mathsf{Start}}\rceil$ types a session start. In the first premise, the service typing $\tilde{l} : G\langle \mathtt{A}|\tilde{\mathtt{B}}|\tilde{\mathtt{B}}\rangle$ checks that the continuation implements all the roles in protocol G. The function **init** assembles the typing environment that correctly types—with the appropriate ownerships and local typings—the freshly-started session k, given the global type G and the processes in $\tilde{\mathsf{p}}$, each playing its corresponding role in $\tilde{\mathtt{B}}$. Formally,

$$\mathbf{init}(\ \widetilde{\mathsf{p}[\mathtt{A}]}, k, G\ ) = \left\{\ \mathsf{q} : k[\mathtt{B}],\ k[\mathtt{B}] : [\![G]\!]_{\mathtt{B}} \mid \mathsf{q}[\mathtt{B}] \in \left\{\widetilde{\mathsf{p}[\mathtt{A}]}\right\}\ \right\}.$$

where the type of each process $\mathsf{p} \in \tilde{\mathsf{p}}$ playing role $\mathtt{B} \in \tilde{\mathtt{B}}$ is the local type projection $[\![G]\!]_{\mathtt{B}}$ of the global type G. In $\lfloor^{\mathsf{T}}|_{\mathsf{Start}}\rceil$, we abuse the notation $\tilde{\mathsf{q}} \notin \Gamma$ to check that all freshly created processes in $\tilde{\mathsf{q}}$ do not appear in Γ (i.e., there is no variable or ownership typings in Γ associated with any process in $\tilde{\mathsf{q}}$).

Rule $\lfloor^{\mathsf{T}}|_{\mathsf{Req}}\rceil$ types (*req*) terms and is similar to $\lfloor^{\mathsf{T}}|_{\mathsf{Start}}\rceil$, although it only performs the checks for the process p, playing role $\mathtt{A}$, that requests the creation of the new session k. Dually, $\lfloor^{\mathsf{T}}|_{\mathsf{Acc}}\rceil$ mirrors Rule

$\lfloor^{\mathsf{T}}|_{\mathsf{Start}}\rceil$ and $\tilde{l} \subseteq \tilde{l}'$ checks that (following Definition 6) the list of locations of the service typing in Γ includes the locations in the (acc) term. In the premise, we type the continuation C with $\tilde{l}' : G\langle \mathtt{A}|\tilde{\mathtt{B}}|\varnothing\rangle$, which forces (acc) (and $(start)$) terms on a given set of locations to only appear once in well-typed choreographies.

Rule $\lfloor^{\mathsf{T}}|_{\mathsf{Com}}\rceil$ types a complete communication. From left to right the premises check that:

1. the sender p and the receiver q own their respective roles in the session;
2. since $j \in I$:
    - operation o_j can be effectively selected by the sender, according to its local type;
    - similarly, o_j is among the operations offered by the receiver, according to its local type;
3. the expression of the sender (e) has the type—the judgement $\Gamma \vdash e : U$ reads as "expression e has type U"—U_j, expected by the protocol;
4. the resulting environment $\Gamma, \mathsf{q}.x : U_j, k[\mathtt{A}] : T_j, k[\mathtt{B}] : T'_j$ correctly types the continuation C, in particular that:
    - the receiver q correctly uses the reception variable x in C;
    - processes p and q proceed according to their local types, respectively T_j and T'_j.

Rules $\lfloor^{\mathsf{T}}|_{\mathsf{Send}}\rceil$ and $\lfloor^{\mathsf{T}}|_{\mathsf{Recv}}\rceil$ share part of the checks commented for $\lfloor^{\mathsf{T}}|_{\mathsf{Com}}\rceil$ and judge the respective partial terms $(send)$ and $(recv)$. Note that, as in standard MST, the local typing of the branching process q is contravariant wrt the branches in the choreography, i.e., the rule $\lfloor^{\mathsf{T}}|_{\mathsf{Recv}}\rceil$ checks that the operations supported by the typing $o_i, i \in I$ are at least a subset of the actual operations $o_j, j \in I \cup J$ provided in the $(recv)$ term.

Rule $\lfloor^{\mathsf{T}}|_{\mathsf{Cond}}\rceil$ checks that the expression of a conditional has a compatible type (**bool**) and that both branches C_1 and C_2 are correctly typed by Γ.

Rule $\lfloor^{\mathsf{T}}|_{\mathsf{Def}}\rceil$ checks procedure definitions. Here, function $|_{\mathbf{locs}}$ applied to an environment Γ returns all service typings in it. In the rule, we write $\Gamma'|_{\mathbf{locs}} \subseteq \Gamma$ to check that the body of the recursive procedure does not introduce unexpected services, i.e., services that are not present at the top level.

In rule $\lfloor^{\mathsf{T}}|_{\mathsf{Par}}\rceil$ we extend the set inclusion for Γ_1, Γ_2 point-wise to the identifiers in Γ to merge typings and to check that choreographies executing in parallel do not implement overlapping roles at locations, and that only one choreography defines the behaviour of a process.

In rule $\lfloor^{\mathsf{T}}|_{\mathsf{End}}\rceil$, the predicate $\mathsf{end}(\Gamma)$ holds if the protocols for all sessions in Γ have terminated (i.e., all local typings have type end).

$\lfloor^{\mathsf{T}}|_{\mathsf{Call}}\rceil$ checks a procedure call. The premise $\Gamma'' \subseteq \Gamma'$ checks that procedure X does not introduce unexpected typings (and, by extension, behaviour) wrt the active sessions contained in Γ'. The premise $\mathsf{end}(\Gamma)$ makes sure that the remaining sessions in the typing environment have all terminated.

1.3 Runtime Typing

To prove that well-typed FC programs never go wrong, we need to pay attention to how their deployments evolve at runtime. For example, in rule $\lfloor^{\mathsf{C}}|_{\mathsf{Send}}\rceil$, the deployment D must contain the proper queue where the sender can deliver its message: a remarkable difference wrt previous work on choreographies, where such conditions do not exist and choreographies can always continue execution (see, e.g., (Qiu et al., 2007; Busi et al., 2006; Carbone et al., 2012; Carbone and Montesi, 2013)).

To guarantee that well-typed FC programs never go wrong, we must guarantee that their companion deployments evolve consistently. We address this issue by extending our typing discipline to check runtime states.

Wrong Deployments. We want to rule out "wrong" deployments. Intuitively, we say that a deployment is wrong wrt a choreography if e.g., processes have undefined variables that are used in the choreography or a message queue does not contain messages as expected by the protocol of the session in which it is used.

Wrong deployments may cause unpredictable executions or faulty behaviour, such as deadlocks. We illustrate the consequences of having wrong deployments with this simple running choreography:

$D, k\colon \mathtt{p}[\mathtt{A}].y \longrightarrow \mathtt{q}[\mathtt{B}].o(x); \mathbf{0}$

- *(uninitialised variables)* assume that D is such that the state of process p in D, $D(\mathtt{p})$, does not contain a value for variable y; then the condition $\mathbf{eval}(\ y, D(\mathtt{p})\ )$ given in rule $\lfloor^{\mathsf{D}}|_{\mathsf{Send}}\rceil$ is undefined and rule $\lfloor^{\mathsf{C}}|_{\mathsf{Com}}\rceil$ cannot be applied, causing the choreography to get stuck.
- *(protocol violations)* assume that $D(\ k[\mathtt{A}\rangle\mathtt{B}]\ ) = (o', v)$ where $o \neq o'$. Namely, that *i*) in session k process q (playing role B) has a message in its receiving queue from process p (playing role A) and *ii*) the operation of the message is o', different from operation o expected in the choreography. If we let the choreography reduce following the previous point, it ends up deadlocked. After the reduction, the queue used by p contains in its head the message (o', v) and we cannot apply rule $\lfloor^{\mathsf{C}}|_{\mathsf{Recv}}\rceil$, as it expects to find a message for o at that position.

To avoid these outcomes, we extend our type system to prove that, given a well-typed choreography and a non-wrong companion deployment, our semantics never produces wrong deployments. Note that this development is transparent to programmers, since default deployments are trivially never wrong.

Runtime Global Types To capture asynchrony and partial runtime states, we extend the syntax of global types with:

$$
\begin{array}{rll}
G ::= & \ldots & \\
| & \oplus\mathtt{AB}.\{o_i(U_i)\}; G & (\textit{global choice}) \\
| & \&\mathtt{AB}.\{o_i(U_i); G_i\}_{i \in I} & (\textit{global branch}) \\
| & \mathtt{A}\rangle\mathtt{B}.o(U); G & (\textit{global buffer})
\end{array}
$$

Global choice and branch are the equivalent of a complete communication $\mathtt{A} \longrightarrow \mathtt{B}.o(U); G$ where: $\oplus\mathtt{AB}.\{o_i(U_i)\}; G$ means that role A can choose to send a message to role B on operation o_i with type U_i, proceeding with continuation G; while $\&\mathtt{AB}.\{o_i(U_i); G_i\}_{i \in I}$ means that B can receive a message from A on any operation $o_i, i \in I$, proceeding with the related continuation G_i.

When the choice performed by A is applied to the branch controlled by B, we obtain term $\mathtt{A}\rangle\mathtt{B}.o(U)$, which marks that A has sent the message but B still has to consume it.

Semantics of Global Types To express the (abstract) execution of protocols, we give a semantics for global types. Formally, $G \to G'$ is the smallest relation on the recursion-unfolding of global types satisfying the rules in Figure 6.

Rule $\lfloor^{\mathsf{G}}|_{\mathsf{Send}}\rceil$ allows the sending of a message from a (*global choice*). The continuation G' is obtained from the application of the sending to the corresponding (*global branch*), with function ${}^{\mathtt{A}\rangle\mathtt{B}}_{o_i}{\downarrow}G$ that transforms the related branch in G into a (*global buffer*) on the selected operation o_i, followed by the respective continuation G_i.

The actual reception of the message is executed in rule $\lfloor^{\mathsf{G}}|_{\mathsf{Recv}}\rceil$. In $\lfloor^{\mathsf{G}}|_{\mathsf{Eq}}\rceil$ we model the splitting of complete communications and recursion unfolding with the structural equivalence $\equiv_{\mathsf{G}}$, the smallest congruence defined by the rules in Figure 6. To capture the semantics of asynchronous message delivery, we define the swap relation $\simeq_{\mathsf{G}}$ as the smallest congruence defined by the rules in Figure 6. Both congruences are similar to what we presented for choreographies in Section 2.2 of the article. Note that rules $\lfloor^{\mathsf{GS}}|_{\mathsf{ChoBrc}}\rceil$ and $\lfloor^{\mathsf{GS}}|_{\mathsf{ChoBuf}}\rceil$ enable the swapping of choice terms with receptions, as long as the swap preserves the causal consistency between operations (i.e., we do not swap a send operation that is causally dependent on a receive operation on the same role).

Runtime Type Checking and Typing Rules We extend the typing rules given in the previous section to check runtime terms. The extension consists of *i*) new terms for Γ, and *ii*) the introduction of rule $\lfloor^{\mathsf{T}}|_{\mathsf{DC}}\rceil$ to type runtime choreographies. We extend the grammar of typing environments with

$$
\begin{array}{rll}
\Gamma ::= & \ldots & \\
| & \Gamma, \mathtt{p}@l & (\textit{location}) \\
| & \Gamma, k[\mathtt{A}\rangle\mathtt{B}] : T & (\textit{buffer})
\end{array}
$$

$$\frac{o \in \bigcup_i \{o_i\} \quad G' = {}^{\mathtt{A}\rangle\mathtt{B}}_{o}\!\downarrow G}{\oplus \mathtt{AB}.\{o_i(U_i)\};G \quad \to \quad G'} \lfloor^{\mathsf{G}}|_{\mathsf{Send}}\rceil \qquad \frac{}{\mathtt{A}\rangle\mathtt{B}.o(U);G \quad \to \quad G} \lfloor^{\mathsf{G}}|_{\mathsf{Recv}}\rceil$$

$$\frac{\mathcal{R} \in \{\equiv_{\mathtt{G}}, \simeq_{\mathtt{G}}\} \quad G\,\mathcal{R}\,G_1 \quad G_1 \to G_1' \quad G_1'\,\mathcal{R}\,G'}{G \quad \to \quad G'} \lfloor^{\mathsf{G}}|_{\mathsf{Eq}}\rceil$$

Reduction Rules.

$$\mathtt{A} \longrightarrow\!\!\triangleright\, \mathtt{B}.\{o_i(U_i);G_i\} \quad \equiv_{\mathtt{G}} \quad \oplus\mathtt{AB}.\{o_i(U_i)\};\&\mathtt{AB}.\{o_i(U_i);G_i\}$$

$$G[\mathsf{rec}\ \mathbf{t}.G'] \quad \equiv_{\mathtt{G}} \quad G[\,G'[\mathsf{rec}\ \mathbf{t}.G'/\mathbf{t}]\,]$$

Structural Congruence.

$$\frac{\mathtt{A} \neq \mathtt{C} \vee \mathtt{B} \neq \mathtt{D}}{\oplus\mathtt{AB}.\{o_i(U_i)\};\oplus\mathtt{CD}.\{o_j(U_j)\} \simeq_{\mathtt{G}} \oplus\mathtt{CD}.\{o_j(U_j)\};\oplus\mathtt{AB}.\{o_i(U_i)\}} \lfloor^{\mathsf{GS}}|_{\mathsf{ChoCho}}\rceil$$

$$\frac{\mathtt{A} \neq \mathtt{C} \vee \mathtt{B} \neq \mathtt{D}}{\&\mathtt{AB}.\{o_i(U_i);\&\mathtt{CD}.\{o_j(U_j);G_{ij}\}\} \simeq_{\mathtt{G}} \&\mathtt{CD}.\{o_j(U_j);\&\mathtt{AB}.\{o_i(U_i);G_{ij}\}\}} \lfloor^{\mathsf{GS}}|_{\mathsf{BrcBrc}}\rceil$$

$$\frac{\mathtt{A} \neq \mathtt{D}}{\oplus\mathtt{AB}.\{o_i(U_i)\};\&\mathtt{CD}.\{o_j(U_j);G_j\} \simeq_{\mathtt{G}} \&\mathtt{CD}.\{o_j(U_j);\oplus\mathtt{AB}.\{o_i(U_i)\};G_j\}} \lfloor^{\mathsf{GS}}|_{\mathsf{ChoBrc}}\rceil$$

$$\frac{\mathtt{A} \neq \mathtt{C} \vee \mathtt{B} \neq \mathtt{D}}{\mathtt{A}\rangle\mathtt{B}.o(U);\&\mathtt{CD}.\{o_j(U_j);G_j\} \simeq_{\mathtt{G}} \&\mathtt{CD}.\{o_j(U_j);\mathtt{A}\rangle\mathtt{B}.o(U);G_j\}} \lfloor^{\mathsf{GS}}|_{\mathsf{BufBrc}}\rceil$$

$$\frac{\mathtt{A} \neq \mathtt{C} \vee \mathtt{B} \neq \mathtt{D}}{\mathtt{A}\rangle\mathtt{B}.o(U);\mathtt{C}\rangle\mathtt{D}.o'(U') \simeq_{\mathtt{G}} \mathtt{C}\rangle\mathtt{D}.o'(U');\mathtt{A}\rangle\mathtt{B}.o(U)} \lfloor^{\mathsf{GS}}|_{\mathsf{BufBuf}}\rceil$$

$$\frac{\mathtt{A} \neq \mathtt{D}}{\oplus\mathtt{AB}.\{o_i(U_i)\};\mathtt{C}\rangle\mathtt{D}.o(U) \simeq_{\mathtt{G}} \mathtt{C}\rangle\mathtt{D}.o(U);\oplus\mathtt{AB}.\{o_i(U_i)\}} \lfloor^{\mathsf{GS}}|_{\mathsf{ChoBuf}}\rceil$$

Swap Relation.

$$\begin{array}{lcll} {}^{\mathtt{A}\rangle\mathtt{B}}_{o_j}\!\downarrow\&\mathtt{AB}.\{o_i(U_i);G_i\}_{i\in I} & = & \mathtt{A}\rangle\mathtt{B}.o_j(U_j);G_j & \text{if } j \in I \\ {}^{\mathtt{A}\rangle\mathtt{B}}_{o_j}\!\downarrow\&\mathtt{CD}.\{o_i(U_i);G_i\}_{i\in I} & = & \&\mathtt{CD}.\{o_i(U_i);{}^{\mathtt{A}\rangle\mathtt{B}}_{o_j}\!\downarrow G_i\} & \text{if } \mathtt{A} \neq \mathtt{C} \vee \mathtt{B} \neq \mathtt{D} \end{array}$$

$${}^{\mathtt{A}\rangle\mathtt{B}}_{o}\!\downarrow\mathtt{C}\rangle\mathtt{D}.o(U);G = \mathtt{C}\rangle\mathtt{D}.o(U);{}^{\mathtt{A}\rangle\mathtt{B}}_{o}\!\downarrow G \qquad {}^{\mathtt{A}\rangle\mathtt{B}}_{o}\!\downarrow \oplus\mathtt{CD}.\{o_i(U_i)\};G = \oplus\mathtt{CD}.\{o_i(U_i)\};{}^{\mathtt{A}\rangle\mathtt{B}}_{o}\!\downarrow G$$

$${}^{\mathtt{A}\rangle\mathtt{B}}_{o}\!\downarrow\mathsf{rec}\ \mathbf{t}.G = \mathsf{rec}\ \mathbf{t}.G \qquad {}^{\mathtt{A}\rangle\mathtt{B}}_{o}\!\downarrow\mathbf{t} = \mathbf{t} \qquad {}^{\mathtt{A}\rangle\mathtt{B}}_{o}\!\downarrow\mathsf{end} = \mathsf{end}$$

Application Function.

Figure 6. Global types, semantics.

$$[\![\mathtt{C} \longrightarrow \mathtt{D}.\{o_i(U_i);G_i\}]\!]^{\mathtt{A}}_{\mathtt{B}} \quad = \quad \begin{cases} \mathsf{end} & \text{if } \mathtt{C}=\mathtt{A} \ \wedge \ \mathtt{D}=\mathtt{B} \\ \bigsqcup_i [\![G_i]\!]^{\mathtt{A}}_{\mathtt{B}} & \text{otherwise} \end{cases}$$

$$[\![\mathtt{C}\rangle \mathtt{D}.o(U);G]\!]^{\mathtt{A}}_{\mathtt{B}} \quad = \quad \begin{cases} \&\mathtt{A}.o(U);[\![G]\!]^{\mathtt{A}}_{\mathtt{B}} & \text{if } \mathtt{C}=\mathtt{A} \wedge \ \mathtt{D}=\mathtt{B} \\ [\![G]\!]^{\mathtt{A}}_{\mathtt{B}} & \text{otherwise} \end{cases}$$

$$[\![\mathbf{t}]\!]^{\mathtt{A}}_{\mathtt{B}} = [\![\mathsf{end}]\!]^{\mathtt{A}}_{\mathtt{B}} = [\![\mathsf{rec}\ \mathbf{t}.G]\!]^{\mathtt{A}}_{\mathtt{B}} \quad = \quad \mathsf{end}$$

Figure 7. Frontend Choreographies, Buffer Type Projection.

where $\Gamma, \mathtt{p}@l$ states that process $\mathtt{p}$ runs at location l and a *buffer* typing $k[\mathtt{A}\rangle\mathtt{B}] : T$ types the messages in the queue where the process implementing role $\mathtt{B}$ in session k receives messages from role $\mathtt{A}$. We extend to buffer typings the assumption for set inclusion stated for standard elements in Γ. For location typings, we assume that we can write $\Gamma, \mathtt{p}@l$ only if $\nexists\, l'$ s.t. $\mathtt{p}@l' \in \Gamma$. This requirement formalises that a process is associated only with one location.

To relate the typing of queues to the buffer types expected by the protocol of sessions, we define the *buffer type projection* $[\![G]\!]^{\mathtt{A}}_{\mathtt{B}}$, which follows the rules in Figure 7 and returns the expected buffer type of role $\mathtt{B}$ from $\mathtt{A}$ in G.

The idea behind the buffer type projection follows the one presented above for global-to-local type projection. We have types in the type G that specify global behaviour (e.g., "A communicates to B"), but we want to check the correspondence of the specification wrt a given deployment, which contains messages that are sent/wait to be received by the participants. Hence, to make sure that a given deployment conforms with the expected behaviour of its companion choreography, we generate buffer types which allow us to check that the kind and order of messages in queues agree with the global types that define the choreography.

Formally, we define $[\![G]\!]^{\mathtt{A}}_{\mathtt{B}}$ to extract from G the partial receptions of the form $\mathtt{A}\rangle\mathtt{B}.o(U)$, translating them to local types of the form $\&\mathtt{A}.o(U)$. Below, we report the rule that extends global type projection for global buffers.

$$[\![\mathtt{A}\rangle\mathtt{B}.o(U);G]\!]_{\mathtt{C}} = \begin{cases} \&\mathtt{A}.o(U);[\![G]\!]_{\mathtt{C}} & \text{if } \mathtt{C}=\mathtt{B} \\ [\![G]\!]_{\mathtt{C}} & \text{otherwise} \end{cases}$$

Note that we do not need to extend the projection to (*global choice*) and (*global branch*). Indeed, in our setting, we consider only running global types that are evolutions of a global type, hence global choices and branches are always balanced. Given a running global type G, we can always obtain an equivalent ($\simeq_{\mathtt{G}}$, $\equiv_{\mathtt{G}}$) global type G' which is absent from (*global choice*) and (*global branch*) terms. We call a running global type *canonic* if it contains no (*global choice*) and (*global branch*) terms. When writing projections of global types we assume G to be in canonic form.

Finally, we extend our typing discipline with a new rule $\lfloor^{\mathsf{T}}|_{\mathsf{DC}}\rceil$ that checks for coherence among types, choreographies, and deployments. To define $\lfloor^{\mathsf{T}}|_{\mathsf{DC}}\rceil$, we formalise a predicate, called *partial coherence*[1] and denoted $\mathbf{pco}(\Gamma)$, that holds if and only if, for all sessions k, the local and buffer typing of k follow (are projections of) the same global type G.

Definition 1 (Partial Coherence)**.** We write $\mathbf{pco}(\Gamma)$ when, for all sessions k in Γ, there exists a global type G such that

$$\forall\, k[\mathtt{B}] : T \in \Gamma,\ T = [\![G]\!]_{\mathtt{B}} \quad \wedge \quad \forall\, \mathtt{A} \in \mathbf{roles}(G) \setminus \{\mathtt{B}\},\ \Gamma \vdash k[\mathtt{A}\rangle\mathtt{B}] :\ [\![G]\!]^{\mathtt{A}}_{\mathtt{B}}$$

Rule $\lfloor^{\mathsf{T}}|_{\mathsf{DC}}\rceil$ is defined as:

$$\frac{\mathbf{pco}(\Gamma) \quad \Gamma \vdash D \quad \Gamma \vdash C}{\Gamma \vdash D, C}\ \lfloor^{\mathsf{T}}|_{\mathsf{DC}}\rceil$$

where a judgement $\Gamma \vdash D, C$ states that C and D are coherent according to Γ and all sessions in Γ are coherent. Γ is an abstraction between D and C and guarantees that D cannot go wrong. Formally

[1] Partial because it accounts for missing typing of roles implemented by external partial choreographies.

	Typing Environment	Choreography	Deployment
①	$G = \mathtt{A} \longrightarrow \mathtt{B}.pass(\mathbf{str});$<br>$\mathtt{B} \longrightarrow \mathtt{C}.fwd(\mathbf{str});$<br>$\mathsf{end}$<br>$k[\mathtt{A}] = \oplus\mathtt{B}.pass(\mathbf{str});\mathsf{end}$<br>$k[\mathtt{B}] = \&\mathtt{A}.pass(\mathbf{str});$<br>$\oplus\mathtt{C}.fwd(\mathbf{str});\mathsf{end}$<br>$k[\mathtt{C}] = \&\mathtt{B}.fwd(\mathbf{str});\mathsf{end}$	$C' = k\colon \mathsf{a}[\mathtt{A}].\text{"ok"} \longrightarrow \mathsf{b}[\mathtt{B}].pass(\ x\ );$<br>$k\colon \mathsf{b}[\mathtt{B}].x \longrightarrow \mathsf{c}[\mathtt{C}].fwd(\ x\ )$	D'
	$G \to G'$ by $\lfloor^{\mathsf{G}}\vert_{\mathsf{Eq}}\rceil, \lfloor^{\mathsf{G}}\vert_{\mathsf{Send}}\rceil$	$C' \to C''$ by $\lfloor^{\mathsf{C}}\vert_{\mathsf{Eq}}\rceil, \lfloor^{\mathsf{C}}\vert_{\mathsf{Send}}\rceil$	$\delta = k\colon \mathsf{a}[\mathtt{A}].\text{"ok"} \longrightarrow \mathtt{B}.pass$<br>$D', \delta \blacktriangleright D''$ by $\lfloor^{\mathsf{D}}\vert_{\mathsf{Send}}\rceil$
②	$G' = \mathtt{A}\rangle\mathtt{B}.pass(\mathbf{str});$<br>$\mathtt{B} \longrightarrow \mathtt{C}.fwd(\mathbf{str});$<br>$\mathsf{end}$<br>$k[\mathtt{A}] = \mathsf{end}$<br>$k[\mathtt{B}] = \&\mathtt{A}.pass(\mathbf{str});$<br>$\oplus\mathtt{C}.fwd(\mathbf{str});\mathsf{end}$<br>$k[\mathtt{C}] = \&\mathtt{B}.fwd(\mathbf{str});\mathsf{end}$<br>$k[\mathtt{A}\rangle\mathtt{B}] = \&\mathtt{A}.pass(\mathbf{str})$	$C'' = k\colon \mathtt{A} \longrightarrow \mathsf{b}[\mathtt{B}].pass(\ x\ );$<br>$k\colon \mathsf{b}[\mathtt{B}].x \longrightarrow \mathsf{c}[\mathtt{C}].fwd(\ x\ )$	$D''(k[\mathtt{A}\rangle\mathtt{B}]) = (pass, \text{"ok"})$
	$G' \to G''$ by $\lfloor^{\mathsf{G}}\vert_{\mathsf{Recv}}\rceil$	$C'' \to C'''$ by $\lfloor^{\mathsf{C}}\vert_{\mathsf{Recv}}\rceil$	$\delta' = k\colon \mathtt{A} \longrightarrow \mathsf{b}[\mathtt{B}].pass(x)$<br>$D'', \delta' \blacktriangleright D'''$ by $\lfloor^{\mathsf{D}}\vert_{\mathsf{Recv}}\rceil$
③	$G'' = \mathtt{B} \longrightarrow \mathtt{C}.fwd(\mathbf{str});$<br>$\mathsf{end}$<br>$k[\mathtt{A}] = \mathsf{end}$<br>$k[\mathtt{B}] = \oplus\mathtt{C}.fwd(\mathbf{str});\mathsf{end}$<br>$k[\mathtt{C}] = \&\mathtt{B}.fwd(\mathbf{str});\mathsf{end}$	$C''' = k\colon \mathsf{b}[\mathtt{B}].x \longrightarrow \mathsf{c}[\mathtt{C}].fwd(\ x\ )$	$D'''(\mathsf{b}).x = \text{"ok"}$

Table 1. Example of message delivery on elements of interest of choreography C' (second column), its companion deployment D' (third column), and their typing environment (first column).

Definition 2 (Deployment Judgements)**.**

$$\Gamma \vdash D \iff \begin{cases} (1)\ \forall\ \mathsf{p}.x\colon U \in \Gamma,\ D(\mathsf{p}).x : U \\ (2)\ \forall\ k[\mathtt{A}\rangle\mathtt{B}] : T \in \Gamma \land D(k[\mathtt{A}\rangle\mathtt{B}]) = \tilde{m},\ \mathtt{bte}(\mathtt{A},\tilde{m}) = T \end{cases}$$

We comment the checks performed by $\Gamma \vdash D$: (1) checks that, for each typing $\mathsf{p}.x\colon U$ in Γ, D associates x, in the state of process p, with value of type U; (2) uses buffer types to check that the typing of a message queue in Γ is correct wrt to the actual sequence of messages stored by that queue in D. We extract the type of a queue $\tilde{m}$, i.e., the sequence of message receptions from a role $\mathtt{A}$, with function $\mathtt{bte}(\mathtt{A},\tilde{m})$. We state the formal definition below.

Definition 3 (Buffer Type Extraction)**.** Let $\vdash v_i\colon U_i$, $i \in [1,n]$ and $\tilde{m} = (o_1,v_1) :: \cdots :: (o_n,v_n)$, then $\mathtt{bte}(\ \mathtt{A},\tilde{m}\ )\ =\ \&\mathtt{A}.o_1(U_1);\ \cdots;\ \&\mathtt{A}.o_n(U_n)$.

1.4 Runtime Examples, Typing and Reductions

In this section, we present two running examples that illustrate the relationship between global types and choreographies. First, we report a basic case where a session starts and two processes exchange a message. Then we consider a started session and comment on the asynchronous delivery of messages.

Example 1 (Start and Message Delivery)**.** We consider a running choreography C,D and a global type G such that D is a default deployment (cf. Definition 1 of the article) and

$$C = \begin{array}{l} \mathtt{start}\ k : \mathsf{a}[\mathtt{A}] \leftrightarrow\!\!\triangleright\ l_{\mathtt{B}}.\mathsf{b}[\mathtt{B}], l_{\mathtt{C}}.\mathsf{c}[\mathtt{C}]; \\ k\colon \mathsf{a}[\mathtt{A}].\text{"ok"} \longrightarrow \mathsf{b}[\mathtt{B}].pass(\ x\ ); \\ k\colon \mathsf{b}[\mathtt{B}].x \longrightarrow \mathsf{c}[\mathtt{C}].fwd(\ x\ ) \end{array} \qquad G = \begin{array}{l} \mathtt{A} \longrightarrow \mathtt{B}.pass(\mathbf{str}); \\ \mathtt{B} \longrightarrow \mathtt{C}.fwd(\mathbf{str}); \\ \mathsf{end} \end{array}$$

The global type G is used in the typing environment Γ to check C,D. Formally, the service typing $l_{\mathtt{B}},l_{\mathtt{C}}\colon G\langle\ \mathtt{A} \mid \mathtt{B},\mathtt{C} \mid \mathtt{B},\mathtt{C}\ \rangle$ belongs to Γ and $\Gamma \vdash C,D$.

Now, we let D, C reduce to D', C' following rules $\lfloor^{\mathsf{C}}|_{\mathsf{Start}}\rceil$ and $\lfloor^{\mathsf{D}}|_{\mathsf{Start}}\rceil$ so that D contains the data and queues needed to support interactions on session k. Finally, we report the following in Table 1:

- left column, the main elements in the typing environment Γ, i.e., the evolution of the type G. To show how partial coherence (Definition 1) holds, we report also the local and buffer types of A, B, and C projected from G following global type projection $[\![G]\!]_{\mathtt{A}}$ for local types (see Figure 3) and buffer type projection $[\![G]\!]_{\mathtt{A}}^{\mathtt{B}}$ (see Figure 7) for buffer types. For brevity, we omit empty buffer types such as $k[\mathtt{A}\rangle\mathtt{B}] = \mathsf{end}$;
- middle column, the reduction of choreography C;
- right column, the main changes in the deployment D.

To ease the reading of the example, we highlight in grey the elements that have been changed by the reduction. To keep our example brief, we only report the reduction (sending and reception) of the first interaction in C, namely $k\!:\!\mathsf{a}[\mathtt{A}].\text{"ok"} \multimap \mathsf{b}[\mathtt{B}].pass(\ x\ )$.

In Table 1, row ① shows on the left column the original type G and the global type projection onto the local types of roles A, B, and C; in the next two columns we reported for completeness the reductions C' and D'. Next, we let the running choreography reduce, applying rules $\lfloor^{\mathsf{C}}|_{\mathsf{Eq}}\rceil$, $\lfloor^{\mathsf{C}}|_{\mathsf{Send}}\rceil$, and $\lfloor^{\mathsf{D}}|_{\mathsf{Send}}\rceil$ to let process a deliver its message in the queue $k[\mathtt{A}\rangle\mathtt{B}]$ of process b. We also let G reduce to G' with rule $\lfloor^{\mathsf{G}}|_{\mathsf{Send}}\rceil$. In row ② we report the result of the reductions. In the left column, G' indicates that role A has sent a message to B, which should consume it in the next step. This is also mirrored by the buffer projection, where the buffer typing $k[\mathtt{A}\rangle\mathtt{B}]$ is $\&\mathtt{A}.pass$. The deployment D'' contains the actual message sent by a in the queue owned by b. The reduced running choreography is still well-typed as, applying function $\mathtt{bte}(\ \mathtt{A}, D''(k[\mathtt{A}\rangle\mathtt{B}])\ )$ on the interested queue, we obtain the same local type of the buffer typing $k[\mathtt{A}\rangle\mathtt{B}]$. Finally, we let the running choreography and the global type reduce again, allowing process b to consume the message. We show the result of the reductions in row ③, where in deployment D''' we can find that the value of the message has been assigned to the receiving variable x of b.

Example 2 (Asynchronous Message Delivery)**.** In this example, we consider a well-typed running choreography $\Gamma \vdash D, C$ where C and its correspondent reduced global type G are:

$$C = \begin{array}{l} k\!:\!\mathsf{a}[\mathtt{A}] \multimap \mathsf{b}[\mathtt{B}].first();\\ k\!:\!\mathsf{a}[\mathtt{A}] \multimap \mathsf{b}[\mathtt{B}].second()\end{array} \qquad G = \begin{array}{l}\mathtt{A} \multimap \mathtt{B}.first(\mathbf{unit});\\ \mathtt{A} \multimap \mathtt{B}.second(\mathbf{unit});\\ \mathsf{end}\end{array}$$

We keep the same conventions on notation defined in the previous example with the addition of omitting round parenthesis for void values. We report in Table 2 a possible sequence of reductions. Following the previous example, we use row ① to summarise the status of (from left to right) the typing environment Γ, the choreography C, and its companion deployment D.

In row Ⓐ, we report the main elements involved in the reduction. In the left-most cell of the row, the global type G_1 is structurally equivalent ($\equiv_{\mathsf{G}}$) to G and that appears in rule $\lfloor^{\mathsf{G}}|_{\mathsf{Eq}}\rceil$ to split the complete communication $\mathtt{A} \multimap \mathtt{B}.first()$ into its equivalent $\oplus\mathtt{AB}.first();\&\mathtt{AB}.first()$. Then G_1 reduces to G_1' with rule $\lfloor^{\mathsf{G}}|_{\mathsf{Send}}\rceil$ and, as of rule $\lfloor^{\mathsf{G}}|_{\mathsf{Eq}}\rceil$, we take G' as structurally equivalent to G_1', as shown in row ②, G' splits the complete communication $\mathtt{A} \multimap \mathtt{B}.second()$ into its equivalent $\oplus\mathtt{AB}.second();\&\mathtt{AB}.second()$. The reduction of C mirrors that of G: it splits the complete communication on operation $first$, consumes the sending, and finally splits the other complete communication on operation $second$, resulting in C' (row ②). The sending is applied on D which contains the related message in queue $k[\mathtt{A}\rangle\mathtt{B}]$ in its reductum D'.

Then, in row Ⓑ we allow the delivery of operation $second$. This illustrates how asynchrony works at both levels of global types and choreographies. As before, we start from the left-most cell in the row. First we consider G_2, which is swap-equivalent to G', after applying to it rule $\lfloor^{\mathsf{GS}}|_{\mathsf{ChoBuf}}\rceil$. This brings on top the (*global choice*) on operation $second$. Then G_2 reduces to G_2' with rule $\lfloor^{\mathsf{G}}|_{\mathsf{Send}}\rceil$ and, as of rule $\lfloor^{\mathsf{G}}|_{\mathsf{Eq}}\rceil$, we take $G'' = G_1'$. The reduction on C', D' is similar to that of G'.

1.5 Properties

We close this section with the main guarantees of our type system.

First, our semantics preserves well-typedness:

	Typing Environment	Choreography	Deployment
①	$G = \mathtt{A} \rightarrowtriangle \mathtt{B}.first;$<br>$\quad \mathtt{A} \rightarrowtriangle \mathtt{B}.second;$<br>$\quad \mathsf{end}$<br>$k[\mathtt{A}] = \oplus\mathtt{B}.first;$<br>$\quad \oplus\mathtt{B}.second; \mathsf{end}$<br>$k[\mathtt{B}] = \&\mathtt{A}.first;$<br>$\quad \&\mathtt{A}.second; \mathsf{end}$	$C = k : \mathsf{a}[\mathtt{A}] \rightarrowtriangle \mathsf{b}[\mathtt{B}].first;$<br>$\quad k : \mathsf{a}[\mathtt{A}] \rightarrowtriangle \mathsf{b}[\mathtt{B}].second$	D
Ⓐ	$G \to G'$ by $\lfloor^{\mathsf{G}}\vert_{\mathsf{Eq}}\rceil, \lfloor^{\mathsf{G}}\vert_{\mathsf{Send}}\rceil$ i.e.,<br>$G \equiv_{\mathsf{G}} G_1 = \oplus\mathtt{AB}.first;$<br>$\quad \&\mathtt{AB}.first;$<br>$\quad \mathtt{A} \rightarrowtriangle \mathtt{B}.second;$<br>$\quad \mathsf{end}$<br>$G_1 \to G_1'$ and $G_1' \equiv_{\mathsf{G}} G'$	$C \to C'$ by $\lfloor^{\mathsf{C}}\vert_{\mathsf{Eq}}\rceil, \lfloor^{\mathsf{C}}\vert_{\mathsf{Send}}\rceil$	$\delta = k : \mathsf{a}[\mathtt{A}] \rightarrowtriangle \mathtt{B}.first$<br>$D,\delta \blacktriangleright D'$ by $\lfloor^{\mathsf{D}}\vert_{\mathsf{Send}}\rceil$
②	$G' = \mathtt{A}\rangle\mathtt{B}.first;$<br>$\quad \oplus\mathtt{AB}.second;$<br>$\quad \&\mathtt{AB}.second;$<br>$\quad \mathsf{end}$<br>$k[\mathtt{A}] = \oplus\mathtt{B}.second; \mathsf{end}$<br>$k[\mathtt{B}] = \&\mathtt{A}.first;$<br>$\quad \&\mathtt{A}.second; \mathsf{end}$<br>$k[\mathtt{A}\rangle\mathtt{B}] = \&\mathtt{A}.first(); \mathsf{end}$	$C' = k : \mathtt{A} \rightarrowtriangle \mathsf{b}[\mathtt{B}].first;$<br>$\quad k : \mathsf{a}[\mathtt{A}] \rightarrowtriangle \mathtt{B}.second;$<br>$\quad k : \mathtt{A} \rightarrowtriangle \mathsf{b}[\mathtt{B}].second$	$D'(k[\mathtt{A}\rangle\mathtt{B}]) = (first, \_)$
Ⓑ	$G' \to G''$ by $\lfloor^{\mathsf{G}}\vert_{\mathsf{Eq}}\rceil, \lfloor^{\mathsf{G}}\vert_{\mathsf{Send}}\rceil$ i.e.,<br>$G' \simeq_{\mathsf{G}} G_2 = \oplus\mathtt{AB}.second;$<br>$\quad \mathtt{A}\rangle\mathtt{B}.first;$<br>$\quad \&\mathtt{AB}.second;$<br>$\quad \mathsf{end}$<br>$G_2 \to G_2'$ and $G_2' \simeq_{\mathsf{G}} G''$	$C' \to C''$ by $\lfloor^{\mathsf{C}}\vert_{\mathsf{Eq}}\rceil, \lfloor^{\mathsf{C}}\vert_{\mathsf{Send}}\rceil$	$\delta' = k : \mathtt{A} \rightarrowtriangle \mathsf{b}[\mathtt{B}].second$<br>$D',\delta' \blacktriangleright D''$ by $\lfloor^{\mathsf{D}}\vert_{\mathsf{Send}}\rceil$
③	$G'' = \mathtt{A}\rangle\mathtt{B}.first;$<br>$\quad \mathtt{A}\rangle\mathtt{B}.second;$<br>$\quad \mathsf{end}$<br>$k[\mathtt{A}] = \mathsf{end}$<br>$k[\mathtt{B}] = \&\mathtt{A}.first;$<br>$\quad \&\mathtt{A}.second;$<br>$\quad \mathsf{end}$<br>$k[\mathtt{A}\rangle\mathtt{B}] = \&\mathtt{A}.first$<br>$\quad \&\mathtt{A}.second$<br>$\quad \mathsf{end}$	$C'' = k : \mathtt{A} \rightarrowtriangle \mathsf{b}[\mathtt{B}].first;$<br>$\quad k : \mathtt{A} \rightarrowtriangle \mathsf{b}[\mathtt{B}].second$	$D''(k[\mathtt{A}\rangle\mathtt{B}]) = (first, \_) ::$<br>$(second, \_)$

Table 2. Example of asynchrony and effects on elements of interest of choreography C (second column), its companion deployment D (third column), and their typing environment (first column).

Theorem 1 (Subject Reduction)**.** $\Gamma \vdash D, C$ and $D, C \to D', C'$ imply $\Gamma' \vdash D', C'$ for some Γ'.

We report in Section 3.1 the proof of Theorem 1.

We now relate Γ and Γ' to prove that the behaviour of sessions in a well-typed choreography follow their respective types.

To simplify the definition of Theorem 2, we introduce a session-bound global-type projection, which associates the local types and buffer types generated from a given global type G to a given session name k. We denote $[\![G]\!]_k$ the set of local and buffer types as obtained by the projection of a global type G on each of its roles and on the pairwise queues for session k:

Definition 4 (Global Type Projection)**.**

$$[\![G]\!]_k = \{\ k[\mathtt{A}] : [\![G]\!]_{\mathtt{A}} \mid \mathtt{A} \in \mathbf{roles}(G)\ \} \cup \{\ k[\mathtt{A}\rangle\mathtt{B}] : [\![G]\!]^{\mathtt{A}}_{\mathtt{B}} \mid \mathtt{A} \in \mathbf{roles}(G), \mathtt{B} \in \mathbf{roles}(G) \setminus \{\mathtt{A}\}\ \}$$

In the definition of Theorem 2, we say that a reduction is "at session k" if it is obtained by consuming a communication term for session k (as in (Honda et al., 2016)), and we write $k \notin \Gamma$ when k does not appear in any local typing in Γ. Then we have:

Theorem 2 (Session Fidelity)**.** Let $\Gamma, \Gamma_k \vdash D, C$, $k \notin \Gamma$. Then, $D, C \rightarrow D', C'$ with a redex at session k implies that, for some G and Γ', $k \notin \Gamma'$, (i) $\Gamma_k \subseteq [\![G]\!]_k$, (ii) $G \rightarrow G'$, (iii) $\Gamma'_k \subseteq [\![G']\!]_k$, and (iv) $\Gamma', \Gamma'_k \vdash D', C'$.

Theorem 2 states that all communications on sessions follow the expected protocols (Γ' may differ from Γ for the instantiation of a new variable). The proof of Theorem 2 is reported in Section 3.1.

Finally, we present the definition of the coherence predicate **co**:

Definition 5 (Coherence)**.** $\mathbf{co}(\Gamma)$ holds iff $\forall\, k \in \Gamma$, $\exists\, G$ s.t.

- $\tilde{l} : G\langle \mathtt{A}|\tilde{\mathtt{B}}|\tilde{\mathtt{C}}\rangle \in \Gamma \;\wedge\; \tilde{\mathtt{C}} = \tilde{\mathtt{B}}$ and
- $\forall\, \mathtt{A} \in \mathbf{roles}(G), k[\mathtt{A}] : T \in \Gamma \quad \wedge \quad T = [\![G]\!]_{\mathtt{A}} \quad \wedge \quad \forall\, \mathtt{B} \in \mathbf{roles}(G) \setminus \{\mathtt{A}\}, \Gamma \vdash k[\mathtt{B}\rangle\mathtt{A}] : [\![G]\!]^{\mathtt{B}}_{\mathtt{A}}$

Coherence extends partial coherence to check that *i*) all needed services to start new sessions are present and *ii*) all the roles in every open session are correctly implemented by some processes.

Coherent and well-typed systems are deadlock-free, as stated by Theorem 3.

Theorem 3 (Deadlock-freedom)**.** $\Gamma \vdash D, C$ and $\mathbf{co}(\Gamma)$ imply that either (i) $C \equiv_{\mathtt{C}} \mathbf{0}$ or (ii) there exist D' and C' such that $D, C \rightarrow D', C'$.

We report the proof of Theorem 3 in Section 3.2.

1.6 Additional Definitions

Definition 6 (List Subset)**.** Let ε be the empty list and $\tilde{N}$, $\tilde{M}$ be two lists of elements n of the kind $\tilde{N} ::= \varepsilon \mid n, \tilde{N}'$, the predicate $\tilde{N} \subseteq \tilde{M}$ holds if $\tilde{N} = \tilde{M} = \varepsilon$ or, assuming $\tilde{N} = n, \tilde{N}'$ and $\tilde{M} = m, \tilde{M}'$ either $n = m$ and $\tilde{N}' \subseteq \tilde{M}'$ or $\tilde{N} \subseteq \tilde{M}'$.

Definition 7 (Ordered Join Operator)**.** Let $\tilde{N}$, $\tilde{L}$, and $\tilde{M}$ be three lists of elements as defined in Definition 6, the ordered-join operator $\tilde{N} \bowtie_{\tilde{L}} \tilde{N}$ is defined as

$$\tilde{N} \bowtie_{\varepsilon} \tilde{M} \quad = \quad \varepsilon$$

$$\tilde{N} \bowtie_{l,\tilde{L}} \tilde{M} \quad = \quad \begin{cases} \tilde{N} \bowtie_{\tilde{L}} \tilde{M} & \text{if } l \notin \tilde{N} \cup \tilde{M} \\ l, \tilde{N}' \bowtie_{\tilde{L}} \tilde{M} & \text{if } \tilde{N} = l, \tilde{N}' \\ l, \tilde{N} \bowtie_{\tilde{L}} \tilde{M}' & \text{if } \tilde{M} = l, \tilde{M}' \end{cases}$$

2 COMPILING FRONTEND CHOREOGRAPHIES INTO DCC PROCESSES

3 PROOFS

3.1 Proofs of Subject Reduction and Session Fidelity

In order to prove Subject Reduction (Theorem 1), we prove the stronger result of Typing Soundness, defined in Theorem 4. We use Theorem 4 to also prove Session Fidelity (Theorem 2).

In order to define and prove Theorem 4, we provide additional definitions and lemmas, in particular:

- we define an annotated semantics for FC (Section 3.1.1) to track reductions on sessions;
- we define subtyping (Section 3.1.2) for local types and for typing environments. On these definitions we prove lemmas used to relate evolutions of the typing environment wrt reductions in choreographies;
- we define an annotated semantics for global types (Section 3.1.3) and prove Lemma 4, guaranteeing that global types and local types in the typing environment evolve accordingly.

Finally, we proceed to prove Typing Soundness (Section 3.1.5) and consequently Subject Reduction and Session Fidelity.

3.1.1 FC Annotated Semantics

We define the semantics of annotated FCs by marking transitions with the name of the session whose term has reduced. We annotate other reductions as τ. We range over annotated labels with

$$\beta \quad ::= \quad k\!:\!\mathtt{A} \multimap\!\!\!\rhd \mathtt{B}.o \quad \mid \quad k : \mathtt{A}\rangle\mathtt{B}.o(x) \quad \mid \quad \tau$$

We report the annotated semantics of FC in Figure 10. Intuitively, we mark reductions over a session k with $k\!:\!\mathtt{A} \multimap\!\!\!\rhd \mathtt{B}.o$ for message sends ($\lfloor^{\mathtt{C}}|_{\mathsf{Send}}\rceil$ and $\lfloor^{\mathtt{C}}|_{\mathsf{Com}}\rceil$) and $k : \mathtt{A}\rangle\mathtt{B}.o(x)$ for receptions ($\lfloor^{\mathtt{C}}|_{\mathsf{Recv}}\rceil$).

$$\begin{array}{rcl}
\begin{array}{r}\mathsf{acc}\ k : l.\mathsf{p}[\mathtt{A}];C_1 \sqcup \\ \mathsf{acc}\ k : l.\mathsf{q}[\mathtt{A}];C_2\end{array} & = & \mathsf{acc}\ k : l.\mathsf{p}[\mathtt{A}];(C_1 \sqcup C_2) \\[2em]
\begin{array}{r}\mathsf{req}\ k : \mathsf{p}[\mathtt{A}] \Leftrightarrow \widetilde{l.\mathtt{B}};C_1 \sqcup \\ \mathsf{req}\ k : \mathsf{q}[\mathtt{A}] \Leftrightarrow \widetilde{l.\mathtt{B}};C_2\end{array} & = & \mathsf{req}\ k : \mathsf{p}[\mathtt{A}] \Leftrightarrow \widetilde{l.\mathtt{B}};(C_1 \sqcup C_2) \\[2em]
\begin{array}{r}k : \mathsf{p}[\mathtt{A}].e \longrightarrow \mathtt{B}.o;C_1 \sqcup \\ k : \mathsf{q}[\mathtt{A}].e \longrightarrow \mathtt{B}.o;C_2\end{array} & = & k : \mathsf{p}[\mathtt{A}].e \longrightarrow \mathtt{B}.o;(C_1 \sqcup C_2) \\[2em]
\begin{array}{r}k : \mathtt{A} \longrightarrow \mathsf{p}[\mathtt{B}].\{\ o_i(x_i);C_i\ \}_{i \in I} \sqcup \\ k : \mathtt{A} \longrightarrow \mathsf{q}[\mathtt{B}].\{\ o_j(x_j);C'_j\ \}_{j \in J}\end{array} & = & k : \mathtt{A} \longrightarrow \mathsf{p}[\mathtt{B}].\left\{\begin{array}{ll} & \{\ o_i(x_i);C_i\ \}_{i \in I \setminus J} \\ \cup & \{\ o_i(x_i);C'_i\ \}_{i \in J \setminus I} \\ \cup & \{\ o_i(x_i);C_i \sqcup C'_i\ \}_{i \in I \cap J}\end{array}\right\} \\[2em]
\begin{array}{r}\mathtt{if}\ \mathsf{p}.e\ \{C_1\}\ \mathtt{else}\ \{C'_1\} \sqcup \\ \mathtt{if}\ \mathsf{q}.e\ \{C_2\}\ \mathtt{else}\ \{C'_2\}\end{array} & = & \mathtt{if}\ \mathsf{p}.e\ \{C_1 \sqcup C_2\}\ \mathtt{else}\ \{C'_1 \sqcup C'_2\} \\[2em]
\begin{array}{r}\mathsf{def}\ X = C'_1\ \mathsf{in}\ C_1 \sqcup \\ \mathsf{def}\ Y = C'_2\ \mathsf{in}\ C_2\end{array} & = & \mathsf{def}\ X = C'_1 \sqcup C'_2\ \mathsf{in}\ C_1 \sqcup C_2 \\[2em]
X \sqcup Y & = & X \\[2em]
\mathbf{0} \sqcup \mathbf{0} & = & \mathbf{0}
\end{array}$$

Figure 8. Merging Function

$$\begin{array}{lcl}
\left\lfloor \mathsf{start}\ k : \mathsf{p}[\mathtt{D}] \Leftrightarrow \widetilde{l.\mathsf{q}[\mathtt{B}]};C \right\rfloor_l & = & \left\lfloor \mathsf{acc}\ k : \widetilde{l.\mathsf{q}[\mathtt{B}]};C \right\rfloor_l \\
\left\lfloor \mathsf{acc}\ k : \widetilde{l.\mathsf{q}[\mathtt{B}]};C \right\rfloor_l & = & \begin{cases} \{\mathsf{r}\} \cup \lfloor C \rfloor_l & \text{if } l.\mathsf{r}[\mathtt{A}] \in \{\ \widetilde{l.\mathsf{q}[\mathtt{B}]}\ \} \\ \lfloor C \rfloor_l & \text{otherwise} \end{cases} \\
\lfloor \eta;C \rfloor_l & = & \lfloor C \rfloor_l \quad \text{if } \eta \neq (\mathit{start}) \\
\lfloor \mathtt{if}\ \mathsf{p}.e\ \{C_1\}\ \mathtt{else}\ \{C_2\} \rfloor_l & = & \lfloor C_1 \rfloor_l \cup \lfloor C_2 \rfloor_l \\
\lfloor \mathsf{def}\ X = C'\ \mathsf{in}\ C \rfloor_l & = & \lfloor C' \rfloor_l \cup \lfloor C \rfloor_l \\
\lfloor X \rfloor_l & = & \varnothing \\
\lfloor \mathbf{0} \rfloor_l & = & \varnothing \\
\lfloor C_1 \mid C_2 \rfloor_l & = & \lfloor C_1 \rfloor_l \cup \lfloor C_2 \rfloor_l
\end{array}$$

Figure 9. Service Grouping

$$\frac{D\#k', \tilde{\mathsf{r}} \quad \delta = \mathtt{start}\ k' : \mathsf{p}[\mathtt{A}] \leftrightarrow \widetilde{l.\mathsf{q}[\mathtt{B}]} \quad D,\delta \blacktriangleright D'}{D,\ \mathtt{start}\ k : \mathsf{p}[\mathtt{A}] \leftrightarrow \widetilde{l.\mathsf{q}[\mathtt{B}]}; C \quad \xrightarrow{\tau} \quad D',\ C[k'/k][\tilde{\mathsf{r}}/\tilde{\mathsf{q}}]}\ \lfloor^{\mathsf{C}}|_{\mathsf{Start}}\rceil$$

$$\frac{\eta = k\!:\mathsf{p}[\mathtt{A}].e \longrightarrow \mathtt{B}.o \quad D,\eta \blacktriangleright D'}{D,\ \eta; C \quad \xrightarrow{k:\,\mathtt{A} \longrightarrow \mathtt{B}.o} \quad D',\ C}\ \lfloor^{\mathsf{C}}|_{\mathsf{Send}}\rceil$$

$$\frac{j \in I \quad D, k\!:\mathtt{A} \longrightarrow \mathsf{q}[\mathtt{B}].o_j(x_j) \blacktriangleright D'}{D,\ k\!:\mathtt{A} \longrightarrow \mathsf{q}[\mathtt{B}].\{o_i(x_i); C_i\}_{i\in I} \quad \xrightarrow{k:\mathtt{A}\rangle \mathtt{B}.o_j(x_j)} \quad D',\ C_j}\ \lfloor^{\mathsf{C}}|_{\mathsf{Recv}}\rceil$$

$$\frac{i = 1 \text{ if } \mathbf{eval}(\ e, D(\mathsf{p})\ ) = \mathtt{true}, i = 2 \text{ otherwise}}{D,\ \mathtt{if}\ \mathsf{p}.e\ \{C_1\}\ \mathtt{else}\ \{C_2\} \quad \xrightarrow{\tau} \quad D,\ C_i}\ \lfloor^{\mathsf{C}}|_{\mathsf{Cond}}\rceil$$

$$\frac{D, C_1 \xrightarrow{\beta} D', C_1'}{D, \mathsf{def}\ X = C_2\ \mathsf{in}\ C_1 \quad \xrightarrow{\beta} \quad D', \mathsf{def}\ X = C_2\ \mathsf{in}\ C_1'}\ \lfloor^{\mathsf{C}}|_{\mathsf{Ctx}}\rceil$$

$$\frac{\mathcal{R} \in \{\equiv, \simeq_{\mathsf{C}}\} \quad C\,\mathcal{R}\,C_1 \quad D, C_1 \xrightarrow{\beta} D', C_1' \quad C_1\,\mathcal{R}\,C'}{D, C \quad \xrightarrow{\beta} \quad D', C'}\ \lfloor^{\mathsf{C}}|_{\mathsf{Eq}}\rceil$$

$$\frac{D, C_1 \quad \xrightarrow{\beta} \quad D', C_1'}{D, C_1 \mid C_2 \quad \xrightarrow{\beta} \quad D', C_1' \mid C_2}\ \lfloor^{\mathsf{C}}|_{\mathsf{Par}}\rceil$$

$$\frac{\begin{array}{c} i \in \{1,\ldots,n\} \quad D\#k', \tilde{\mathsf{r}} \quad \{\widetilde{l.\mathtt{B}}\} = \biguplus_i \{\widetilde{l_i.\mathtt{B}_i}\} \quad \{\tilde{\mathsf{r}}\} = \bigcup_i \{\tilde{\mathsf{r}}_i\} \\ \delta = \mathtt{start}\ k' : \mathsf{p}[\mathtt{A}] \leftrightarrow \widetilde{l_1.\mathsf{r}_1[\mathtt{B}_1]}, \ldots, \widetilde{l_n.\mathsf{r}_n[\mathtt{B}_n]} \quad D,\delta \blacktriangleright D' \end{array}}{\begin{array}{l} D, \mathsf{req}\ k : \mathsf{p}[\mathtt{A}] \leftrightarrow \widetilde{l.\mathtt{B}}; C \mid \prod_i \left(\mathsf{acc}\ k : \widetilde{l_i.\mathsf{q}_i[\mathtt{B}_i]}; C_i\right) \xrightarrow{\tau} \\ \quad D',\ C[k'/k] \mid \prod_i \left(\ C_i[k'/k][\tilde{\mathsf{r}}_i/\tilde{\mathsf{q}}_i]\ \right) \mid \prod_i \left(\mathsf{acc}\ k : \widetilde{l_i.\mathsf{q}_i[\mathtt{B}_i]}; C_i\right) \end{array}}\ \lfloor^{\mathsf{C}}|_{\mathsf{PStart}}\rceil$$

Figure 10. Fronted Choreographies, annotated semantics.

3.1.2 Local Types and Typing Environment Subtyping

We define a subtyping relation on local types following Gay and Hole (2005); Carbone et al. (2012); Montesi and Yoshida (2013). We write the subtyping relation as $T' \prec T$, which intuitively indicates that T' is more constrained than T in its behaviour. Note that, like in Carbone et al. (2012); Montesi and Yoshida (2013), the input type is covariant and the output type is contravariant for this relation.

Definition 8 (Local Subtyping)**.** We define the subtyping relation between local types as $T' \prec T$, which is the smallest relation over closed local types, satisfying the rules

$$\frac{T'' \prec T' \quad T \approx T''}{T \prec T'} \lfloor^{\mathsf{SubT}}|_{\mathsf{Eq}}\rceil \qquad \frac{J \subseteq I \quad \forall\, i \in J \mid T_i \prec T'_i \;\wedge\; U_i \prec U'_i}{!\mathtt{A}.\{o_i(U_i);T_i\}_{i\in I} \prec\, !\mathtt{A}.\{o_i(U'_i);T'_i\}_{i\in J}} \lfloor^{\mathsf{SubT}}|_{\mathsf{Send}}\rceil$$

$$\frac{I \subseteq J \quad \forall\, i \in I \mid T_i \prec T'_i \;\wedge\; U_i \prec U'_i}{?\mathtt{A}.\{o_i(U_i);T_i\}_{i\in I} \prec\, ?\mathtt{A}.\{o_i(U'_i);T'_i\}_{i\in J}} \lfloor^{\mathsf{SubT}}|_{\mathsf{Recv}}\rceil \qquad \frac{}{U \prec U} \lfloor^{\mathsf{SubT}}|_{\mathsf{Val}}\rceil \qquad \frac{\mathsf{end} \approx T}{\mathsf{end} \prec T} \lfloor^{\mathsf{SubT}}|_{\mathsf{End}}\rceil$$

In rule $\lfloor^{\mathsf{SubT}}|_{\mathsf{Eq}}\rceil$, $T \prec T'$ if there exists a local type T'', subtype of T', such that $T \approx T''$, i.e., T'' approximates T, $\approx$ being the standard tree isomorphism on recursive types.

Although not directly relevant in the current proof, we also define the subtyping for global types $G \prec G'$, which intuitively follows that of local ones. Subtyping for global types is used in the definition of Environment subtyping. The relation between subtyping of Environments and of global types (in service typings) will become relevant when proving properties of our Endpoint Projection (see Section 3.4). Our definition of subtyping for global types follows Montesi and Yoshida (2013).

Definition 9 (Global Subtyping)**.** $G \prec G'$ is the smallest relation over closed global types satisfying the rules below

$$\frac{I \subseteq J \quad \forall\, i \in I,\, G_i \prec G'_i \;\wedge U_i \prec U'_i}{\mathtt{A} \longrightarrow \mathtt{B}.\{o_i(U_i);G_i\}_{i\in I} \prec \mathtt{A} \longrightarrow \mathtt{B}.\{o_j(U'_j);G'_j\}_{j\in J}} \lfloor^{\mathsf{SubG}}|_{\mathsf{Com}}\rceil$$

$$\frac{U \prec U' \quad G \prec G'}{\mathtt{A}\rangle\mathtt{B}.o(U);G \prec \mathtt{A}\rangle\mathtt{B}.o(U');G'} \lfloor^{\mathsf{SubG}}|_{\mathsf{Recv}}\rceil$$

$$\frac{G'' \prec G' \quad (G'' \approx G \;\vee\; G'' \simeq_{\mathsf{G}} G)}{G \prec G'} \lfloor^{\mathsf{SubG}}|_{\mathsf{Eq}}\rceil \qquad \frac{\mathsf{end} \approx G}{\mathsf{end} \prec G} \lfloor^{\mathsf{SubG}}|_{\mathsf{End}}\rceil$$

Finally, we define a subtyping relation between Typing Environments. Intuitively $\Gamma \prec \Gamma'$ means that Γ' and Γ are identical Typing Environments up to *a*) some local and global types that are more constrained in Γ—i.e., subtypes of a correspondent global/local type—than in Γ' and *b*) some service typings not present in Γ.

Definition 10 (Typing Environment Subtyping)**.** Let Γ and Γ' be two typing environments, where $\Gamma' = \Gamma'', \Gamma_l$, for which $\mathbf{dom}(\Gamma) = \mathbf{dom}(\Gamma'')$ and Γ_l contains only service typings. Then, $\Gamma \prec \Gamma'$ if and only if

(*i*)	$\forall\, \mathsf{p}.x : U \in \Gamma,$	$\Gamma' \vdash \mathsf{p}.x : U$
(*ii*)	$\forall\, X : \Gamma_x \in \Gamma,$	$\Gamma' \vdash X : \Gamma_x$
(*iii*)	$\forall\, \mathsf{p} : k[\mathtt{A}] \in \Gamma,$	$\Gamma' \vdash \mathsf{p} : k[\mathtt{A}]$
(*iv*)	$\forall\, \mathsf{p}@l \in \Gamma,$	$\Gamma' \vdash \mathsf{p}@l$
(*v*)	$\forall\, k[\mathtt{A}\rangle\mathtt{B}] : T \in \Gamma,$	$\Gamma' \vdash k[\mathtt{A}\rangle\mathtt{B}] : T$
(*vi*)	$\forall\, k[\mathtt{A}] : T \in \Gamma,$	$\Gamma' \vdash k[\mathtt{A}] : T'$ and $T \prec T'$
(*vii*)	$\forall\, \tilde{l} : G\langle \mathtt{A}\vert\tilde{\mathtt{B}}\vert\tilde{\mathtt{C}}\rangle \in \Gamma,$	$\Gamma' \vdash \tilde{l} : G'\langle \mathtt{A}\vert\tilde{\mathtt{B}}\vert\tilde{\mathtt{C}}\rangle$ and $G \prec G'$

Commenting the definition, the subtyping relation for typing environments states that an environment Γ is a subtype of an environment Γ' if

- they type the same variables (*i*), procedure definitions (*ii*), role ownerships (*iii*), process locations (*iv*), and buffers (*v*) and they agree on their judgements;

- they type the same local sessions (*vi*) and the local type in Γ is a subtype of the local type in Γ';

- if they type the same service (*vii*) (note that Γ' is allowed to have additional service typings wrt Γ) and the global type in Γ is a subtype of the global type in Γ'.

In Lemma 1 we prove that if $\Gamma \prec \Gamma'$ and Γ types a running choreography D, C also Γ' types that choreography.

Lemma 1 (Subsumption)**.** Let $\Gamma \prec \Gamma'$ and $\Gamma \vdash D, C$ for some D, C then $\Gamma' \vdash D, C$.

Proof The proof is immediate by Definition 8 and rules $\lfloor^{\mathsf{T}}|_{\mathsf{Recv}}\rceil$, $\lfloor^{\mathsf{T}}|_{\mathsf{Send}}\rceil$, and $\lfloor^{\mathsf{T}}|_{\mathsf{Com}}\rceil$. Intuitively, the lemma holds since the local typings in Γ' allow for additional, unused actions in D, C. □

We also prove Lemma 2 which guarantees that the typing of choreographies (C) is invariant wrt buffer types.

Lemma 2 (Buffer types invariance)**.** Let $\Gamma = \Gamma', \Gamma_b$ where Γ_b contains only buffer typings. If $\Gamma' \vdash C$ then $\Gamma \vdash C$.

Proof Trivial from the definition of rule $\lfloor^{\mathsf{T}}|_{\mathsf{DC}}\rceil$ and $\Gamma \vdash C$ for which buffer typings affect only predicate **pco** and the typing of deployments. □

3.1.3 Reductions for Global Types

We annotate the reductions of global types with labels

$$\gamma \quad ::= \quad \mathtt{A} \longrightarrow\!\!\triangleright\, \mathtt{B}.o \quad \mid \quad \mathtt{A}\rangle\mathtt{B}.o$$

and report below the correspondent annotated semantics.

$$\frac{o \in \bigcup_i \{o_i\} \quad G' = {}^{\mathtt{A}\rangle\mathtt{B}}_{\;o}{\downarrow} G}{\oplus \mathtt{AB}.\{o_i(U_i)\}; G \xrightarrow{\mathtt{A} \longrightarrow\triangleright \mathtt{B}.o} G'} \lfloor^{\mathsf{G}}|_{\mathsf{Send}}\rceil \qquad \frac{}{\mathtt{A}\rangle\mathtt{B}.o(U); G \xrightarrow{\mathtt{A}\rangle\mathtt{B}.o} G} \lfloor^{\mathsf{G}}|_{\mathsf{Recv}}\rceil$$

$$\frac{\mathcal{R} \in \{\equiv_{\mathtt{G}}, \simeq_{\mathtt{G}}\} \quad G\,\mathcal{R}\,G_1 \quad G_1 \xrightarrow{\gamma} G_1' \quad G_1'\,\mathcal{R}\,G'}{G \xrightarrow{\gamma} G'} \lfloor^{\mathsf{G}}|_{\mathsf{Eq}}\rceil$$

In Lemma 3 we account for the fact that any output reduction at the level of global types can constrain the projected local types of the roles not involved in the reduction. Indeed, referring to rule $\lfloor^{\mathsf{G}}|_{\mathsf{Send}}\rceil$, the output operation chooses one of the available continuations G' and discards all the others. Therefore the local types of the other roles not involved in the reduction can be constrained by the removal of the unused branches.

Lemma 3 (Projection Subtyping)**.** Let $T = [\![G]\!]_{\mathtt{C}}$, $T' = [\![G']\!]_{\mathtt{C}}$, and $\{\mathtt{A},\mathtt{B},\mathtt{C}\} \subseteq \mathbf{roles}(G)$, $\mathtt{C} \notin \{\mathtt{A},\mathtt{B}\}$, then $G \xrightarrow{\mathtt{A} \longrightarrow\triangleright \mathtt{B}.o} G'$ implies $T' \prec T$.

Proof By induction on the derivation of $G \xrightarrow{\gamma} G'$. □

3.1.4 Typing Environment Reductions

We define a reduction relation for typing environments. To do so, we first formalise the writing $k \notin \Gamma$, which means that Γ has no local typing and buffer types for session k, formally, for some local types T and T'

$$k \notin \Gamma \iff \nexists\, \mathtt{A},\mathtt{B} \text{ s.t. } k[\mathtt{A}] : T \in \Gamma \;\vee k[\mathtt{A}\rangle\mathtt{B}] : T' \in \Gamma$$

Finally, we formalise the reduction relation for typing environments of the form $\Gamma \to \Gamma'$, $\to$ being the smallest closed under the rules below. Note that the annotation labels are a subset of the labels used to annotate the semantics of FC, ranged over by β.

$$\frac{k \notin \Gamma \quad \Gamma_k \subseteq [\![G]\!]_k \quad \{k[\mathtt{A}]: T, k[\mathtt{B}]: T'\} \in \Gamma_k \quad j \in I \quad G \xrightarrow{\mathtt{A} \longrightarrow\!\!\triangleright\, \mathtt{B}.o_j} G'}{\Gamma, \Gamma_k \xrightarrow{k\colon \mathtt{A} \longrightarrow\!\!\triangleright\, \mathtt{B}.o_j} \Gamma, \{k[\mathtt{C}]\colon [\![G']\!]_{\mathtt{C}} \mid k[\mathtt{C}] \in \Gamma_k\}, \{k[\mathtt{C}\rangle\mathtt{D}]\colon [\![G']\!]^{\mathtt{D}}_{\mathtt{C}} \mid k[\mathtt{C}\rangle\mathtt{D}] \in \Gamma_k\}} \lfloor \Gamma|_{\mathsf{Send}} \rceil$$

$$\frac{k \notin \Gamma \quad \Gamma_k \subseteq [\![G]\!]_k \quad \{k[\mathtt{A}]: T, k[\mathtt{B}]: T'\} \in \Gamma_k \quad \Gamma \vdash \mathsf{q}\colon k[\mathtt{B}] \quad G \xrightarrow{\mathtt{A}\rangle\mathtt{B}.o_j} G'}{\Gamma, \Gamma_k \xrightarrow{k\colon\mathtt{A}\rangle\mathtt{B}.o_j(x)} \Gamma, \{k[\mathtt{C}]\colon [\![G']\!]_{\mathtt{C}} \mid k[\mathtt{C}] \in \Gamma_k\}, \{k[\mathtt{C}\rangle\mathtt{D}]\colon [\![G']\!]^{\mathtt{D}}_{\mathtt{C}} \mid k[\mathtt{C}\rangle\mathtt{D}] \in \Gamma_k\}, \mathsf{q}.x\colon U_j} \lfloor \Gamma|_{\mathsf{Recv}} \rceil$$

With slight abuse of notation, we also write β_k to mark reductions of Γ on session k, i.e., $\beta_k \in \{k\colon \mathtt{A} \longrightarrow\!\!\triangleright\, \mathtt{B}.o,\ k : \mathtt{A}\rangle\mathtt{B}.o(x)\}$.

We define the correspondence operator $G_{\mathsf{act}}(\beta)$ between β and γ labels:

$$G_{\mathsf{act}}(\beta_k) = \begin{cases} \mathtt{A} \longrightarrow\!\!\triangleright\, \mathtt{B}.o & \text{if } \beta_k = k\colon \mathtt{A} \longrightarrow\!\!\triangleright\, \mathtt{B}.o \\ \mathtt{A}\rangle\mathtt{B}.o & \text{if } \beta_k = k : \mathtt{A}\rangle\mathtt{B}.o(x). \end{cases}$$

In Lemma 4 we prove that if a typing environment Γ includes local types that are projection of a global type G, then if the global type can reduce, also the typing environment can reduce. The reduction preserves the correspondence between the reduced global type and the reduced local types in Γ.

Lemma 4 (Type-Environment Fidelity)**.** Let $\Gamma = \Gamma_*, [\![G]\!]_k$ for some Γ_*, $k \notin \Gamma_*$, and $G \xrightarrow{G_{\mathsf{act}}(\beta_k)} G'$ then $\Gamma \xrightarrow{\beta_k} \Gamma'$ and for some Γ'_*, $k \notin \Gamma'_*$, $\Gamma' = \Gamma'_*, [\![G']\!]_k$.

Proof Direct by cases on the derivation of Γ. ☐

3.1.5 Proof of Typing Soundness

We also report Lemmas 5 and 6 that prove that typing is invariant wrt structural equivalence and swapping.

Lemma 5 (Subject Congruence)**.** $\Gamma \vdash D, C$ and $C \equiv_{\mathtt{C}} C'$ imply $\Gamma \vdash D, C'$ (up to α-renaming)

Proof By induction on the rules that define $\equiv_{\mathtt{C}}$. ☐

Lemma 6 (Subject Swap)**.** $\Gamma \vdash D, C$ and $C \simeq_{\mathtt{C}} C'$ imply $\Gamma \vdash D, C'$

Proof By induction on the derivation of $C \simeq_{\mathtt{C}} C'$. ☐

Below we restate the definition of *Deployment Judgements* enriched with pointers of the kind $(\mathrm{DX.Y})$ for a clearer referencing in the proofs.

Definition 2 (Deployment Judgements)
$\Gamma \vdash D \iff$

(D|2.1) $\forall\ \mathsf{p}.x \in \Gamma, D(\mathsf{p}).x : U$

(D|2.2) $\forall\ k[\mathtt{A}\rangle\mathtt{B}] : T \in \Gamma \wedge D(k[\mathtt{A}\rangle\mathtt{B}]) = \tilde{m},\ \mathtt{bte}(\mathtt{A}, \tilde{m}) = T$

Finally, we prove Theorem 1 by proving the stronger result of Theorem 4.
In the proof, we use the context over global types $\mathcal{G}[\cdot]$, defined as

$$\begin{aligned} \mathcal{G}[\cdot] ::= \quad & \mathtt{A} \longrightarrow\!\!\triangleright\, \mathtt{B}.\{o_i(U_i); \mathcal{G}[\cdot]\}_i \\ \mid \quad & \oplus\mathtt{AB}.\{o_i(U_i)\}; \mathcal{G}[\cdot] \\ \mid \quad & \&\mathtt{AB}.\{o_i(U_i); \mathcal{G}[\cdot]\}_{i \in I} \\ \mid \quad & \mathtt{A}\rangle\mathtt{B}.o(U); \mathcal{G}[\cdot] \end{aligned}$$

We can now proceed to state and prove Theorem 4.

Theorem 4 (Typing Soundness)**.** Let D, C be an annotated FC and $(\mathrm{T}|4.1)$ $\Gamma \vdash D, C$ for some Γ:

if $(\mathrm{T}|4.2)$ $\beta \neq \tau$ and $D, C \xrightarrow{\beta} D', C'$ then $(\mathrm{T}|4.3)$ $\Gamma \xrightarrow{\beta} \Gamma'$ and $(\mathrm{T}|4.4)$ $\Gamma' \vdash D', C'$;
if $(\mathrm{T}|4.5)$ $D, C \xrightarrow{\tau} D', C'$ then, for some Γ', $(\mathrm{T}|4.6)$ $\Gamma' \vdash D', C'$.

Proof Proof by induction on the derivation of $D,C \xrightarrow{\beta} D',C'$.

Case $\lfloor^{\mathsf{C}}|_{\mathsf{Send}}\rceil$

The case is:

$$\frac{\eta = k\colon \mathtt{p}[\mathtt{A}].e \longrightarrow\!\triangleright \mathtt{B}.o_j \qquad D,\eta \blacktriangleright D'}{D,\ \eta;C \quad \xrightarrow{k\colon \mathtt{A} \longrightarrow\!\triangleright \mathtt{B}.o_j} \quad D',\ C}\ \lfloor^{\mathsf{C}}|_{\mathsf{Send}}\rceil$$

Where (T|4.2) has the reductum $C' = C$ and, let $v = \mathbf{eval}(e, D(\mathtt{p}))$ and $\tilde{m} = D(k[\mathtt{A}\rangle\mathtt{B}])$, $D' = D\big[\ k[\mathtt{A}\rangle\mathtt{B}] \mapsto \tilde{m} :: (o_j, v)\ \big]$ by rule $\lfloor^{\mathsf{D}}|_{\mathsf{Send}}\rceil$.

To prove (T|4.3) we must prove rule $\lfloor^{\Gamma}|_{\mathsf{Send}}\rceil$ to be applicable.

From (T|4.1) we know that there exists a global type G for session k such that $\mathbf{pco}(\Gamma)$ holds. We can partition $\Gamma = \Gamma_*, \Gamma_k$ such that $\Gamma_* = \Gamma \setminus [\![G]\!]_k$ and $\Gamma_k = \Gamma \setminus \Gamma_*$.

From (T|4.1) we can write the derivation (with $\Gamma = \Gamma_1, k[\mathtt{A}]\colon \oplus\, \mathtt{B}.\{o_i(U_i);[\![G_i]\!]_{\mathtt{A}}\}_{i\in I}$)

$$\dfrac{\mathbf{pco}(\Gamma) \qquad \Gamma \vdash D \qquad \dfrac{j \in I \qquad \Gamma_1 \vdash \mathtt{p}\colon k[\mathtt{A}] \qquad \Gamma_1 \vdash \mathtt{p}.e\colon U_j \qquad \Gamma_1, k[\mathtt{A}]\colon\ [\![G_j]\!]_{\mathtt{A}} \vdash C}{\Gamma_1, k[\mathtt{A}]\colon \oplus\, \mathtt{B}.\{o_i(U_i);[\![G_i]\!]_{\mathtt{A}}\}_{i\in I} \vdash k\colon \mathtt{p}[\mathtt{A}].e \longrightarrow\!\triangleright \mathtt{B}.o_j;C}\ \lfloor^{\mathsf{T}}|_{\mathsf{Send}}\rceil}{\Gamma \vdash D, k\colon \mathtt{p}[\mathtt{A}].e \longrightarrow\!\triangleright \mathtt{B}.o_j;C}\ \lfloor^{\mathsf{T}}|_{\mathsf{DC}}\rceil$$

Since $\Gamma \vdash k[\mathtt{A}]\colon \oplus\, \mathtt{B}.\{o_i(U_i);T_i\}_{i\in I}$, we can write $G = \mathcal{G}[\mathtt{A} \longrightarrow\!\triangleright \mathtt{B}.o_i(U_i);G_i]$ where $\forall\ i \in I$, $[\![G_i]\!]_{\mathtt{A}} = T_i$. Let π be the reduction of G with rules $\lfloor^{\mathsf{G}}|_{\mathsf{Eq}}\rceil$ and $\lfloor^{\mathsf{G}}|_{\mathsf{Send}}\rceil$, we observe the following derivation:

$$\pi = \left\{ \dfrac{G \equiv_{\mathsf{G}} G_1 \qquad \dfrac{G_1 \simeq_{\mathsf{G}} G_2 \qquad \dfrac{o_i \in U_i\{o_i\} \qquad G' = \Delta}{G_2 \xrightarrow{\gamma} G'}\ \lfloor^{\mathsf{G}}|_{\mathsf{Send}}\rceil \qquad G' \simeq_{\mathsf{G}} G'}{G_1 \xrightarrow{\gamma} G'}\ \vdots\ \lfloor^{\mathsf{G}}|_{\mathsf{Eq}}\rceil \qquad G \equiv_{\mathsf{G}} G'}{G \xrightarrow{\gamma} G'}\ \lfloor^{\mathsf{G}}|_{\mathsf{Eq}}\rceil \right. \quad \begin{array}{|l} \Delta = {}^{\mathtt{A}\rangle\mathtt{B}}_{o_i}\!\downarrow\mathcal{G}[\&_{\mathtt{A}}\mathtt{B}.\{o_i(U_i\};G_i] \\ \\ G_2 = \oplus\mathtt{A}\mathtt{B}.\{o_i(U_i)\};\mathcal{G}[\&_{\mathtt{A}}\mathtt{B}.\{o_i(U_i\};G_i] \\ G_1 = \mathcal{G}[\oplus\mathtt{A}\mathtt{B}.\{o_i(U_i\};\&_{\mathtt{A}}\mathtt{B}.\{o_i(U_i\};G_i] \\ \\ G' = \mathcal{G}[\mathtt{A}\rangle\mathtt{B}.o_j;G_j] \\ \gamma = \mathtt{A} \longrightarrow\!\triangleright \mathtt{B}.o_j \end{array}$$

In the reductions, since C, D reduces with $\beta = k\colon \mathtt{A} \longrightarrow\!\triangleright \mathtt{B}.o_j$ and G types C, D in Γ, there are no other exchanges from $\mathtt{A}$ to $\mathtt{B}$ in G that could prevent from obtaining, after a finite number of derivations on rule $\lfloor^{\mathsf{G}}|_{\mathsf{Eq}}\rceil$, the swap-equivalence $G_1 \simeq_{\mathsf{G}} G_2$. Following a similar reasoning, the application Δ targets the global branching in the context, which reduces the continuation $\mathcal{G}[\&_{\mathtt{A}}\mathtt{B}.\{o_i(U_i\};G_i]$ after the global choice $\oplus\mathtt{A}\mathtt{B}.\{o_i(U_i)\}$ to G'.

Given π, we can use it to write the reduction at the level the typing environment Γ, applying rule $\lfloor^{\Gamma}|_{\mathsf{Send}}\rceil$. Below, we consider $\Gamma = \Gamma_*, \Gamma_k$ where Γ_k contains all and only typings of session k in Γ.

$$\dfrac{k \notin \Gamma_* \qquad \Gamma_k \subseteq [\![G]\!]_k \qquad \{k[\mathtt{A}]\colon T, k[\mathtt{B}]\colon T'\} \in \Gamma_k \qquad j \in I \qquad \overset{\pi}{\vdots}\ G \xrightarrow{\mathtt{A} \longrightarrow\!\triangleright \mathtt{B}.o_j} G'}{\Gamma_*, \Gamma_k \xrightarrow{k\colon \mathtt{A} \longrightarrow\!\triangleright \mathtt{B}.o_j} \Gamma_*, \{k[\mathtt{C}]\colon [\![G']\!]_{\mathtt{C}} \mid k[\mathtt{C}] \in \Gamma_k\}, \{k[\mathtt{C}\rangle\mathtt{D}]\colon [\![G']\!]^{\mathtt{D}}_{\mathtt{C}} \mid k[\mathtt{C}\rangle\mathtt{D}] \in \Gamma_k\}}\ \lfloor^{\Gamma}|_{\mathsf{Send}}\rceil$$

Hence (T|4.3) holds and $\Gamma' = \Gamma_*, \{k[\mathtt{C}]\colon [\![G']\!]_{\mathtt{C}} \mid k[\mathtt{C}] \in \Gamma_k\}, \{k[\mathtt{C}\rangle\mathtt{D}]\colon [\![G']\!]^{\mathtt{D}}_{\mathtt{C}} \mid k[\mathtt{C}\rangle\mathtt{D}] \in \Gamma_k\}$. We now prove (T|4.4) by proving that rule $\lfloor^{\mathsf{T}}|_{\mathsf{DC}}\rceil$ applies to $\Gamma' \vdash D', C'$.

$$\frac{\mathbf{pco}(\Gamma') \qquad \Gamma' \vdash C' \qquad \Gamma' \vdash D'}{\Gamma' \vdash D', C'}\ \lfloor^{\mathsf{T}}|_{\mathsf{DC}}\rceil$$

Hence we need to prove ① $\mathbf{pco}(\Gamma')$, ② $\Gamma' \vdash C'$, and ③ $\Gamma' \vdash D'$

Proof of ① For all sessions $k' \in \Gamma_*$, $\mathbf{pco}(\Gamma')$ holds as $\mathbf{pco}(\Gamma)$ holds by (T|4.1). For session k, $\mathbf{pco}(\Gamma')$ holds by construction.

□

Proof of ② From the derivation on $\Gamma \vdash D, k : \mathtt{p}[\mathtt{A}].e \multimap \mathtt{B}.o_j; C$ we know that $\Gamma_1, k[\mathtt{A}] : \llbracket G_j \rrbracket_{\mathtt{A}} \vdash C$. Let $\Gamma'' = \Gamma_1, k[\mathtt{A}] : \llbracket G_j \rrbracket_{\mathtt{A}}$ and $\Gamma'_k = \Gamma_1 \setminus \Gamma_* = \Gamma_k \setminus \{k[\mathtt{A}] : \llbracket G \rrbracket_{\mathtt{A}}\}$. We can write $\Gamma'' = \Gamma_*, \Gamma'_k, k[\mathtt{A}] : \llbracket G_j \rrbracket_{\mathtt{A}}$. Note that in the premise of rule $\lfloor^{\mathsf{T}}|_{\mathsf{Send}}\rceil$ that types the continuation C, the buffer types in Γ (i.e., those in Γ_1) are unaffected. Therefore $\Gamma''(k[\mathtt{A}\rangle\mathtt{B}]) \neq \Gamma'(k[\mathtt{A}\rangle\mathtt{B}])$, however from Lemma 2 we know that we can omit to consider buffer types as they are irrelevant for the typing of choreographies. For all sessions $k' \neq k$ in Γ'' their local typings are the same in Γ'. For session k, the typing $\Gamma''(k[\mathtt{A}]) = \Gamma'(k[\mathtt{A}]) = \llbracket G_j \rrbracket_{\mathtt{A}}$. From Lemma 3, for all other $k[\mathtt{C}] \in \Gamma'', \mathtt{C} \neq \mathtt{A}$ it holds that $\Gamma''(k[\mathtt{C}]) = \llbracket G \rrbracket_{\mathtt{C}}$, $\Gamma'(k[\mathtt{C}]) = \llbracket G' \rrbracket_{\mathtt{C}}$, and $\llbracket G' \rrbracket_{\mathtt{C}} \prec \llbracket G \rrbracket_{\mathtt{C}}$. Therefore $\Gamma' \prec \Gamma''$ and ② holds by Lemma 1.

□

Proof of ③ To prove $\Gamma' \vdash D'$ we need to prove that the conditions of Definition 2 hold. (D|2.1) holds by the application of rule $\lfloor^{\mathsf{D}}|_{\mathsf{Send}}\rceil$, by construction of Γ', and by (T|4.1). (D|2.2) holds for all sessions $k' \neq k$ by application of rule $\lfloor^{\mathsf{D}}|_{\mathsf{Send}}\rceil$ and the construction of Γ'. The same holds true for session k and any process $\mathtt{q} : k[\mathtt{C}] \in \Gamma' \mid \mathtt{C} \neq \mathtt{B}$.

Finally, we need to prove that $\Gamma'(\ k[\mathtt{A}\rangle\mathtt{B}]\ ) = \mathtt{bte}(\ \mathtt{A}, D'(k[\mathtt{A}\rangle\mathtt{B}])\ )$. From (T|4.1) we know that *i*) $\Gamma(k[\mathtt{A}\rangle\mathtt{B}]) = T$ and *ii*) let $D(k[\mathtt{A}\rangle\mathtt{B}]) = \tilde{m}$, that $\mathtt{bte}(\mathtt{A}, \tilde{m}) = T$. From Definition 3 we have a direct proof that $\mathtt{bte}(\ \mathtt{A}, m_1 :: \cdots :: m_n\ ) = \mathtt{bte}(\mathtt{A}, m_1)\ ;\ \ldots\ ;\ \mathtt{bte}(\mathtt{A}, m_n)$.

Now, from the reduction on $\lfloor^{\mathsf{C}}|_{\mathsf{Send}}\rceil$ we know that

$$D'(\ k[\mathtt{A}\rangle\mathtt{B}]\ ) = m' = \tilde{m} :: (o_j, v)$$

And therefore, $\mathtt{bte}(\mathtt{A}, m') = T;\ \mathtt{bte}(\mathtt{A},\ (o_j, v))$. From the reductions on Γ and G, we observe that the reduction on G do not affect the context $\mathcal{G}$ (which contains local type T), thus, by the rules of the definition of the Buffer Type Projection (Figure 7), we have

$$\llbracket G' \rrbracket^{\mathtt{A}}_{\mathtt{B}} = T; \&\mathtt{A}.o_j(U_j)$$

Hence, from the reduction on rule $\lfloor^{\Gamma}|_{\mathsf{Send}}\rceil$, we know that $\Gamma'(\ k[\mathtt{B}\rangle\mathtt{A}]\ ) = \Gamma'(\ \llbracket G' \rrbracket^{\mathtt{A}}_{\mathtt{B}}\ ) = T; \&\mathtt{A}.o_j(U_j)$. Finally, from the typing rule $\lfloor^{\mathsf{T}}|_{\mathsf{Send}}\rceil$ we know that $\mathtt{p}.e \vdash U_j$ and from reduction rule $\lfloor^{\mathsf{C}}|_{\mathsf{Send}}\rceil$ that $v = \mathbf{eval}(\ e, D(\mathtt{p})\ )$, thus v has type U_j. Hence, $\mathtt{bte}(\ \mathtt{A}, (o_j, v)\ ) = \&\mathtt{A}.o_j(U_j)$ and

$$\Gamma'(\ k[\mathtt{A}\rangle\mathtt{B}]\ ) = T; \&\mathtt{A}.o_j(U_j) = \mathtt{bte}(\ \mathtt{A}, D'(k[\mathtt{A}\rangle\mathtt{B}])\ )$$

□

Case $\lfloor^{\mathsf{C}}|_{\mathsf{Recv}}\rceil$
The case is:

$$\frac{j \in I \quad D, k : \mathtt{A} \multimap \mathtt{q}[\mathtt{B}].o_j(x_j) \blacktriangleright D'}{D,\ k : \mathtt{A} \multimap \mathtt{q}[\mathtt{B}].\{o_i(x_i); C_i\}_{i \in I} \xrightarrow{k:\mathtt{A}\rangle\mathtt{B}.o_j(x_j)} D',\ C_j}\ \lfloor^{\mathsf{C}}|_{\mathsf{Recv}}\rceil$$

(T|4.2) has reductum $C' = C_j$. Since we could apply $\lfloor^{\mathsf{C}}|_{\mathsf{Recv}}\rceil$, we know that $D(k[\mathtt{A}\rangle\mathtt{B}]) = (o_j, v) :: \tilde{m}$. Let $D_1 = D\big[\mathtt{q} \mapsto D(\mathtt{q})[x \mapsto v]\big]$, from the application of rule $\lfloor^{\mathsf{D}}|_{\mathsf{Recv}}\rceil$, we know that $D' = D_1\big[k[\mathtt{A}\rangle\mathtt{B}] \mapsto \tilde{m}\big]$. To prove (T|4.3) we must prove that rule $\lfloor^{\Gamma}|_{\mathsf{Recv}}\rceil$ is applicable.

Since (T|4.1) holds $\mathbf{pco}(\Gamma)$ and $\Gamma \vdash D$ hold and therefore we know that, by (D|2.2), $\Gamma(k[\mathtt{A}\rangle\mathtt{B}]) = \mathtt{bte}(\mathtt{A},\ (o_j, v) ::\ \tilde{m})$.

Let $\vdash v : U_j$, then $\mathtt{bte}(\mathtt{A},\ (o_j, v) ::\ \tilde{m}) = \&\mathtt{A}.o_j(U_j); T$ where $T = \mathtt{bte}(\mathtt{A},\ \tilde{m})$ by Definition 3 and $\Gamma(k[\mathtt{A}\rangle\mathtt{B}]) = \&\mathtt{A}.o_j(U_j); T$. Since $\mathbf{pco}(\Gamma)$ holds, there exists a global type G for session k such

that $G = \mathcal{G}[\mathtt{A}\rangle\mathtt{B}.o_j(U_j);G_j]$. Let π be the reduction of G with rules $\lfloor^{\mathsf{G}}|_{\mathsf{Eq}}\rceil$ and $\lfloor^{\mathsf{G}}|_{\mathsf{Recv}}\rceil$, we observe the following derivation:

$$\pi = \left\{ \begin{array}{c|l} G \simeq_{\mathsf{G}} G_1 \quad \dfrac{}{G_1 \xrightarrow{\gamma} G'}\ \lfloor^{\mathsf{G}}|_{\mathsf{Recv}}\rceil \quad G \simeq_{\mathsf{G}} G' & G_1 = \mathtt{A}\rangle\mathtt{B}.o_j(U_j);\mathcal{G}[G_j] \\ \vdots\ \lfloor^{\mathsf{G}}|_{\mathsf{Eq}}\rceil & G' = \mathcal{G}[G_j] \\ G \xrightarrow{\gamma} G' & \gamma = \mathtt{A}\rangle\mathtt{B}.o_j(U_j) \end{array} \right.$$

In the reductions, since C,D reduces with $\beta = k : \mathtt{A}\rangle\mathtt{B}.o_j(x_j)$ and G types C,D in Γ, there are no other exchanges from A to B in G that could prevent from obtaining, after a finite number of derivations on rule $\lfloor^{\mathsf{G}}|_{\mathsf{Eq}}\rceil$, the swap-equivalence $G \simeq_{\mathsf{G}} G_1$. Then, applying rule $\lfloor^{\mathsf{G}}|_{\mathsf{Send}}\rceil$, G_1 can reduce to G'.

Given π, we can use it to write the reduction at the level the typing environment Γ, applying rule $\lfloor^{\Gamma}|_{\mathsf{Recv}}\rceil$. Below, we consider $\Gamma = \Gamma_*,\Gamma_k$ where Γ_k contains all and only typings of session k in Γ.

$$\dfrac{k \notin \Gamma_* \quad \Gamma_k \subseteq [\![G]\!]_k \quad \{k[\mathtt{A}]: T, k[\mathtt{B}]: T'\} \in \Gamma_k \quad \Gamma_* \vdash \mathtt{q}: k[\mathtt{B}] \quad \overset{\pi}{\vdots}\ G \xrightarrow{\mathtt{A}\rangle\mathtt{B}.o_j} G'}{\Gamma_*,\Gamma_k \xrightarrow{k:\mathtt{A}\rangle\mathtt{B}.o_j(x)} \Gamma_*, \{k[\mathtt{C}]: [\![G']\!]_{\mathtt{C}} \mid k[\mathtt{C}] \in \Gamma_k\}, \{k[\mathtt{C}\rangle\mathtt{D}]: [\![G']\!]^{\mathtt{D}}_{\mathtt{C}} \mid k[\mathtt{C}\rangle\mathtt{D}] \in \Gamma_k\}, \mathtt{q}.x: U_j}\ \lfloor^{\Gamma}|_{\mathsf{Recv}}\rceil$$

Hence (T|4.3) holds and $\Gamma' = \Gamma_*, \{[\![G']\!]_{\mathtt{C}} \mid k[\mathtt{C}] \in \Gamma_k\}, \mathtt{q}.x: U_j$.

(T|4.4) holds if we can apply rule $\lfloor^{\mathsf{T}}|_{\mathsf{DC}}\rceil$ on $\Gamma' \vdash D', C'$

$$\dfrac{\mathbf{pco}(\Gamma') \quad \Gamma' \vdash C' \quad \Gamma' \vdash D'}{\Gamma' \vdash D', C'}\ \lfloor^{\mathsf{T}}|_{\mathsf{DC}}\rceil$$

and we need to prove ① $\mathbf{pco}(\Gamma')$, ② $\Gamma' \vdash C'$, and ③ $\Gamma' \vdash D'$

The proof of ① for this case is similar to that of ① for case $\lfloor^{\mathsf{C}}|_{\mathsf{Send}}\rceil$.

Proof of ② From (T|4.1), partitioning $\Gamma = \Gamma_1, k[\mathtt{B}] : \&\mathtt{A}.o_j(U_j); [\![G_j]\!]_{\mathtt{B}}$ and since $j \in I$ from rule $\lfloor^{\mathsf{C}}|_{\mathsf{Recv}}\rceil$, we can write the derivation

$$\dfrac{\mathbf{pco}(\Gamma) \quad \Gamma \vdash D \quad \dfrac{j \in I \quad \Gamma_1 \vdash \mathtt{q}: k[\mathtt{B}] \quad \Gamma_1, \mathtt{q}.x_j: U_j, k[\mathtt{B}]: [\![G_j]\!]_{\mathtt{B}} \vdash C_j}{\Gamma_1, k[\mathtt{B}]: \&\mathtt{A}.o_j(U_j); [\![G_j]\!]_{\mathtt{B}} \vdash k: \mathtt{A} \multimap \mathtt{q}[\mathtt{B}].\{o_i(x_i);C_i\}_{i\in I}}\ \lfloor^{\mathsf{T}}|_{\mathsf{Recv}}\rceil}{\Gamma \vdash D, k: \mathtt{A} \multimap \mathtt{q}[\mathtt{B}].\{o_i(x_i);C_i\}_{i\in I}}\ \lfloor^{\mathsf{T}}|_{\mathsf{DC}}\rceil$$

hence we know that $\Gamma_1, \mathtt{q}.x_j: U_j, k[\mathtt{B}]: [\![G_j]\!]_{\mathtt{B}} \vdash C_j$.

Let $\Gamma'' = \Gamma_1, \mathtt{q}.x_j: U_j, k[\mathtt{B}]: [\![G_j]\!]_{\mathtt{B}} \vdash C_j$ and $\Gamma'_k = \Gamma_1 \setminus \Gamma_* = \Gamma_k \setminus \{k[\mathtt{B}]: [\![G]\!]_{\mathtt{B}}\}$. We can write $\Gamma'' = \Gamma_*, \Gamma'_k, k[\mathtt{B}]: [\![G_j]\!]_{\mathtt{B}}$. Similarly to ② for case $\lfloor^{\mathsf{C}}|_{\mathsf{Send}}\rceil$, $\Gamma''(k[\mathtt{A}\rangle\mathtt{B}]) \neq \Gamma'(k[\mathtt{A}\rangle\mathtt{B}])$, but we omit to consider buffer types as they are irrelevant for the typing of choreographies by Lemma 2. For all sessions in Γ'', their local typings are the same as in Γ'. We consider in particular k on which we applied the reduction for this case for which it holds

$$\forall\, k[\mathtt{C}] \in \Gamma'',\ \Gamma''(k[\mathtt{C}]) = \Gamma'(k[\mathtt{C}]) = [\![G']\!]_{\mathtt{C}}$$

□

Proof of ③ To prove $\Gamma' \vdash D'$ we prove the conditions in Definition 2. (D|2.1) holds from the application of rule ⌊D|Recv⌉, (T|4.1), and the construction of Γ'. (D|2.2) holds for all $\mathtt{p}.x$ from the application of rule ⌊D|Recv⌉, (T|4.1), and the construction of Γ', except for $\mathtt{q}.x_j$ which is not defined in Γ. However the condition holds by construction of $\Gamma' = \Gamma_1, \mathtt{q}.x_j : U_j, k[\mathtt{B}] : \llbracket G_j \rrbracket_{\mathtt{B}}$. (D|2.2) holds for all sessions $k' \neq k$ by the application of rule ⌊D|Recv⌉ and the construction of Γ'. The same holds true for session k and any process $\mathtt{p} : k[\mathtt{C}] \in \Gamma \mid \mathtt{C} \neq \mathtt{B}$.

For $\mathtt{q} : k[\mathtt{B}]$ and role $\mathtt{A}$ we know from the application of ⌊C|Send⌉ that $D'(k[\mathtt{A}\rangle\mathtt{B}]) = \tilde{m}$. Since we took G such that $\llbracket G \rrbracket_{\mathtt{B}}^{\mathtt{A}} = \&\mathtt{A}.o_j(U_j); T$, where $T = \mathtt{bte}(\mathtt{A}, \tilde{m})$, then $\llbracket G' \rrbracket_{\mathtt{B}}^{\mathtt{A}} = T$.

☐

Case ⌊C|Start⌉

The case is:

$$\frac{D\#k', \tilde{\mathtt{r}} \quad \delta = \mathtt{start}\, k' :\ l.\mathtt{p}[\mathtt{A}], \widetilde{l.\mathtt{r}[\mathtt{B}]} \quad D, \delta \blacktriangleright D'}{D,\ \mathtt{start}\, k : \mathtt{p}[\mathtt{A}] \twoheadleftarrow\!\!\!\twoheadrightarrow \widetilde{l.\mathtt{q}[\mathtt{B}]}; C \quad \xrightarrow{\tau} \quad D',\ C[k'/k][\tilde{\mathtt{r}}/\tilde{\mathtt{q}}]} \ \lfloor \mathsf{C}|\mathsf{Start} \rceil$$

Where (T|4.5) has $C' = C[k'/k][\tilde{\mathtt{r}}/\tilde{\mathtt{q}}]$. D' is defined non-deterministically but abides the requirements defined in rule ⌊D|Start⌉. Let $\widetilde{\mathtt{s}[\mathtt{C}]} = \mathtt{p}[\mathtt{A}], \widetilde{\mathtt{r}[\mathtt{B}]}$. Since (T|4.1) holds, we can apply rule ⌊T|Start⌉. We partition $\Gamma = \Gamma_1, \tilde{l} : G\langle \mathtt{A}|\tilde{\mathtt{B}}|\tilde{\mathtt{B}}\rangle$

$$\frac{\Gamma_1, \tilde{l} : G\langle \mathtt{A}|\tilde{\mathtt{B}}|\tilde{\mathtt{B}}\rangle, \mathbf{init}(\ \widetilde{\mathtt{s}'[\mathtt{C}]}\ , k,\ G\ ) \vdash C \quad \widetilde{\mathtt{s}'[\mathtt{C}]} = \mathtt{p}[\mathtt{A}], \widetilde{\mathtt{q}[\mathtt{B}]} \quad \tilde{\mathtt{q}} \notin \Gamma_1}{\Gamma_1, \tilde{l} : G\langle \mathtt{A}|\tilde{\mathtt{B}}|\tilde{\mathtt{B}}\rangle \vdash \mathtt{start}\, k : \mathtt{p}[\mathtt{A}] \twoheadleftarrow\!\!\!\twoheadrightarrow \widetilde{l.\mathtt{q}[\mathtt{B}]}; C} \ \lfloor \mathsf{T}|\mathsf{Start} \rceil$$

Coherently with the semantics of rule ⌊C|Start⌉, we take $\Gamma' = \Gamma, \mathbf{init}(\ \widetilde{\mathtt{s}[\mathtt{C}]},\ k',\ G\ )$—obtainable from the typing environment in the left-most premise of rule ⌊T|Start⌉, α-renaming: *i)* typings on session k to session k' and *ii)* process identifies $\tilde{\mathtt{q}}$ to $\tilde{\mathtt{r}}$ in $\widetilde{\mathtt{s}'[\mathtt{C}]}$ (i.e., such that $\widetilde{\mathtt{s}[\mathtt{C}]} = [\ \widetilde{\mathtt{r}[\mathtt{C}]}\ /\ \widetilde{\mathtt{q}[\mathtt{C}]}\ ]\ \widetilde{\mathtt{s}'[\mathtt{C}]}$)—and we prove the case by proving that we can apply rule ⌊T|DC⌉ on $\Gamma' \vdash D', C'$, i.e, that the following hold: ① $\mathbf{pco}(\Gamma')$, ② $\Gamma' \vdash C'$, and ③ $\Gamma' \vdash D'$.

Proof of ① ① holds for all session $k'' \in \Gamma', k'' \neq k'$ by (T|4.1). For session k' ① holds by construction. ☐

Proof of ② By (T|4.1) we could apply ⌊T|Start⌉ where $\Gamma, \mathbf{init}(\ \widetilde{\mathtt{s}[\mathtt{C}]},\ k,\ G\ ) \vdash C$. Since Γ' is obtained by α-renaming of the left-most premise of Rule ⌊T|Start⌉, which types the continuation C, Γ' types $C[k'/k][\tilde{\mathtt{r}}/\tilde{\mathtt{q}}]$ and ② holds by construction. ☐

Proof of ③ To prove ③ we prove the conditions in Definition 2. (D|2.1–D|2.2) hold by the application of rule ⌊D|Start⌉ and the construction of Γ'. ☐

Case ⌊C|PStart⌉

The case is:

$$\frac{\begin{array}{c} i \in \{1, \ldots, n\} \qquad D\#k', \tilde{\mathtt{r}} \qquad \{\ \widetilde{l.\mathtt{B}}\ \} = \uplus_i \{\ \widetilde{l_i.\mathtt{B}_i}\ \} \qquad \{\tilde{\mathtt{r}}\} = \bigcup_i \{\tilde{\mathtt{r}}_i\} \\ \mathtt{p} \in D(l) \qquad \delta = \mathtt{start}\, k' :\ l.\mathtt{p}[\mathtt{A}], \widetilde{l_1.\mathtt{r}_1[\mathtt{B}_1]}, \ldots, \widetilde{l_n.\mathtt{r}_n[\mathtt{B}_n]} \qquad D, \delta \blacktriangleright D' \end{array}}{\begin{array}{l} D, \mathtt{req}\, k : \mathtt{p}[\mathtt{A}] \twoheadleftarrow\!\!\!\twoheadrightarrow \widetilde{l.\mathtt{B}}; C \mid \prod_i \left(\mathtt{acc}\, k : \widetilde{l_i.\mathtt{q}_i[\mathtt{B}_i]}; C_i\right) \xrightarrow{\tau} \\ \qquad D',\ C[k'/k] \mid \prod_i \left(\ C_i[k'/k][\tilde{\mathtt{r}}_i/\tilde{\mathtt{q}}_i]\ \right) \mid \prod_i \left(\mathtt{acc}\, k : \widetilde{l_i.\mathtt{q}_i[\mathtt{B}_i]}; C_i\right) \end{array}} \ \lfloor \mathsf{C}|\mathsf{PStart} \rceil$$

Where (T|4.5) has $C' = C[k'/k] \mid \prod_i \left(\ C_i[k'/k][\tilde{\mathtt{r}}_i/\tilde{\mathtt{q}}_i]\ \right) \mid \prod_i \left(\mathtt{acc}\, k : \widetilde{l_i.\mathtt{q}_i[\mathtt{B}_i]}; C_i\right)$. D' is defined non-deterministically but abides the requirements defined in rule ⌊D|Start⌉.

We partition Γ such that:

- $\Gamma = \Gamma_r, \Gamma_a$
- $\Gamma_r \vdash \tilde{l} : G\langle \mathtt{A}|\tilde{\mathtt{B}}|\varnothing\rangle$

- $\Gamma_a \vdash \tilde{l} \colon G\langle \mathtt{A}|\tilde{\mathtt{B}}|\tilde{\mathtt{B}}\rangle$
- $\Gamma_a = \Gamma_1, \tilde{l} \colon G\langle \mathtt{A}|\tilde{\mathtt{B}}|\widetilde{\mathtt{B}_1}\rangle, \cdots, \Gamma_n, \tilde{l} \colon G\langle \mathtt{A}|\tilde{\mathtt{B}}|\widetilde{\mathtt{B}_n}\rangle$
- $\Gamma_a^i = \Gamma_i, \tilde{l} \colon G\langle \mathtt{A}|\tilde{\mathtt{B}}|\widetilde{\mathtt{B}_i}\rangle, \cdots, \Gamma_n, \tilde{l} \colon G\langle \mathtt{A}|\tilde{\mathtt{B}}|\widetilde{\mathtt{B}_n}\rangle$

and we can write the derivation

$$\dfrac{\mathbf{pco}(\Gamma) \qquad \Gamma \vdash D \qquad \dfrac{\dfrac{\Gamma_r, \mathsf{p} \colon k[\mathtt{A}], k[\mathtt{A}] \colon [\![G]\!]_{\mathtt{A}} \vdash C \qquad \Gamma_r \vdash \tilde{l} \colon G\langle \mathtt{A}|\tilde{\mathtt{B}}|\varnothing\rangle}{\Gamma_r \vdash \mathsf{req}\, k : \mathsf{p}[\mathtt{A}] \Leftrightarrow \widetilde{l.\mathtt{B}}; C}\lfloor\mathsf{T}|_{\mathsf{Req}}\rceil \qquad \Delta_1}{\Gamma \vdash \mathsf{req}\, k : \mathsf{p}[\mathtt{A}] \Leftrightarrow \widetilde{l.\mathtt{B}}; C \mid \prod_{i \in I} \left(\mathsf{acc}\, k : \widetilde{l_i.\mathsf{q}_i[\mathtt{B}_i]}; C_i\right)}\lfloor\mathsf{T}|_{\mathsf{Par}}\rceil}{\Gamma \vdash D, \mathsf{req}\, k : \mathsf{p}[\mathtt{A}] \Leftrightarrow \widetilde{l.\mathtt{B}}; C \mid \prod_{i \in I} \left(\mathsf{acc}\, k : \widetilde{l_i.\mathsf{q}_i[\mathtt{B}_i]}; C_i\right)}\lfloor\mathsf{T}|_{\mathsf{DC}}\rceil$$

$$\Delta_i = \left\{ \dfrac{\dfrac{\tilde{l}_i \subseteq \tilde{l} \qquad \Gamma_i, \tilde{l} \colon G\langle \mathtt{A}|\tilde{\mathtt{B}}|\varnothing\rangle, \mathbf{init}(\ \widetilde{\mathsf{q}_i[\mathtt{B}_i]},\ k,\ G\ ) \vdash C_i \qquad \tilde{\mathsf{q}}_i \notin \Gamma}{\Gamma_i, \tilde{l} \colon G\langle \mathtt{A}|\tilde{\mathtt{B}}|\widetilde{\mathtt{B}_i}\rangle \vdash \mathsf{acc}\, k : \widetilde{l_i.\mathsf{q}_i[\mathtt{B}_i]}; C_i}\lfloor\mathsf{T}|_{\mathsf{Acc}}\rceil \qquad \Delta_{i+1}}{\Gamma_a^i \vdash \mathsf{acc}\, k : \widetilde{l_i.\mathsf{q}_i[\mathtt{B}_i]}; C_i \mid \prod_{j \in I \setminus \{1, \cdots, i\}} \left(\mathsf{acc}\, k : \widetilde{l_j.\mathsf{q}_j[\mathtt{B}_j]}; C_j\right)}\lfloor\mathsf{T}|_{\mathsf{Par}}\rceil \right.$$

Let $\widetilde{\mathsf{s}[\mathtt{C}]} = \widetilde{\mathsf{p}[\mathtt{A}]}, \widetilde{\mathsf{r}_1[\mathtt{B}_1]}, \cdots, \widetilde{\mathsf{r}_n[\mathtt{B}_n]}$.
To prove (T|4.6) we take

$$\Gamma' = \Gamma, \mathbf{init}(\ \widetilde{\mathsf{s}[\mathtt{C}]},\ k',\ G\ ) = \Gamma_r, \Gamma_a, \mathbf{init}(\ \widetilde{\mathsf{s}[\mathtt{C}]},\ k',\ G\ )$$

and we partition $\mathbf{init}(\ \widetilde{\mathsf{s}[\mathtt{C}]},\ k',\ G\ )$ such that

$$\Gamma' = \Gamma'_r, \Gamma'_a, \Gamma_a$$

Where

- $\Gamma'_r = \Gamma_r, \mathbf{init}(\ \mathsf{p}[\mathtt{A}],\ k',\ G\ )$
- $\Gamma'_a = \Gamma'_1, \cdots, \Gamma'_n$
- $\Gamma'_i = \Gamma_i, \tilde{l} \colon G\langle \mathtt{A}|\tilde{\mathtt{B}}|\varnothing\rangle, \mathbf{init}(\ \widetilde{\mathsf{r}_i[\mathtt{B}_i]},\ k',\ G\ )$ where $i \in \{1, \ldots, n\}$

To prove (T|4.6) we must prove we can apply rule $\lfloor\mathsf{T}|_{\mathsf{DC}}\rceil$ on $\Gamma' \vdash D', C'$.

$$\dfrac{①\ \mathbf{pco}(\Gamma') \qquad ③\ \Gamma' \vdash D' \qquad ② \left\{ \dfrac{\text{(2a)}\ \Gamma'_r \vdash C[k'/k] \qquad \dfrac{\text{(2b)}\ \Gamma'_a \vdash \prod_i \left(\ C_i[k'/k][\tilde{\mathsf{r}}_i/\tilde{\mathsf{q}}_i]\ \right) \quad \text{(2c)}\ \Gamma_a \vdash \prod_i \left(\mathsf{acc}\, k : \widetilde{l_i.\mathsf{q}_i[\mathtt{B}_i]}; C_i\right)}{\Gamma'_a, \Gamma_a \vdash \prod_i \left(\ C_i[k'/k][\tilde{\mathsf{r}}_i/\tilde{\mathsf{q}}_i]\ \right) \mid \prod_i \left(\mathsf{acc}\, k : \widetilde{l_i.\mathsf{q}_i[\mathtt{B}_i]}; C_i\right)}\lfloor\mathsf{T}|_{\mathsf{Par}}\rceil}{\Gamma' \vdash C[k'/k] \mid \prod_i \left(\ C_i[k'/k][\tilde{\mathsf{r}}_i/\tilde{\mathsf{q}}_i]\ \right) \mid \prod_i \left(\mathsf{acc}\, k : \widetilde{l_i.\mathsf{q}_i[\mathtt{B}_i]}; C_i\right)}\lfloor\mathsf{T}|_{\mathsf{Par}}\rceil \right.}{\Gamma' \vdash D', C'}\lfloor\mathsf{T}|_{\mathsf{DC}}\rceil$$

Proof of ① ① holds by construction. □

Proof of ② ② holds as

- (2a) holds by α-renaming $(\Gamma_r, \mathsf{p} \colon k[\mathtt{A}], k[\mathtt{A}] \colon [\![G]\!]_{\mathtt{A}})[k'/k] \vdash C[k'/k]$ and by omitting to consider buffer types as of Lemma 2;
- similarly to (2a), (2b) holds by α-renaming on the derivation of

$$(\ \Gamma_i, \tilde{l} \colon G\langle \mathtt{A}|\tilde{\mathtt{B}}|\varnothing\rangle, \mathbf{init}(\ \widetilde{\mathsf{q}_i[\mathtt{B}_i]},\ k,\ G\ )\ )[k'/k][\tilde{\mathsf{r}}_i/\tilde{\mathsf{q}}_i] \vdash C_i[k'/k][\tilde{\mathsf{r}}_i/\tilde{\mathsf{q}}_i]$$

  and by Lemma 2;
- (2c) holds by (T|4.1).

□

Proof of ③ The proof of ③ of this case is similar to the of ③ for Case $\lfloor^{\mathsf{C}}|_{\mathsf{Start}}\rceil$. □

Case $\lfloor^{\mathsf{C}}|_{\mathsf{Cond}}\rceil$
The case is:

$$\frac{i = 1 \text{ if } \mathbf{eval}(\, e, D(\mathsf{p})\,) = \mathtt{true}, i = 2 \text{ otherwise}}{D,\ \mathtt{if}\ \mathsf{p}.e\ \{C_1\}\ \mathtt{else}\ \{C_2\} \quad \xrightarrow{\tau} \quad D,\ C_i}\ \lfloor^{\mathsf{C}}|_{\mathsf{Cond}}\rceil$$

In (T|4.5) $D' = D$ and we have two cases for $C' = C_1$ or $C' = C_2$.

From (T|4.1) we can write

$$\frac{\Gamma \vdash \mathsf{p}.e\colon \mathbf{bool} \quad \Gamma \vdash C_1 \quad \Gamma \vdash C_2}{\Gamma \vdash \mathtt{if}\ \mathsf{p}.e\ \{C_1\}\ \mathtt{else}\ \{C_2\}}\ \lfloor^{\mathsf{T}}|_{\mathsf{Cond}}\rceil$$

The proof of (T|4.6) follows directly from the premises of the typing derivation as $\Gamma \vdash D = D'$ and in both cases that $C' = C_1$ or $C' = C_2$ it holds that $\Gamma \vdash C'$ from the premises of $\lfloor^{\mathsf{T}}|_{\mathsf{Cond}}\rceil$.

Case $\lfloor^{\mathsf{C}}|_{\mathsf{Ctx}}\rceil$
The case is:

$$\frac{D, C_1 \xrightarrow{\beta} D', C_1'}{D, \mathtt{def}\ X = C_2\ \mathtt{in}\ C_1 \quad \xrightarrow{\beta} \quad D', \mathtt{def}\ X = C_2\ \mathtt{in}\ C_1'}\ \lfloor^{\mathsf{C}}|_{\mathsf{Ctx}}\rceil$$

From (T|4.1) we know that, $\Gamma = \Gamma_1, X\colon \Gamma_x$

$$\frac{\mathbf{pco}(\Gamma) \quad \dfrac{\Gamma_1, X\colon \Gamma_x \vdash C_1 \quad \Gamma_x, X\colon \Gamma_x \vdash C_2 \quad \Gamma_x|_{\mathbf{locs}} \subseteq \Gamma}{\Gamma \vdash \mathtt{def}\ X = C_2\ \mathtt{in}\ C_1}\ \lfloor^{\mathsf{T}}|_{\mathsf{Def}}\rceil \quad \Gamma \vdash D}{\Gamma \vdash D, \mathtt{def}\ X = C_2\ \mathtt{in}\ C_1}\ \lfloor^{\mathsf{T}}|_{\mathsf{DC}}\rceil$$

The proof is divided in two cases on the type of β.

Case $\beta \neq \tau$
D, C_1 reduces on some session k. By the induction hypothesis since $\Gamma \vdash D, C_1$ we can find Γ' such that (T|4.3) holds. We prove (T|4.4) by proving that we can apply $\lfloor^{\mathsf{T}}|_{\mathsf{DC}}\rceil$ on $\Gamma' \vdash D', \mathtt{def}\ X = C_2\ \mathtt{in}\ C_1'$ and therefore that ① $\mathbf{pco}(\Gamma')$ holds, ② $\Gamma' \vdash \mathtt{def}\ X = C_2\ \mathtt{in}\ C_1$ and ③ $\Gamma' \vdash D'$.

① holds by the construction of Γ' and ③ holds by the induction hypothesis.

To prove ② we have to prove that $\Gamma' \vdash X\colon C_2$ and $\Gamma_x|_{\mathbf{locs}} \subseteq \Gamma'$.

From the induction hypothesis we have that $\Gamma \xrightarrow{\beta} \Gamma'$ and $\Gamma' \vdash D', C_1'$. By construction of Γ' it holds that $\Gamma' = \Gamma_*', \Gamma_k'$ where $\Gamma' \cap \Gamma = \Gamma_*$ such that $k \notin \Gamma_*$ and $\Gamma = \Gamma_*, \Gamma_k$ where $\Gamma_k \subseteq [\![G]\!]_k$ for some G. Therefore it holds that $\Gamma_* \vdash X\colon \Gamma_x$ and thus that $\Gamma' \vdash X\colon \Gamma_x$. The same applies to $\Gamma_x|_{\mathbf{locs}} \subseteq \Gamma_*$ which proves $\Gamma_x|_{\mathbf{locs}} \subseteq \Gamma'$.

Case $\beta = \tau$
from the induction hypothesis, for any considered derivation we have $\Gamma \subseteq \Gamma'$. We prove (T|4.6) by proving that we can apply $\lfloor^{\mathsf{T}}|_{\mathsf{DC}}\rceil$ on $\Gamma' \vdash D', \mathtt{def}\ X = C_2\ \mathtt{in}\ C_1'$. ①, ②, and ③ hold by construction of Γ'.

Case $\lfloor^{\mathsf{C}}|_{\mathsf{Par}}\rceil$
The case is:

$$\frac{D, C_1 \quad \xrightarrow{\beta} \quad D', C_1'}{D, C_1 \mid C_2 \quad \xrightarrow{\beta} \quad D', C_1' \mid C_2}\ \lfloor^{\mathsf{C}}|_{\mathsf{Par}}\rceil$$

From (T|4.1) we have the derivation below, with Γ partitioned as $\Gamma = \Gamma_1, \Gamma_2$

$$\dfrac{\mathbf{pco}(\Gamma) \quad \dfrac{\Gamma_1 \vdash C_1 \quad \Gamma_2 \vdash C_2}{\Gamma \vdash C_1 \mid C_2} \lfloor^{\mathsf{T}}|_{\mathsf{Par}}\rceil \quad \Gamma \vdash D}{\Gamma \vdash D, C_1 \mid C_2} \lfloor^{\mathsf{T}}|_{\mathsf{DC}}\rceil$$

The proof is divided in two cases on the type of β.

Case $\beta \neq \tau$
D, C_1 reduces on some session k. By the induction hypothesis and since $\Gamma_1 \vdash D, C_1$ we can find Γ_1' such that $\Gamma_1 \xrightarrow{\beta} \Gamma_1'$ and $\Gamma_1' \vdash D', C_1'$. Then we take $\Gamma' = \Gamma_1', \Gamma_2$ which proves (T|4.3) to hold. We prove (T|4.4) by proving that we can apply $\lfloor^{\mathsf{T}}|_{\mathsf{DC}}\rceil$ on $\Gamma' \vdash D', C_1' \mid C_2$ and therefore that ① $\mathbf{pco}(\Gamma')$, ② $\Gamma' \vdash C_1' \mid C_2$ and ③ $\Gamma' \vdash D'$ hold. ①, ②, and ③ hold by construction and the induction hypothesis.

Case $\beta = \tau$
from the induction hypothesis, for any derivation we have that $\Gamma_1' \vdash D', C_1'$ and $\Gamma_1 \subseteq \Gamma_1'$. Also in this case we take $\Gamma' = \Gamma_1', \Gamma_2$ and prove (T|4.6) by proving that we can apply $\lfloor^{\mathsf{T}}|_{\mathsf{DC}}\rceil$ on $\Gamma' \vdash D', C_1' \mid C_2$. ①, ②, and ③ hold by construction of Γ' and the induction hypothesis.

Case $\lfloor^{\mathsf{C}}|_{\mathsf{Eq}}\rceil$
The case is:

$$\dfrac{\mathcal{R} \in \{\equiv_{\mathsf{C}}, \simeq_{\mathsf{C}}\} \quad C \,\mathcal{R}\, C_1 \quad D, C_1 \xrightarrow{\beta} D', C_1' \quad C_1' \,\mathcal{R}\, C'}{D, C \quad \xrightarrow{\beta} \quad D', C'} \lfloor^{\mathsf{C}}|_{\mathsf{Eq}}\rceil$$

The proof is divided into two subcases on the type of $\mathcal{R}$.

Case $\mathcal{R} = \equiv_{\mathsf{C}}$
The case is proved by induction hypothesis and Lemma 5.

Case $\mathcal{R} = \simeq_{\mathsf{C}}$
The case is proved by induction hypothesis and Lemma 6.

□

The proof of Theorem 2 follows directly from the proof of Theorem 4 and Lemma 4.

3.2 Proof of Deadlock Freedom

We report below the statement of Theorem 3 enriched with pointers for clearer referencing in the proof.

Theorem 3 (Deadlock-freedom)
(D3.1) $\Gamma \vdash D, C$ and (D3.2) $\mathbf{co}(\Gamma)$ imply that either (D3.3) $C \equiv_{\mathsf{C}} \mathbf{0}$ or (D3.4) there exist D' and C' such that $D, C \to D', C'$.

Like in Carbone and Montesi (2013); Montesi and Yoshida (2013), frontend choreographies enjoy deadlock freedom, provided that they *i*) do not contain free variable names and *ii*) are *well-sorted*, i.e., have no undefined procedure calls. Notably, well-sortedness is guaranteed by the type system.

Proof Proof by induction on the structure of C.

Case $C \equiv_{\mathsf{C}} \mathbf{0}$
trivial.

Case $C = k\colon \mathtt{p}[\mathtt{A}].e \longrightarrow \mathtt{B}.o; C_1$
from (D3.1) and (D3.2) we know that the requirements of $\lfloor^{\mathsf{D}}|_{\mathsf{Send}}\rceil$ hold and we can find D' such that $D, k\colon \mathtt{p}[\mathtt{A}].e \longrightarrow \mathtt{B}.o \blacktriangleright D'$. We can apply Rule $\lfloor^{\mathsf{C}}|_{\mathsf{Send}}\rceil$ for which $C' = C_1$.

Case $C = k\colon \mathtt{p}[\mathtt{A}].e \longrightarrow \mathtt{q}[\mathtt{B}].o(x);C_1$
since (D3.1) holds both receiver and sender are typed by Γ. We apply rule $\lfloor^{\mathsf{C}}|_{\mathsf{Eq}}\rceil$ to split the complete term into respectively a send and a receive partial terms, and similarly to the previous case, we apply rule $\lfloor^{\mathsf{C}}|_{\mathsf{Send}}\rceil$, for which $C' = k\colon \mathtt{A} \longrightarrow \mathtt{q}[\mathtt{B}].o(x);C_1$.

Case $C = k\colon \mathtt{A} \longrightarrow \mathtt{q}[\mathtt{B}].\{o_i(x_i);C_i\}_{i\in I}$
from (D3.1) and (D3.2) we know that the requirements of Rule $\lfloor^{\mathsf{D}}|_{\mathsf{Recv}}\rceil$ hold and $D(k[\mathtt{A}\rangle\mathtt{B}]) = (o_j,t_m) :: \tilde{m}$ for some $j \in I$. We can find D' such that $D,k\colon \mathtt{A} \longrightarrow \mathtt{q}[\mathtt{B}].o_j(x_j) \blacktriangleright D'$ and apply Rule $\lfloor^{\mathsf{C}}|_{\mathsf{Recv}}\rceil$ for which $C' = C_j$.

Case $C = \mathtt{start}\ k : \mathtt{p}[\mathtt{A}] \Leftrightarrow \widetilde{l.\mathtt{q}[\mathtt{B}]};C_1$
from (D3.1) and (D3.2) $\lfloor^{\mathsf{D}}|_{\mathsf{Start}}\rceil$ applies and we can find D' such that $D,\mathtt{start}\ k' : l.\mathtt{p}[\mathtt{A}],\widetilde{l.\mathtt{r}[\mathtt{B}]} \blacktriangleright D'$ for some k', $\tilde{\mathtt{r}}$ fresh. We can apply Rule $\lfloor^{\mathsf{C}}|_{\mathsf{Start}}\rceil$ for which $C' = C_1[k'/k][\tilde{\mathtt{r}}/\tilde{\mathtt{q}}]$.

Case $C = \mathtt{req}\ k : \mathtt{p}[\mathtt{A}] \Leftrightarrow \widetilde{l.\mathtt{B}};C \mid \prod_{i=1}^{n} \left(\mathtt{acc}\ k : \widetilde{l_i.\mathtt{q}_i[\mathtt{B}_i]};C_i\right)$
similarly to the previous case, the requirements of $\lfloor^{\mathsf{D}}|_{\mathsf{Start}}\rceil$ hold and we can find D' such that $D,\mathtt{start}\ k' :\ l.\mathtt{p}[\mathtt{A}],\widetilde{l_1.\mathtt{r}_1[\mathtt{B}_1]},\ldots,\widetilde{l_n.\mathtt{r}_n[\mathtt{B}_n]} \blacktriangleright D'$ for some k' and $\tilde{\mathtt{r}}_1,\cdots,\tilde{\mathtt{r}}_n$ fresh. We can apply Rule $\lfloor^{\mathsf{C}}|_{\mathsf{PStart}}\rceil$ for which
$C' = C[k'/k] \mid \prod_{i=1}^{n} C_i[k'/k][\tilde{\mathtt{r}}_1/\tilde{\mathtt{q}}_1] \mid \prod_{i=1}^{n} \left(\mathtt{acc}\ k : \widetilde{l_i.\mathtt{q}_i[\mathtt{B}_i]};C_i\right)$.

Case $C = C_1 \mid C_2$
we can apply the induction hypothesis and Rule $\lfloor^{\mathsf{C}}|_{\mathsf{Par}}\rceil$ such that $D,C_1 \to D_1,C_1'$ and in (D3.4) $D' = D_1$ and $C' = C_1' \mid C_2$.

Case $C = \mathtt{def}\ X = C_2\ \mathtt{in}\ C_1$
applies the induction hypothesis and Rule $\lfloor^{\mathsf{C}}|_{\mathsf{Ctx}}\rceil$ for which $D,C_1 \to D',C_1'$, where $C' = \mathtt{def}\ X = C_2\ \mathtt{in}\ C_1'$.

Case $\mathtt{def}\ X = C_2\ \mathtt{in}\ X;C_1$
applies Rule $\lfloor^{\mathsf{C}}|_{\mathsf{Eq}}\rceil$ for $\mathtt{def}\ X = C_2\ \mathtt{in}\ X;C_1 \equiv_{\mathsf{C}} \mathtt{def}\ X = C_2\ \mathtt{in}\ C_2;C_1$ and by the induction hypothesis $D,C_2 \to D',C_2'$ and $C' = \mathtt{def}\ X = C_2\ \mathtt{in}\ C_2';C_1$.

Case $C = \mathtt{if}\ \mathtt{p}.e\ \{C_1\}\ \mathtt{else}\ \{C_2\}$
from (D3.1) we know that $\Gamma \vdash \mathtt{p}.e\colon \mathbf{bool}$ and therefore we can apply Rule $\lfloor^{\mathsf{C}}|_{\mathsf{Cond}}\rceil$ and, according to the evaluation of e, we have $C' = C_1$ or $C' = C_2$.

□

3.3 Proof of Encoding Frontend Choreographies to Backend Choreographies

We report below the definition of Theorem 1 of the article for easier referencing.
Theorem 1 *Operational Correspondence (FC $\leftrightarrow$ BC)*
Let $\Gamma \vdash D,C$. Then:

1. (Completeness) $D,C \to D',C'$ implies $\langle\!\langle D \rangle\!\rangle^{\Gamma},C \to \langle\!\langle D' \rangle\!\rangle^{\Gamma'},C'$ for some Γ' s.t. $\Gamma' \vdash D',C'$;
2. (Soundness) $\langle\!\langle D \rangle\!\rangle^{\Gamma},C \to \mathbb{D},C'$ implies $D,C \to D',C'$ and $\mathbb{D} = \langle\!\langle D' \rangle\!\rangle^{\Gamma'}$ for some Γ' s.t. $\Gamma' \vdash D',C'$.

We now proceed to prove the separate results of *(Completeness)* and *(Soundness)*.

Proof (Completeness)

Proof by induction on the derivation of $D,C \to D',C'$.

Case $\lfloor^{\mathsf{C}}|_{\mathsf{Send}}\rceil$
we know that $C = k\colon \mathtt{p}[\mathtt{A}].e \longrightarrow \mathtt{B}.o;C_c$ and we can write the derivation

$$\dfrac{\eta = k\colon \mathtt{p}[\mathtt{A}].e \longrightarrow \mathtt{B}.o \qquad \dfrac{v = \mathbf{eval}(e,D(\mathtt{p})) \quad D(k[\mathtt{A}\rangle\mathtt{B}]) = \tilde{m}}{D,\eta \blacktriangleright D\big[k[\mathtt{A}\rangle\mathtt{B}] \mapsto \tilde{m} :: (o,v)\big]}\lfloor^{\mathsf{D}}|_{\mathsf{Send}}\rceil}{D,\ \eta;C \quad \to D\big[k[\mathtt{A}\rangle\mathtt{B}] \mapsto \tilde{m} :: (o,v)\big],\ C_c}\lfloor^{\mathsf{C}}|_{\mathsf{Send}}\rceil$$

where $C' = C_c$ and $D' = D\big[k[\mathtt{A}\rangle\mathtt{B}] \mapsto \tilde{m} :: (o,v)\big]$. Then, given $\mathbb{D} = \langle\!\langle D \rangle\!\rangle^{\Gamma}$, we can write the derivation

$$\dfrac{\eta = k\colon \mathtt{p}[\mathtt{A}].e \longrightarrow \mathtt{B}.o \qquad \dfrac{l = \underline{k.B.l}(\ \mathbb{D}(\mathtt{p})\ ) \qquad t_c = \underline{k.A.B}(\ \mathbb{D}(\mathtt{p})\ ) \quad t_m = \mathbf{eval}(e, \mathbb{D}(\mathtt{p}))}{\mathbb{D}, k\colon \mathtt{p}[\mathtt{A}].e \longrightarrow \mathtt{B}.o \blacktriangleright \mathbb{D}[\ l : t_c \mapsto \mathbb{D}(l : t_c) :: (o, t_m)\ ]}\ \lfloor^{\mathbb{D}}|_{\mathsf{Send}}\rceil}{\mathbb{D},\ \eta; C \quad \to \mathbb{D}[\ l : t_c \mapsto \mathbb{D}(l : t_c) :: (o, t_m)\ ],\ C_c}\ \lfloor^{\mathsf{C}}|_{\mathsf{Send}}\rceil$$

where the continuation $C' = C_c$ and $\mathbb{D}' = \mathbb{D}[\ l : t_c \mapsto \mathbb{D}(l : t_c) :: (o, t_m)\ ]$. In $\mathbb{D}$, the path $\underline{k.B.l}$ applied on the state of p corresponds to the location l of the process that plays B in session k in Γ, e.g., if $\{\mathtt{q}\colon k[\mathtt{B}], \mathtt{q}@l_{\mathtt{q}}\} \subseteq \Gamma$ then $\underline{k.B.l}(\mathbb{D}(\mathtt{p})) = l_{\mathtt{q}}$. In the reduction above, t_c is the correlation key in $\mathbb{D}$ for process p playing A in k to send messages to the process playing B in k.

Since we have that $\mathbf{eval}(e, \mathbb{D}(\mathtt{p}))$ maps variables in e into paths contained in $\mathbb{D}(\mathtt{p})$, the executions of $\mathbf{eval}(e, \mathbb{D}(\mathtt{p}))$ and $\mathbf{eval}(e, D(\mathtt{p}))$ return the same value, so that $v = t_m$ and the corresponding message in the respective queues in D' and $\mathbb{D}'$ are the same.

From Theorem 1 we know we can find Γ' such that $\Gamma' \vdash D', C'$. We can build Γ' from Γ where the only difference is in the reduction of the local type of $k[A]$ due to the consumption of the send action by the choreography.

Let $\langle\!\langle D' \rangle\!\rangle^{\Gamma'} = \mathbb{D}^*$. We need to demonstrate that $\mathbb{D}' = \mathbb{D}^*$. We divide the analysis over the three "foreach" loops of the encoding algorithm from Figure 12 of the article. As mentioned earlier, the locations of the processes found in $\mathbb{D}$ and $\mathbb{D}'$ are the same and this is true also for $\mathbb{D}^*$ since Γ' has the same list of processes and locations of Γ. Similarly, Γ and Γ' have the same typing of process variables such that the state of processes in $\mathbb{D}'$ (from $\mathbb{D}$) and $\mathbb{D}^*$ are the same. Finally, the only difference between $\mathbb{D}$ and $\mathbb{D}'$ is the new message in the queue $l : t_c$. Let us impose, w.l.o.g., that the **fresh** function in the encoding assigns the same correlation keys to each ordered pair of roles under the same session found under Γ' as it did for the keys found under Γ[2]. Then, also $\mathbb{D}^*$ contains all the locations and correlation keys under the states of its processes and the messages in the existing queues as found in $\mathbb{D}$, except it carries the new message from the reduction under queue $l : t_c$ as $\mathbb{D}'$ does.

Case $\lfloor^{\mathsf{C}}|_{\mathsf{Recv}}\rceil$

we know that $D, C = D,\ k\colon \mathtt{A} \longrightarrow \mathtt{q}[\mathtt{B}].\{o_i(x_i); C_i\}_{i \in I}$ and we can write the derivation

$$\dfrac{j \in I \qquad \dfrac{D(k[\mathtt{A}\rangle\mathtt{B}]) = (o_j, v) :: \tilde{m}}{D, k\colon \mathtt{A} \longrightarrow \mathtt{q}[\mathtt{B}].o_j(x_j) \blacktriangleright D\big[k[\mathtt{A}\rangle\mathtt{B}] \mapsto \tilde{m}\big]\big[\mathtt{q} \mapsto D(\mathtt{q})[x_j \mapsto v]\big]}\ \lfloor^{\mathsf{D}}|_{\mathsf{Recv}}\rceil}{D,\ k\colon \mathtt{A} \longrightarrow \mathtt{q}[\mathtt{B}].\{o_i(x_i); C_i\}_{i \in I} \quad \to \quad D\big[k[\mathtt{A}\rangle\mathtt{B}] \mapsto \tilde{m}\big]\big[\mathtt{q} \mapsto D(\mathtt{q})[x_j \mapsto v]\big],\ C_j}\ \lfloor^{\mathsf{C}}|_{\mathsf{Recv}}\rceil$$

where $C' = C_j$ and $D' = D\big[k[\mathtt{A}\rangle\mathtt{B}] \mapsto \tilde{m}\big]\big[\mathtt{q} \mapsto D(\mathtt{q})[x \mapsto v]\big]$. Then, given $\mathbb{D} = \langle\!\langle D \rangle\!\rangle^{\Gamma}$ we can write the derivation

$$\dfrac{j \in I \qquad \dfrac{t_c = \underline{k.A.B}(\ \mathbb{D}(\mathtt{q})\ ) \quad \mathtt{q} \in \mathbb{D}(l) \quad \mathbb{D}(l : t_c) = (o_j, t_m) :: \tilde{m} \quad \mathbb{D}_1 = \mathbb{D}[\ l : t_c \mapsto \tilde{m}\ ]}{\mathbb{D}, k\colon \mathtt{A} \longrightarrow \mathtt{q}[\mathtt{B}].o_j(x_j) \blacktriangleright \mathbb{D}'\Big[\ \mathtt{q} \mapsto \mathbb{D}_1(\mathtt{q}) \triangleleft (\ \underline{x_j}, t_m\ )\ \Big]}\ \lfloor^{\mathbb{D}}|_{\mathsf{Recv}}\rceil}{\mathbb{D},\ k\colon \mathtt{A} \longrightarrow \mathtt{q}[\mathtt{B}].\{o_i(x_i); C_i\}_{i \in I} \quad \to \quad \mathbb{D}_1\Big[\ \mathtt{q} \mapsto \mathbb{D}_1(\mathtt{q}) \triangleleft (\ \underline{x_j}, t_m\ )\ \Big],\ C_j}\ \lfloor^{\mathsf{C}}|_{\mathsf{Recv}}\rceil$$

where we know we select operation o_j since, from Definition 2 of the article, we find the same messages (both label- and value-wise) between D and its encoding $\mathbb{D}$. From the derivation, we have $C' = C_j$ and $\mathbb{D}' = \mathbb{D}_1\Big[\ \mathtt{q} \mapsto \mathbb{D}_1(\mathtt{q}) \triangleleft (\ \underline{x_j}, t_m\ )\ \Big],\ C_j$.

[2]The encoding procedure abstracts away from this detail since the important point is that the correlation keys are consistently distributed, within the same deployment, among the processes that participate in the sessions.

Following the same reasoning as the previous case, we know that both communications take place correspondingly and, thus, the value held under variable x_j in D' and path $\underline{x_j}$ in $\mathbb{D}'$ in the state of process q is the same. Similarly, we remove from both D and $\mathbb{D}$ the first message in the queue, leaving the corresponding remainder as per Definition 2 of the article. As in the previous case, we take Γ' such that $\Gamma' \vdash D', C'$ and Γ' builds from Γ where we reduce the interested local typing corresponding to the receive action and add the typing of the new variable x_j under the state of q. Then, we can demonstrate that $\langle\!\langle D' \rangle\!\rangle^{\Gamma'} = \mathbb{D}'$. The locations of the processes found in $\langle\!\langle D' \rangle\!\rangle^{\Gamma'}$ and $\mathbb{D}'$ are the same. This is true also for process states, except that Γ' adds the typing of x_j under p. As we mentioned, we find the value of x_j in D' under the corresponding path $\underline{x_j}$ under $\langle\!\langle D' \rangle\!\rangle^{\Gamma'}$ as well as under $\mathbb{D}'$. Finally, assuming (w.l.o.g., see case above) that $\langle\!\langle D' \rangle\!\rangle^{\Gamma'}$ maintains the same session-correlation-keys correspondence of $\mathbb{D}$, we find under $l : t_c$ the updated message queue $\tilde{m}$ as found in $\mathbb{D}'$.

Case $\lfloor^{\mathsf{C}}|_{\mathsf{Start}}\rceil$

we know that $C = \mathtt{start}\ k : \mathsf{p}[\mathtt{A}] \Leftrightarrow \widetilde{l.\mathsf{q}[\mathtt{B}]}; C_c$ and we can write the derivation

$$\dfrac{D\#k', \tilde{\mathsf{r}} \qquad \delta = \mathtt{start}\ k' : \mathsf{p}[\mathtt{A}] \Leftrightarrow \widetilde{l.\mathsf{q}[\mathtt{B}]} \qquad \dfrac{D' = D\big[\mathsf{q} \mapsto \varnothing \mid \mathsf{q} \in \tilde{\mathsf{q}}\big]\big[k'[\mathtt{C}\rangle\mathtt{E}] \mapsto \varepsilon \mid \{\mathtt{C},\mathtt{E}\} \subseteq \{\mathtt{A},\tilde{\mathtt{B}}\}\big]}{D, \delta \blacktriangleright D'}\ \lfloor^{\mathsf{D}}|_{\mathsf{Start}}\rceil}{D,\ \mathtt{start}\ k : \mathsf{p}[\mathtt{A}] \Leftrightarrow \widetilde{l.\mathsf{q}[\mathtt{B}]}; C \quad \rightarrow \quad D',\ C[k'/k][\tilde{\mathsf{r}}/\tilde{\mathsf{q}}]}\ \lfloor^{\mathsf{C}}|_{\mathsf{Start}}\rceil$$

and $C' = C_c[k'/k][\tilde{\mathsf{r}}/\tilde{\mathsf{q}}]$ whereas, given $\mathbb{D} = \langle\!\langle D \rangle\!\rangle^{\Gamma}$,

$$\dfrac{D\#k', \tilde{\mathsf{r}} \qquad \delta = \mathtt{start}\ k' : \mathsf{p}[\mathtt{A}] \Leftrightarrow \widetilde{l.\mathsf{q}[\mathtt{B}]} \qquad \dfrac{\mathsf{p} \in \mathbb{D}(l) \qquad \Delta}{\mathbb{D}, \delta \blacktriangleright \mathbb{D}_3\big[\ \mathsf{q}_h \mapsto \{\underline{k'} : t\}\ \big]_{h \in \{2,\ldots,n\}}}\ \lfloor^{\mathbb{D}}|_{\mathsf{Start}}\rceil}{\mathbb{D},\ \mathtt{start}\ k : \mathsf{p}[\mathtt{A}] \Leftrightarrow \widetilde{l.\mathsf{q}[\mathtt{B}]}; C \quad \rightarrow \quad \mathbb{D}_3\big[\ \mathsf{q}_h \mapsto \{\underline{k} : t\}\ \big]_{h \in \{2,\ldots,n\}},\ C[k'/k][\tilde{\mathsf{r}}/\tilde{\mathsf{q}}]}\ \lfloor^{\mathsf{C}}|_{\mathsf{Start}}\rceil$$

where Δ is

$$\dfrac{\begin{array}{c} \mathsf{q}_1 \in \mathbb{D}\ ① \qquad j \in I \setminus \{i\} \qquad \underline{B_i.l}(t) = l_i ② \qquad \underline{B_i.B_j}(t) = t_{ij} ③ \qquad l_j : t_{ij} \notin \mathbb{D} ④ \\ \mathbb{D}_1 = \mathbb{D}\big[\ l_i \mapsto \mathbb{D}(l_i) \cup \{\mathsf{q}_i\}\ \big] ⑤ \quad \mathbb{D}_2 = \mathbb{D}_1\big[\ l_i : t_{ij} \mapsto \varepsilon\ \big] ⑥ \quad \mathbb{D}_3 = \mathbb{D}_2\big[\ \mathsf{q}_1 \mapsto \mathbb{D}''(\mathsf{q}_1) \triangleleft (\underline{k'}, t)\ \big] ⑦ \end{array}}{\mathbb{D}, \mathbf{sup}(\ k', \{\ l_i.\mathsf{q}_i[\mathtt{B}_i]\ \}_{i \in I}\ ) \blacktriangleright \mathbb{D}_3\big[\ \mathsf{q}_h \mapsto \{\underline{k'} : t\}\ \big]_{h \in \{2,\ldots,n\}} ⑧}\ \lfloor^{\mathbb{D}}|_{\mathsf{Sup}}\rceil$$

with $C' = C_c[k'/k][\tilde{\mathsf{r}}/\tilde{\mathsf{q}}]$ and $\mathbb{D}' = \mathbb{D}_3\big[\ \mathsf{q}_h \mapsto \{\underline{k'} : t\}\ \big]_{h \in \{2,\ldots,n\}}$.

To demonstrate that $\langle\!\langle D' \rangle\!\rangle^{\Gamma} = \mathbb{D}'$ we follow the same reasoning as in the previous two cases. We build Γ' from Γ such that $\Gamma' \vdash D', C'$. In particular, Γ' adds all the typings for the new session k' and the new processes $\tilde{\mathsf{r}}$ (process variables, local typings, buffers, locations, etc.) following the reductions above.

In D', we find the new processes $\tilde{\mathsf{r}}$ with an empty state and the empty queues for the session. This effect is mirrored in $\mathbb{D}'$, where the only data in the state of the new processes is the one found under the path $\underline{k'}$, which contains the data needed to implement the communication logic seen in the first two rules (for locating the addressee and, subsequently, the queue where it expects messages from the sender). The steps ①–⑧ realise, for one session, the same routine for implementing correlation-based communication given the names of the processes, roles, locations, and the session involved found in the encoding in Figure 12 of the article.

As done in the previous cases, we can demonstrate that $\langle\!\langle D' \rangle\!\rangle^{\Gamma'} = \mathbb{D}'$ by having (w.l.o.g.) the **fresh** function 1) assign the same correlation keys to each ordered pair of roles under the same session found under Γ' as it did for the keys found under Γ and 2) assign the same correlation keys to each ordered pair of roles under the newly created session k', as performed by rule $\lfloor^{\mathbb{D}}|_{\mathsf{Sup}}\rceil$.

Case $\lfloor^{\mathsf{C}}|_{\mathsf{PStart}}\rceil$

Similar to the proof of case $\lfloor^{\mathsf{C}}|_{\mathsf{Start}}\rceil$.

Case $\lfloor^{\mathsf{C}}|_{\mathsf{Cond}}\rceil$

We know that $C = \texttt{if}\ \mathtt{p}.e\ \{C_1\}\ \texttt{else}\ \{C_2\}$ and we can write the derivation

$$\frac{i = 1 \text{ if } \mathbf{eval}(e, D(\mathtt{p})) = \text{ true}, i = 2 \text{ otherwise}}{D,\ \texttt{if}\ \mathtt{p}.e\ \{C_1\}\ \texttt{else}\ \{C_2\} \quad \to \quad D,\ C_i}\ \lfloor^{\mathsf{C}}|_{\mathsf{Cond}}\rceil$$

We develop the case only considering $\mathbf{eval}(e, D(\mathtt{p})) = \text{true}$ as $\mathbf{eval}(e, D(\mathtt{p})) = \text{false}$ is similar; then, $C' = C_1$ and $D' = D$.

Given $\mathbb{D} = \langle\!\langle D \rangle\!\rangle^{\Gamma}$, similarly to the previous derivation

$$\frac{i = 1 \text{ if } \mathbf{eval}(e, \mathbb{D}(\mathtt{p})) = \text{ true}, i = 2 \text{ otherwise}}{\mathbb{D},\ \texttt{if}\ \mathtt{p}.e\ \{C_1\}\ \texttt{else}\ \{C_2\} \quad \to \quad \mathbb{D},\ C_i}\ \lfloor^{\mathsf{C}}|_{\mathsf{Cond}}\rceil$$

where we know that $\mathbf{eval}(e, D(\mathtt{p})) = \mathbf{eval}(e, \mathbb{D}(\mathtt{p}))$ per the properties we required on the **eval** function and, thus, $C' = C_1$ and $\mathbb{D}' = \mathbb{D}$.

We take $\Gamma' = \Gamma$ and obtain (under the condition on the **fresh** function of the previous cases) that $\langle\!\langle D' \rangle\!\rangle^{\Gamma} = \mathbb{D}'$.

Case $\lfloor^{\mathsf{C}}|_{\mathsf{Ctx}}\rceil$, **Case** $\lfloor^{\mathsf{C}}|_{\mathsf{Par}}\rceil$, and **Case** $\lfloor^{\mathsf{C}}|_{\mathsf{Eq}}\rceil$

Proved by the induction hypothesis.

□

Proof (Soundness)

The proof is done by induction on the derivation of $\langle\!\langle D \rangle\!\rangle^{\Gamma}, C \to \mathbb{D}, C'$. We avoid repeating the breakdown of all the cases because the latter pair-wise unfold following the structure of the corresponding cases in the proof of (Completeness), although we swap the challenge and response reductions, which, here, happen respectively on $\langle\!\langle D \rangle\!\rangle^{\Gamma}, C$ and D, C.

The observations written case by case hold for their corresponding ones for (Soundness) since we have D and Γ define the content of $\langle\!\langle D \rangle\!\rangle^{\Gamma}$, which we then update following the rules of the semantics.

□

3.4 Proof of Endpoint Projection

To prove our result on the Endpoint Projection we first define the minimal typing system $\vdash_{\mathtt{min}}$ for FC.

3.4.1 Minimal Typing

We recall the definition of subtyping for local and global types (see Definitions 8 and 9), which we extend to set inclusion and point-wise to *i*) the typing of services (i.e., of kind $\tilde{l} \colon G\langle \mathtt{A}|\tilde{\mathtt{B}}|\tilde{\mathtt{C}}\rangle$) and *ii*) the typing of sessions, respectively. Given two types G and G', we denote their least upper bound wrt $\prec$ with $G \triangledown G'$ (the same for local types and typing environments).

We define the minimal typing system $\vdash_{\mathtt{min}}$ on this notion of subtyping. The minimal typing uses the minimal global and local types for typing sessions and services such that the projection of the choreography is still typable. We report the rules for minimal typing in Figure 11.

Proposition 1 (Existence of Minimal Typing)**.** Let $\Gamma \vdash D, C$, then there exists Γ_0 such that $\Gamma_0 \vdash D, C$ and for each $\Gamma' \vdash D, C$ we have that $\Gamma_0 \prec \Gamma'$. The environment Γ_0 can be algorithmically calculated from C and is called the minimal typing of C.

$$\frac{\Gamma, \mathbf{init}(\,\widetilde{\mathsf{r}[\mathtt{C}]}, k, G\,) \vdash_{\mathtt{min}} C \qquad \widetilde{\mathsf{r}[\mathtt{C}]} = \mathsf{p}[\mathtt{A}], \widetilde{\mathsf{q}[\mathtt{B}]} \qquad \tilde{\mathsf{q}} \notin \Gamma \qquad \tilde{l} \notin \Gamma}{\Gamma, \tilde{l} : G\langle \mathtt{A}|\tilde{\mathtt{B}}|\tilde{\mathtt{B}}\rangle \vdash_{\mathtt{min}} \mathtt{start}\ k : \mathsf{p}[\mathtt{A}] \leftrightarrow \widetilde{l.\mathsf{q}[\mathtt{B}]}; C} \;\lfloor^{\mathsf{Min}}|_{\mathsf{Start1}}\rceil$$

$$\frac{\Gamma, \tilde{l} : G\langle \mathtt{A}|\tilde{\mathtt{B}}|\tilde{\mathtt{B}}\rangle, \mathbf{init}(\,\widetilde{\mathsf{r}[\mathtt{C}]}, k, G'\,) \vdash_{\mathtt{min}} C \qquad \widetilde{\mathsf{r}[\mathtt{C}]} = \mathsf{p}[\mathtt{A}], \widetilde{\mathsf{q}[\mathtt{B}]} \qquad \tilde{\mathsf{q}} \notin \Gamma}{\Gamma, \tilde{l} : G \triangledown G' \langle \mathtt{A}|\tilde{\mathtt{B}}|\tilde{\mathtt{B}}\rangle \vdash_{\mathtt{min}} \mathtt{start}\ k : \mathsf{p}[\mathtt{A}] \leftrightarrow \widetilde{l.\mathsf{q}[\mathtt{B}]}; C} \;\lfloor^{\mathsf{Min}}|_{\mathsf{Start2}}\rceil$$

$$\frac{\Gamma, \mathsf{p} : k[\mathtt{A}], k[\mathtt{A}] : [\![G]\!]_{\mathtt{A}} \vdash_{\mathtt{min}} C \qquad \tilde{l} \notin \Gamma}{\Gamma, \tilde{l} : G\langle \mathtt{A}|\tilde{\mathtt{B}}|\varnothing\rangle \vdash_{\mathtt{min}} \mathtt{req}\ k : \mathsf{p}[\mathtt{A}] \leftrightarrow \widetilde{l.\mathtt{B}}; C} \;\lfloor^{\mathsf{Min}}|_{\mathsf{Req1}}\rceil \qquad \frac{\Gamma, \tilde{l} : G\langle \mathtt{A}|\tilde{\mathtt{B}}|\varnothing\rangle, \mathsf{p} : k[\mathtt{A}], k[\mathtt{A}] : [\![G']\!]_{\mathtt{A}} \vdash_{\mathtt{min}} C}{\Gamma, \tilde{l} : G \triangledown G' \langle \mathtt{A}|\tilde{\mathtt{B}}|\varnothing\rangle \vdash_{\mathtt{min}} \mathtt{req}\ k : \mathsf{p}[\mathtt{A}] \leftrightarrow \widetilde{l.\mathtt{B}}; C} \;\lfloor^{\mathsf{Min}}|_{\mathsf{Req2}}\rceil$$

$$\frac{\tilde{l} \subseteq \tilde{l}' \qquad \Gamma, \mathbf{init}(\,\widetilde{\mathsf{q}[\mathtt{C}]}, k, G\,) \vdash_{\mathtt{min}} C \qquad \tilde{\mathsf{q}} \notin \Gamma \qquad \tilde{l} \notin \Gamma}{\Gamma, \tilde{l}' : G\langle \mathtt{A}|\tilde{\mathtt{B}}|\tilde{\mathtt{C}}\rangle \vdash_{\mathtt{min}} \mathtt{acc}\ k : \widetilde{l.\mathsf{q}[\mathtt{C}]}; C} \;\lfloor^{\mathsf{Min}}|_{\mathsf{Acc}}\rceil$$

$$\frac{\Gamma_1 \triangledown \Gamma_2 \vdash \mathsf{p}.e : \mathbf{bool} \qquad \Gamma_1 \vdash_{\mathtt{min}} C_1 \qquad \Gamma_2 \vdash_{\mathtt{min}} C_2}{\Gamma_1 \triangledown \Gamma_2 \vdash_{\mathtt{min}} \mathtt{if}\ \mathsf{p}.e\ \{C_1\}\ \mathtt{else}\ \{C_2\}} \;\lfloor^{\mathsf{Min}}|_{\mathsf{Cond}}\rceil$$

$$\frac{\Gamma \vdash \mathsf{p} : k[\mathtt{A}], \mathsf{q} : k[\mathtt{B}] \qquad \Gamma \vdash \mathsf{p}.e : U \qquad \Gamma, \mathsf{q}.x : U, k[\mathtt{A}] : T, k[\mathtt{B}] : T' \vdash_{\mathtt{min}} C}{\Gamma, k[\mathtt{A}] : \oplus\, \mathtt{B}.\{o(U); T\}, k[\mathtt{B}] : \&\mathtt{A}.\{o(U); T'\} \vdash_{\mathtt{min}} k : \mathsf{p}[\mathtt{A}].e \longrightarrow \mathsf{q}[\mathtt{B}].o(x); C} \;\lfloor^{\mathsf{Min}}|_{\mathsf{Com}}\rceil$$

$$\frac{\Gamma \vdash \mathsf{p} : k[\mathtt{A}] \qquad \mathsf{q} : k[\mathtt{B}] \notin \Gamma \qquad \Gamma \vdash \mathsf{p}.e : U \qquad \Gamma, k[\mathtt{A}] : T \vdash_{\mathtt{min}} C}{\Gamma, k[\mathtt{A}] : \oplus\, \mathtt{B}.\{o(U); T\} \vdash_{\mathtt{min}} k : \mathsf{p}[\mathtt{A}].e \longrightarrow \mathtt{B}.o; C} \;\lfloor^{\mathsf{Min}}|_{\mathsf{Send}}\rceil$$

$$\frac{\Gamma \vdash \mathsf{q} : k[\mathtt{B}] \qquad \mathsf{p} : k[\mathtt{A}] \notin \Gamma \qquad \Gamma, \mathsf{q}.x : U, k[\mathtt{B}] : T \vdash_{\mathtt{min}} C}{\Gamma, k[\mathtt{B}] : \&\mathtt{A}.\{o(U); T\} \vdash_{\mathtt{min}} k : \mathtt{A} \longrightarrow \mathsf{q}[\mathtt{B}].o(x); C} \;\lfloor^{\mathsf{Min}}|_{\mathsf{Recv}}\rceil$$

$$\frac{\begin{array}{c}\Gamma_x(X) = \Gamma'_x(X) \text{ if } X \in \mathbf{dom}(\Gamma_x) \cap \mathbf{dom}(\Gamma'_x) \qquad \nexists\, k''[\mathtt{A}''] \in \mathbf{dom}(\Gamma \triangledown \Gamma') \\ X \notin \mathbf{dom}(\Gamma \triangledown \Gamma') \quad \Gamma'_x \rhd_X (\Gamma', \widetilde{k'[\mathtt{A}'] : T'}), C' \qquad \Gamma_x \rhd_X (\Gamma, \widetilde{k[\mathtt{A}] : T}), C \qquad \Gamma'|_{\mathbf{locs}} \subseteq \Gamma\end{array}}{(\Gamma \triangledown \Gamma'), \mathsf{solve}(\widetilde{k[\mathtt{A}] : T} \triangledown \widetilde{k'[\mathtt{A}'] : T'}, \mathbf{t}_X) \vdash_{\mathtt{min}} \mathtt{def}\ X = C'\ \mathtt{in}\ C} \;\lfloor^{\mathsf{Min}}|_{\mathsf{Def}}\rceil$$

$$\frac{\Gamma_1 \vdash_{\mathtt{min}} C_1 \qquad \Gamma_2 \vdash_{\mathtt{min}} C_2}{\Gamma_1, \Gamma_2 \vdash_{\mathtt{min}} C_1 \mid C_2} \;\lfloor^{\mathsf{Min}}|_{\mathsf{Par}}\rceil$$

$$\frac{\Gamma, \Gamma_x, X : \Gamma_x \vdash_{\mathtt{min}} C}{\Gamma_x, X : \Gamma_x \rhd_X \Gamma, C} \;\lfloor^{\mathsf{Min}}|_{\mathsf{D1}}\rceil \qquad \frac{X \notin \mathbf{dom}(\Gamma_x) \qquad \Gamma, \Gamma_x \vdash_{\mathtt{min}} C}{\Gamma_x \rhd_X \Gamma, C} \;\lfloor^{\mathsf{Min}}|_{\mathsf{D2}}\rceil$$

$$\frac{\Gamma = \mathsf{ownerships} \cup \mathsf{sessions} \cup \mathsf{vars} \qquad k[\mathtt{A}] \in \mathsf{sessions} \qquad k[\mathtt{A}] : \mathsf{end}}{\Gamma \vdash_{\mathtt{min}} \mathbf{0}} \;\lfloor^{\mathsf{Min}}|_{\mathsf{End}}\rceil$$

$$\frac{\begin{array}{c}\Gamma = \mathsf{vars} \cup \mathsf{ownerships} \qquad \widetilde{k'[\mathtt{A}']} = \mathsf{sessions} \setminus \{\widetilde{k[\mathtt{A}]}\} \\ \Gamma' = \mathsf{vars}(X) \cup \mathsf{ownerships}(X) \qquad \widetilde{k[\mathtt{A}]} = \mathsf{sessions}(\mathsf{X}) \qquad \Gamma' \subseteq \Gamma\end{array}}{\Gamma, \widetilde{k[\mathtt{A}]} : \mathbf{t}_X, \widetilde{k'[\mathtt{A}']} : \mathsf{end}, X : (\Gamma', \widetilde{k[\mathtt{A}]} : \mathbf{t}_X) \vdash_{\mathtt{min}} X} \;\lfloor^{\mathsf{Min}}|_{\mathsf{Call}}\rceil$$

$$\frac{\mathbf{pco}(\Gamma, \Gamma') \qquad \Gamma \vdash D \qquad \Gamma' \vdash_{\mathtt{min}} C}{\Gamma, \Gamma' \vdash_{\mathtt{min}} D, C} \;\lfloor^{\mathsf{Min}}|_{\mathsf{DC}}\rceil$$

Figure 11. Frontend Choreographies, Minimal typing rules

Proof of Existence of Minimal Typing The proof is standard and proceeds by induction on the rules in Figure 11, defining the minimal typing system $\Gamma \vdash_{\mathtt{min}} D, C$.

As in Carbone and Montesi (2013); Montesi and Yoshida (2013), our focus is on the reconstruction of global/local types, thus we leave the reconstruction of variable types undefined (which it is entirely standard, e.g., see Pierce (2002)).

We give the intuition behind each case corresponding to the derivation on the rules. $\lfloor^{\mathsf{Min}}|_{\mathsf{Start1}}\rceil$ and $\lfloor^{\mathsf{Min}}|_{\mathsf{Start2}}\rceil$ type the starting of sessions. The difference between $\lfloor^{\mathsf{Min}}|_{\mathsf{Start1}}\rceil$ and $\lfloor^{\mathsf{Min}}|_{\mathsf{Start2}}\rceil$ is that, when $\lfloor^{\mathsf{Min}}|_{\mathsf{Start1}}\rceil$ applies, the service typing of $\tilde{l}$ is not used anymore in C, and thus its typing is dropped to guarantee minimality. Contrarily, in $\lfloor^{\mathsf{Min}}|_{\mathsf{Start2}}\rceil$ the service typing of $\tilde{l}$ is used in the continuation C. In the rule, we consider the minimal global type $G \triangledown G'$ where G' is minimal in session k and G is minimal in the typing of the continuation C.

Rules $\lfloor^{\mathsf{Min}}|_{\mathsf{Req1}}\rceil$ and $\lfloor^{\mathsf{Min}}|_{\mathsf{Req2}}\rceil$ mirror a similar relationship, where in the first rule we drop the typing of $\tilde{l}$, not used in the continuation C, while in the second we consider $G \triangledown G'$. Note that Rule $\lfloor^{\mathsf{Min}}|_{\mathsf{Acc}}\rceil$ directly drops the typing of $\tilde{l}$ in the typing of the continuation. We do this because we have that by rule $\lfloor^{\mathsf{T}}|_{\mathsf{Acc}}\rceil$ no subsequent term (acc) or $(start)$ on the same locations $\tilde{l}$ is typable (and hence cannot be present in C, well-typed). The same holds for subsequent (req) terms on $\tilde{l}$, which could not be paired with a complementary (acc).

In $\lfloor^{\mathsf{Min}}|_{\mathsf{Cond}}\rceil$ we consider $\Gamma_1 \triangledown \Gamma_2$ to determine the least upper bound of receive types. Rules $\lfloor^{\mathsf{Min}}|_{\mathsf{Com}}\rceil$, $\lfloor^{\mathsf{Min}}|_{\mathsf{Send}}\rceil$, and $\lfloor^{\mathsf{Min}}|_{\mathsf{Recv}}\rceil$ type receptions with a singleton branching local type. Rule $\lfloor^{\mathsf{Min}}|_{\mathsf{Par}}\rceil$ is standard.

Also in rule $\lfloor^{\mathsf{Min}}|_{\mathsf{Def}}\rceil$ we consider the least upper bound of Γ and Γ' respectively typing the continuation C and the body of procedure X. In addition, we also consider the least upper bound of the local typings T and T', on which we apply function solve. Function solve is standard (cf. Carbone et al. (2012); Carbone and Montesi (2013)) and solves the equations $\mathbf{t}_X = T$ for each T in $\widetilde{k[\mathtt{A}] : T}$ where, if $\mathbf{t}_X$ appears in T, the corresponding component is $\mathtt{rec}\ \mathbf{t}.T_X$, or T otherwise. Rule $\lfloor^{\mathsf{Min}}|_{\mathsf{Def}}\rceil$ uses rules $\lfloor^{\mathsf{Min}}|_{\mathsf{D1}}\rceil$ and $\lfloor^{\mathsf{Min}}|_{\mathsf{D2}}\rceil$ to determine the content of Γ_x and Γ'_x to respectively minimally type the continuation C and the body of procedure X. Indeed, when rule $\lfloor^{\mathsf{Min}}|_{\mathsf{D1}}\rceil$ applies, the choreography C uses the typing $X : \Gamma_x$, otherwise $\lfloor^{\mathsf{Min}}|_{\mathsf{D2}}\rceil$ applies and the minimal type does not contain the typing for X. Finally, in case both the typing of C and of C' type X (i.e., X in $\mathbf{dom}(\Gamma_x) \cap \mathbf{dom}(\Gamma'_x)$), their judgements coincide.

Rules $\lfloor^{\mathsf{Min}}|_{\mathsf{End}}\rceil$ and $\lfloor^{\mathsf{Min}}|_{\mathsf{Call}}\rceil$ use some auxiliary information, obtainable by a preliminary top-down visit of the choreography syntax tree (cf. Carbone et al. (2012); Carbone and Montesi (2013)). Specifically, vars, ownerships, and sessions are respectively the variable, the ownership, and the session typings of the choreography whose type is being inferred. Similarly, $\mathsf{vars}(X)$, $\mathsf{ownerships}(X)$, and $\mathsf{sessions}(X)$ yield respectively the same kind of information regarding the body of procedure X (i.e., obtained inspecting the body of the inner-most recursive procedure X). In the rules, in $\lfloor^{\mathsf{Min}}|_{\mathsf{End}}\rceil$ we check that in Γ reside only those ownership, variable, and session typings present in the typed choreography and that all sessions (i.e., their local types) are terminated. In rule $\lfloor^{\mathsf{Min}}|_{\mathsf{Call}}\rceil$, *i*) all sessions outside X must be terminated and those inside X agree on $\mathbf{t}_X$ and *ii*) Γ and Γ' contain only appropriate variable and ownership typings and agree on their judgement ($\Gamma' \subseteq \Gamma$).

Rule $\lfloor^{\mathsf{Min}}|_{\mathsf{DC}}\rceil$ defines minimal typing for running choreographies.

□

3.4.2 Typing Projection

Here we define the projection of typing environments, which is used to prove that, given the minimal typing environment Γ of a choreography C, from Γ we can build the minimal typing environment for the EPP of C.

To do that, we have to account for two peculiarities (as defined in Section 6.2 of the article) of our EPP:

- it merges in the output choreography the behaviour of many service processes into one process. Hence, to guarantee typing and minimality we have to merge typings related to service processes on the same location into the same (and only) service process present in $[\![C]\!]$;
- it projects recursive definitions of the same procedure on different processes, e.g., if in C there are processes $\mathsf{p}_1, \ldots, \mathsf{p}_n$ and procedure X, in the EPP we will find procedures $X_{\mathsf{p}_1}, \ldots, X_{\mathsf{p}_n}$. Thus, we replace the definition typing of any procedure X in $\mathbf{dom}(\Gamma)$ with the typings of its projections X_{p_1}, $\ldots$, X_{p_n}.

To indicate the projection of a typing environment Γ wrt to its typed choreography C, we write $[\![\Gamma]\!]^C$. To define $[\![\Gamma]\!]^C$ (and also later in this proof) we use the typing environment filtering operator $\Gamma|_{\mathsf{p}}$ defined as

$$\Gamma|_{\mathsf{p}} = \begin{cases} \{\ \mathsf{p}.x : U \mid \mathsf{p}.x : U \in \Gamma\ \} & \cup \\ \{\ \mathsf{p} : k[\mathtt{A}], k[\mathtt{A}] : T\} \mid \{\ \mathsf{p} : k[\mathtt{A}], k[\mathtt{A}] : T\ \} \subseteq \Gamma\ \} & \cup \\ \{\ \tilde{l} : G\langle \mathtt{A}|\tilde{\mathtt{B}}|\tilde{\mathtt{C}}\rangle \mid \tilde{l} : G\langle \mathtt{A}|\tilde{\mathtt{B}}|\tilde{\mathtt{C}}\rangle \in \Gamma\ \} & \cup \\ \{\ X_{\mathsf{p}} : \Gamma_x \mid X_{\mathsf{p}} : \Gamma_x\ \in\ \Gamma\ \} \end{cases}$$

Definition 11 (Typing Projection)**.** Let $\Gamma \vdash C$, the projection of Γ wrt to C, written $[\![\Gamma]\!]^C$, is defined as:

$$[\![\Gamma]\!]^C \quad = \quad \underbrace{\left\{ \bigcup_{\mathsf{q} \in \lfloor C \rfloor_l} \underbrace{[\![\Gamma]\!]_{\mathsf{q}} [\mathsf{p}/\mathsf{q}]}_{i.i)} \mid \underbrace{\mathsf{p} \in \lfloor C \rfloor_l \cap \mathbf{pn}([\![C]\!])}_{i.ii)} \wedge l \in \{\tilde{l}\} \ \wedge\ \tilde{l} \in \mathbf{dom}(\Gamma) \right\}}_{i)}, \underbrace{\{\ [\![\Gamma]\!]_{\mathsf{r}} \mid \mathsf{r}\ \in\ \mathbf{fp}([\![C]\!])\}}_{ii)}$$

$$[\![\Gamma]\!]_{\mathsf{p}} \quad = \quad \underbrace{\left(\Gamma|_{\mathsf{p}} \setminus \{X : \Gamma_x \mid \Gamma \vdash X : \Gamma_x\}\right)}_{iii)}, \underbrace{\left\{X_{\mathsf{p}} : [\![\Gamma_x]\!]_{\mathsf{p}} \mid \Gamma \vdash X : \Gamma_x\right\}}_{iv)}$$

As mentioned above, in the definition of $[\![\Gamma]\!]^C$ we distinguish two kinds of projections: the one on service processes *i)* and the one on active processes *ii)*. In the first case, we unify the projection on service processes at the same location in C (i.e., in $\lfloor C \rfloor_l$). To do that in a consistent way, wrt to the EPP of C we:

- obtain the identifier of process p *i.i)*, the only service process at location l that is present in $[\![C]\!]$ (and hence the one that merges the behaviour of all service processes in C at l);
- get the projection of Γ on a service process q ($[\![G]\!]_{\mathsf{q}}$) in $\lfloor C \rfloor_l$;
- we rename all process-related typings in $[\![\Gamma]\!]_{\mathsf{q}}$ to correspond to process p (by abusing the notation $[\![\Gamma]\!]_{\mathsf{q}} [\mathsf{p}/\mathsf{q}]$) *i.ii)*;
- we merge all the resulting, renamed typing environments into a single typing environment for process p.

Finally, the projection of typing environment Γ on process p, written $[\![\Gamma]\!]_{\mathsf{p}}$ corresponds to the union of *iii)* the typing in Γ related to process p, from which we remove the typings of definitions, and *iv)* the projection of the typings of definitions, renamed for process p.

Note the definition of $[\![\Gamma]\!]^C$ is coherent with the definition of process projection (see Definition 4 of the article) in which the rule for projecting (rec) terms is defined as:

$$[\![\mathtt{def}\ X = C'\ \mathtt{in}\ C]\!]_{\mathsf{r}} \quad = \quad \mathtt{def}\ X_{\mathsf{r}} = [\![\ C'[X_{\mathsf{r}}/X]\ ]\!]_{\mathsf{r}}\ \mathtt{in}\ [\![\ C[X_{\mathsf{r}}/X]\ ]\!]_{\mathsf{r}}$$

Similarly, $[\![\Gamma]\!]^C$ generates definition typings for each procedure corresponding to each process in the choreography (assumed to be C). The typings of definitions are guaranteed minimal (as required in Theorem 5).

The only remark regards service typings, which are present in all projected environments, although they might not be used. While having additional, unused service typings does not compromise type checking, we must consider a weakened form of minimality of typing where some unused service typings are allowed. This fact is clearly stated in the definition of the Theorem 5.

3.5 Proof of the Well-Typedness Property of Theorem 2 of the article

To prove the property of well-typedness of Theorem 2 of the article we prove the stronger result of Theorem 5.

Theorem 5 (EPP Typing Preservation)**.** Let D, C be a well-typed running choreography such that $\Gamma \vdash_{\mathtt{min}} D, C$, where $\Gamma = \Gamma_d, \Gamma_c$ such that $\Gamma_d \vdash D$, then $[\![\Gamma_c]\!]^C, \Gamma_d \vdash_{\mathtt{min}} D, [\![C]\!]$ up to service typings.

Intuitively, Theorem 5 subsumes the well-typedness property (1) of Theorem 2 of the article, using the environment projection defined above to provide a minimal typing environment for $[\![C]\!]$ up to some unused service typings.

We define some auxiliary lemmas used in the proof of Theorem 5.

Lemma 7 (Composability of Typing Projections)**.** Let $\Gamma \vdash C$ and $\Gamma = \Gamma', \Gamma''$ then $[\![\Gamma]\!]^C = [\![\Gamma']\!]^C, [\![\Gamma'']\!]^C$.

Proof The proof is by contradiction. The projection $[\![\Gamma]\!]^C$ returns exactly Γ except for the projection of the typings of the procedures, as defined in Definition 11. Hence the projection $[\![\Gamma]\!]^C$ can differ from $[\![\Gamma']\!]^C, [\![\Gamma'']\!]^C$ only on definition typings. However, it is impossible that $[\![\Gamma]\!]^C \neq [\![\Gamma']\!]^C, [\![\Gamma'']\!]^C$. Indeed, there could be only two cases for the partitioning of Γ wrt any definition typing $X \in \mathbf{dom}(\Gamma)$, either:

- *i*) both Γ' and Γ'' type X, in which case, since $\Gamma = \Gamma', \Gamma''$, they must agree on their judgement on X;
- *ii*) the judgement on X is contained only in Γ' or Γ''.

in both cases the projections obtained from X remain the same wrt the one in Γ. □

We prove Lemma 8 that states that given a well-typed choreography C and a typing environment Γ for which $\Gamma \vdash_{\mathtt{min}} C$ then the projection of Γ, $[\![\Gamma]\!]^C$, types minimally the projection of C, $[\![C]\!]$.

Lemma 8 (Choreography EPP Typing Preservation)**.** Let C be a well-typed choreography and let $\Gamma \vdash_{\mathtt{min}} C$ then $[\![\Gamma]\!]^C \vdash_{\mathtt{min}} [\![C]\!]$.

Proof Like for the proof of Theorem 3, we assume our choreographies to be well-sorted. The proof is by induction on the typing derivation of $\Gamma \vdash_{\mathtt{min}} C$.

Case $\lfloor^{\mathsf{Min}}|_{\mathsf{Start1}}\rceil$
From the premises we have $C = \mathtt{start}\ k : \mathtt{p[A]} \mathrel{\triangleleft\!\!\triangleright} \widetilde{l.\mathtt{q[B]}}; C'$. We can partition $\Gamma = \tilde{l} \colon G\langle \mathtt{A}|\tilde{\mathtt{B}}|\tilde{\mathtt{B}}\rangle, \Gamma'$ and we can write the derivation

$$\frac{\Gamma', \mathbf{init}(\widetilde{\mathtt{r[C]}}, k, G) \vdash_{\mathtt{min}} C' \qquad \widetilde{\mathtt{r[C]}} = \mathtt{p[A]}, \widetilde{\mathtt{q[B]}} \qquad \tilde{\mathtt{q}} \notin \Gamma' \qquad \tilde{l} \notin \Gamma'}{\Gamma', \tilde{l} \colon G\langle \mathtt{A}|\tilde{\mathtt{B}}|\tilde{\mathtt{B}}\rangle \vdash_{\mathtt{min}} \mathtt{start}\ k : \mathtt{p[A]} \mathrel{\triangleleft\!\!\triangleright} \widetilde{l.\mathtt{q[B]}}; C'}\ \lfloor^{\mathsf{Min}}|_{\mathsf{Start1}}\rceil$$

Let $\widetilde{l.\mathtt{q[B]}} = l_1.\mathtt{q}_1[\mathtt{B}_1], \cdots, l_n.\mathtt{q}_n[\mathtt{B}_n]$.
Let $\Gamma_c = \Gamma', \mathbf{init}(\widetilde{\mathtt{r[C]}}, k, G)$, from the induction hypothesis we have that $\Gamma_c \vdash_{\mathtt{min}} C'$ and therefore $[\![\Gamma_c]\!]^{C'} \vdash_{\mathtt{min}} [\![C']\!]$.
By its definition $[\![C']\!] \equiv_{\mathsf{C}} C'_s \mid C''$ where

$$C'_s = [\![C']\!]_{\mathsf{p}} \mid [\![C']\!]_{\mathsf{q}_1} \mid \ldots \mid [\![C']\!]_{\mathsf{q}_n}$$

and

$$C'' = \prod_{\mathsf{r} \in \mathbf{fp}(C') \setminus \{\mathsf{p}, \tilde{\mathsf{q}}\}} [\![C']\!]_{\mathsf{r}} \quad \mid \quad \prod_{l} \left(\bigsqcup_{\mathsf{s} \in \lfloor C' \rfloor_l} [\![C']\!]_{\mathsf{s}} \right)$$

We partition $[\![\Gamma_c]\!]^{C'}$ (as per Lemma 7) as

$$[\![\Gamma_c]\!]^{C'} = \Gamma'_{\mathsf{p}}\ ,\ \Gamma'_{\tilde{\mathsf{q}}}\ ,\ \Gamma''$$

where

$$\Gamma'_{\mathsf{p}} = \Gamma''_{\mathsf{p}}, \mathsf{p} \colon k[\mathtt{A}], k[\mathtt{A}] \colon [\![G]\!]_{\mathsf{p}}$$

and

$$\Gamma'_{\tilde{\mathsf{q}}} = \Gamma'_{\mathsf{q}_1}\ ,\ \ldots\ ,\ \Gamma'_{\mathsf{q}_n}$$

where

$$\Gamma'_{\mathsf{q}_i} = \Gamma''_{\mathsf{q}_i}, \mathsf{q}_i \colon k[\mathtt{A}], k[\mathtt{A}] \colon [\![G]\!]_{\mathsf{q}_i}$$

such that we can write the derivation

$$\dfrac{\Gamma'' \vdash_{\mathtt{min}} C'' \qquad \dfrac{\Gamma'_{\mathsf{p}} \vdash_{\mathtt{min}} [\![C']\!]_{\mathsf{p}} \qquad \dfrac{\Gamma'_{\mathsf{q}_1} \vdash_{\mathtt{min}} [\![C']\!]_{\mathsf{q}_1} \qquad \dfrac{\vdots}{\Gamma'_{\mathsf{q}_2}, \ldots, \Gamma'_{\mathsf{q}_n} \vdash_{\mathtt{min}} [\![C']\!]_{\mathsf{q}_2} \mid \ldots \mid [\![C']\!]_{\mathsf{q}_n}}\ \lfloor^{\mathsf{Min}}|_{\mathsf{Par}}\rceil}{\Gamma'_{\mathsf{q}_1}, \Gamma'_{\mathsf{q}_2}, \ldots, \Gamma'_{\mathsf{q}_n} \vdash_{\mathtt{min}} [\![C']\!]_{\mathsf{q}_1} \mid \ldots \mid [\![C']\!]_{\mathsf{q}_n}}\ \lfloor^{\mathsf{Min}}|_{\mathsf{Par}}\rceil}{\Gamma'_{\mathsf{p}}, \Gamma'_{\tilde{\mathsf{q}}} \vdash_{\mathtt{min}} [\![C']\!]_{\mathsf{p}} \mid [\![C']\!]_{\mathsf{q}_1} \mid \ldots \mid [\![C']\!]_{\mathsf{q}_n}}\ \lfloor^{\mathsf{Min}}|_{\mathsf{Par}}\rceil}{\Gamma'', \Gamma'_{\mathsf{p}}, \Gamma'_{\tilde{\mathsf{q}}} \vdash_{\mathtt{min}} C'' \mid C'_s}\ \lfloor^{\mathsf{Min}}|_{\mathsf{Par}}\rceil$$

Since the ownership and session typings for k in Γ_c belong to $\mathbf{init}(\widetilde{\mathsf{r[C]}}, k, G)$ we can write $\Gamma'_{\mathsf{p}} = \Gamma''_{\mathsf{p}}, \mathsf{p}\colon k[\mathtt{A}], k[\mathtt{A}]\colon T$ where Γ''_{p} contains those and only typings (services, ownerships, sessions, etc.) that type minimally the projection of continuation C' for process p.

Since the only difference between Γ and Γ_c are the typings for session k, we have that $\Gamma''_{\mathsf{p}} \subseteq [\![\Gamma]\!]^C$ and also $\Gamma'' \subseteq [\![\Gamma]\!]^C$. The same argument holds for typings Γ'_{q_i}. Indeed, we can partition $[\![\Gamma]\!]^C = \Gamma'', \Gamma''_{\mathsf{p}}, \Gamma''_{\mathsf{q}_1}, \ldots, \Gamma''_{\mathsf{q}_n}, \tilde{l}\colon G\langle \mathtt{A}|\tilde{\mathtt{B}}|\tilde{\mathtt{B}}\rangle$ (as of Lemma 7).

Finally, by the definition of inclusion of service typings in Γ (cf Section 1.2.1), we can write judgement $\tilde{l}\colon G\langle \mathtt{A}|\tilde{\mathtt{B}}|\tilde{\mathtt{B}}\rangle$ as the sequence of judgements $\tilde{l}\colon G\langle \mathtt{A}|\tilde{\mathtt{B}}|\varnothing\rangle, \tilde{l}\colon G\langle \mathtt{A}|\tilde{\mathtt{B}}|\mathtt{B}_1\rangle, \ldots, \tilde{l}\colon G\langle \mathtt{A}|\tilde{\mathtt{B}}|\mathtt{B}_n\rangle$.

Therefore we write $[\![\Gamma']\!]^C$ as

$$[\![\Gamma']\!]_C = \Gamma'', \Gamma''_{\mathsf{p}}, \Gamma''_{\mathsf{q}_1}, \ldots, \Gamma''_{\mathsf{q}_n}, \tilde{l}\colon G\langle \mathtt{A}|\tilde{\mathtt{B}}|\varnothing\rangle, \tilde{l}\colon G\langle \mathtt{A}|\tilde{\mathtt{B}}|\mathtt{B}_1\rangle, \ldots, \tilde{l}\colon G\langle \mathtt{A}|\tilde{\mathtt{B}}|\mathtt{B}_n\rangle$$

Let $\widetilde{l.\mathsf{q}[\mathtt{B}]}\Big|_i = \{l_i.\mathsf{q}_i[\mathtt{B}_i], \ldots, l_n.\mathsf{q}_n[\mathtt{B}_n]\}$, we prove the case by proving the typing derivation for $[\![\Gamma]\!]^C \vdash_{\mathtt{min}} [\![C]\!]$.

From the definition of EPP (Definition 5 of the article) we can write

$$[\![C]\!] \equiv C_s \mid C''$$

where, given the shape of C, we know that C'' is the same as the one generated from $[\![C']\!]$, as seen above. C_s is

$$C_s = \mathsf{req}\ k : \mathsf{p}[\mathtt{A}] \Leftrightarrow \widetilde{l.\mathtt{B}}; [\![C']\!]_{\mathsf{p}} \quad \Big| \prod_{l.\mathsf{r}[\mathtt{C}]\ \in\ \{\widetilde{l.\mathsf{q}[\mathtt{B}]}\}} \mathsf{acc}\ k : l.\mathsf{r}[\mathtt{C}]; [\![C']\!]_{\mathsf{r}}$$

We now prove we can derive the typing of $[\![\Gamma]\!]^C \vdash_{\mathtt{min}} [\![C]\!]$

$$\dfrac{\Gamma'' \vdash_{\mathtt{min}} C'' \qquad \dfrac{\dfrac{\Gamma''_{\mathsf{p}}, \mathsf{p}\colon k[\mathtt{A}], k[\mathtt{A}]\colon [\![G]\!]_{\mathtt{A}} \vdash_{\mathtt{min}} [\![C']\!]_{\mathsf{p}} \qquad \tilde{l} \notin \Gamma''_{\mathsf{p}}}{\Gamma''_{\mathsf{p}}, \tilde{l}\colon G\langle \mathtt{A}|\tilde{\mathtt{B}}|\varnothing\rangle \vdash_{\mathtt{min}} \mathsf{req}\ k : \mathsf{p}[\mathtt{A}] \Leftrightarrow \widetilde{l.\mathtt{B}}; [\![C']\!]_{\mathsf{p}}}\lfloor^{\mathsf{Min}}|_{\mathsf{Req1}}\rceil \qquad \Delta_1}{\Gamma''_{\mathsf{p}}, \tilde{l}\colon G\langle \mathtt{A}|\tilde{\mathtt{B}}|\varnothing\rangle, \Gamma''_{\mathsf{q}_1}, \tilde{l}\colon G\langle \mathtt{A}|\tilde{\mathtt{B}}|\mathtt{B}_1\rangle, \ldots, \Gamma''_{\mathsf{q}_n}, \tilde{l}\colon G\langle \mathtt{A}|\tilde{\mathtt{B}}|\mathtt{B}_n\rangle \vdash_{\mathtt{min}} C_s}\lfloor^{\mathsf{Min}}|_{\mathsf{Par}}\rceil}{\Gamma'', \Gamma''_{\mathsf{p}}, \tilde{l}\colon G\langle \mathtt{A}|\tilde{\mathtt{B}}|\varnothing\rangle, \Gamma''_{\mathsf{q}_1}, \tilde{l}\colon G\langle \mathtt{A}|\tilde{\mathtt{B}}|\mathtt{B}_1\rangle, \ldots, \Gamma''_{\mathsf{q}_n}, \tilde{l}\colon G\langle \mathtt{A}|\tilde{\mathtt{B}}|\mathtt{B}_n\rangle \vdash_{\mathtt{min}} C_s \mid C''}\lfloor^{\mathsf{Min}}|_{\mathsf{Par}}\rceil$$

where

$$\Delta_i = \dfrac{\Delta_{i+1} \qquad \dfrac{l_i \subseteq \tilde{l} \qquad \Gamma''_{\mathsf{q}_i}, \mathbf{init}(\mathsf{q}_i[\mathtt{B}_i], k, G) \vdash_{\mathtt{min}} [\![C']\!]_{\mathsf{q}_i} \qquad \mathsf{q}_i \notin \Gamma''_{\mathsf{q}_i} \qquad \tilde{l} \notin \Gamma''_{\mathsf{q}_i}}{\Gamma''_{\mathsf{q}_i}, \tilde{l}\colon G\langle \mathtt{A}|\tilde{\mathtt{B}}|\mathtt{B}_i\rangle \vdash_{\mathtt{min}} \mathsf{acc}\ k : l_i.\mathsf{q}_i[\mathtt{B}_i]; [\![C']\!]_{\mathsf{q}_i}}\lfloor^{\mathsf{Min}}|_{\mathsf{Acc}}\rceil}{\Gamma''_{\mathsf{q}_i}, \tilde{l}\colon G\langle \mathtt{A}|\tilde{\mathtt{B}}|\mathtt{B}_i\rangle, \ldots, \Gamma''_{\mathsf{q}_n}, \tilde{l}\colon G\langle \mathtt{A}|\tilde{\mathtt{B}}|\mathtt{B}_n\rangle \vdash_{\mathtt{min}} \mathsf{acc}\ k : l_i.\mathsf{q}_i[\mathtt{B}_i]; [\![C']\!]_{\mathsf{q}_i} \mid \prod_{l.\mathsf{r}[\mathtt{C}]\ \in\ \widetilde{l.\mathsf{q}[\mathtt{B}]}|_{i+1}} \mathsf{acc}\ k : l.\mathsf{r}[\mathtt{C}]; [\![C']\!]_{\mathsf{r}}}\lfloor^{\mathsf{Min}}|_{\mathsf{Par}}\rceil$$

Note that we are reporting only the derivation terminating with $\lfloor^{\mathsf{Min}}|_{\mathsf{Req1}}\rceil$, i.e., the one that applies when Γ''_{p} does not contain the typing of $\tilde{l}$. The other case is similar and it applies rule $\lfloor^{\mathsf{Min}}|_{\mathsf{Req2}}\rceil$.

- $\Gamma'' \vdash_{\mathtt{min}} C''$;
- $\Gamma''_{\mathsf{p}}, \mathsf{p}\colon k[\mathtt{A}], k[\mathtt{A}]\colon [\![G]\!]_{\mathtt{A}} \vdash_{\mathtt{min}} [\![C']\!]_{\mathsf{p}}$;
- $\Gamma''_{\mathsf{q}_i}, \mathbf{init}(\mathsf{q}_i[\mathtt{B}_i], k, G) \vdash_{\mathtt{min}} [\![C']\!]_{\mathsf{q}_i}$.

hold by the induction hypothesis.

Case $\lfloor^{\mathsf{Min}}|_{\mathsf{Start2}}\rceil$
Similar to case $\lfloor^{\mathsf{Min}}|_{\mathsf{Start1}}\rceil$.

Case $\lfloor^{\mathsf{Min}}|_{\mathsf{Req1}}\rceil$
and ***Case.*** $\lfloor^{\mathsf{Min}}|_{\mathsf{Req2}}\rceil$ follow the proof of case $\lfloor^{\mathsf{Min}}|_{\mathsf{Start1}}\rceil$, focussing on the request branch.

Case $\lfloor^{\mathsf{Min}}|_{\mathsf{Acc}}\rceil$
Follows the proof of case $\lfloor^{\mathsf{Min}}|_{\mathsf{Start1}}\rceil$, following the accept branch.

Case $\lfloor^{\mathsf{Min}}|_{\mathsf{Cond}}\rceil$
By induction hypothesis on C_1 or C_2.

Case $\lfloor^{\mathsf{Min}}|_{\mathsf{Com}}\rceil$
From the premises we have $C = k\colon \mathsf{p}[\mathtt{A}].e \longrightarrow\!\!\triangleright\ \mathsf{q}[\mathtt{B}].o(x); C'$ on which we can apply the typing derivation

$$\dfrac{\Gamma' \vdash \mathsf{p}\colon k[\mathtt{A}], \mathsf{q}\colon k[\mathtt{B}] \qquad \Gamma' \vdash \mathsf{p}.e\colon U \qquad \Gamma', \mathsf{q}.x\colon U, k[\mathtt{A}]\colon T, k[\mathtt{B}]\colon T' \vdash_{\mathtt{min}} C'}{\Gamma', k[\mathtt{A}]\colon \oplus\, \mathtt{B}.o(U); T, k[\mathtt{B}]\colon \&\mathtt{A}.o(U); T' \vdash_{\mathtt{min}} k\colon \mathsf{p}[\mathtt{A}].e \longrightarrow\!\!\triangleright\ \mathsf{q}[\mathtt{B}].o(x); C'}\ \lfloor^{\mathsf{Min}}|_{\mathsf{Com}}\rceil$$

Hence we consider $\Gamma = \Gamma', k[\mathtt{A}]\colon \oplus\, \mathtt{B}.o(U); T, k[\mathtt{B}]\colon \&\mathtt{A}.o(U); T'$. From the definition of EPP (Definition 5 of the article) we have $[\![C]\!] \equiv C_c \mid C''$ where

$$C_c = k\colon \mathsf{p}[\mathtt{A}].e \longrightarrow\!\!\triangleright\ \mathtt{B}.o; [\![C']\!]_{\mathsf{p}} \ \mid\ k\colon \mathtt{A} \longrightarrow\!\!\triangleright\ \mathsf{q}[\mathtt{B}].o(x); [\![C']\!]_{\mathsf{q}}$$

$$C'' = \prod_{\mathsf{r}\, \in\, \{\mathbf{fp}(C') \setminus \{\mathsf{p},\mathsf{q}\}\}} [\![C']\!]_{\mathsf{r}} \ \mid\ \prod_{l} \left(\bigsqcup_{\mathsf{s}\, \in\, \lfloor C' \rfloor_l} [\![C']\!]_{\mathsf{s}} \right)$$

From the definition of $[\![\Gamma]\!]^C$ we can write

$$[\![\Gamma]\!]^C = [\![\Gamma']\!]^C, k[\mathtt{A}]\colon \oplus\, \mathtt{B}.o(U); T, k[\mathtt{A}]\colon \&\mathtt{A}.o(U); T'$$

from the induction hypothesis we have that, let $\Gamma_c = \Gamma', \mathsf{q}.x\colon U, k[\mathtt{A}]\colon T, k[\mathtt{B}]\colon T'$, $\Gamma_c \vdash_{\mathtt{min}} C'$ and therefore $[\![\Gamma_c]\!]^{C'} \vdash_{\mathtt{min}} [\![C']\!]$. We can partition $[\![\Gamma_c]\!]^{C'}$ as

$$[\![\Gamma_c]\!]^{C'} = \Gamma'', \Gamma_{\mathsf{p}}, k[\mathtt{A}]\colon T, \Gamma_{\mathsf{q}}, \mathsf{q}.x\colon U, k[\mathtt{B}]\colon T'$$

such that

$$\dfrac{\Gamma'' \vdash_{\mathtt{min}} C'' \qquad \dfrac{\Gamma_{\mathsf{p}}, k[\mathtt{A}]\colon T \vdash_{\mathtt{min}} [\![C']\!]_{\mathsf{p}} \qquad \Gamma_{\mathsf{q}}, \mathsf{q}.x\colon U, k[\mathtt{B}]\colon T' \vdash_{\mathtt{min}} [\![C']\!]_{\mathsf{q}}}{\Gamma_{\mathsf{p}}, k[\mathtt{A}]\colon T, \Gamma_{\mathsf{q}}, \mathsf{q}.x\colon U, k[\mathtt{B}]\colon T' \vdash_{\mathtt{min}} [\![C']\!]_{\mathsf{p}} \mid [\![C']\!]_{\mathsf{q}}}\ \lfloor^{\mathsf{Min}}|_{\mathsf{Par}}\rceil}{\Gamma'', \Gamma_{\mathsf{p}}, k[\mathtt{A}]\colon T, \Gamma_{\mathsf{q}}, \mathsf{q}.x\colon U, k[\mathtt{B}]\colon T' \vdash_{\mathtt{min}} [\![C']\!]_{\mathsf{p}} \mid [\![C']\!]_{\mathsf{q}} \mid C''}\ \lfloor^{\mathsf{Min}}|_{\mathsf{Par}}\rceil$$

From the derivation on rule $\lfloor^{\mathsf{Min}}|_{\mathsf{Com}}\rceil$ we know that

$$[\![\Gamma']\!]^{C'} = \Gamma'', \Gamma_{\mathsf{p}}, \Gamma_{\mathsf{q}}$$

and therefore that

$$[\![\Gamma]\!]^C = \Gamma'', \Gamma_{\mathsf{p}}, k[\mathtt{A}]\colon \oplus\, \mathtt{B}.o(U); T, \Gamma_{\mathsf{q}}, k[\mathtt{B}]\colon \oplus\, \mathtt{A}.o(U); T'$$

To prove $[\![\Gamma]\!]^C \vdash_{\mathtt{min}} [\![C]\!]$ we prove that we can apply rule $\lfloor^{\mathsf{Min}}|_{\mathsf{Par}}\rceil$.

$$\dfrac{\Gamma'' \vdash_{\mathtt{min}} C'' \qquad \dfrac{\dfrac{\begin{array}{c}\Gamma_{\mathsf{p}} \vdash \mathsf{p}\colon k[\mathtt{A}] \quad \mathsf{q}\colon k[\mathtt{B}] \notin \Gamma_{\mathsf{p}} \\ \Gamma_{\mathsf{p}} \vdash \mathsf{p}.e\colon U \\ \Gamma_{\mathsf{p}}, k[\mathtt{A}]\colon T \vdash_{\mathtt{min}} [\![C']\!]_{\mathsf{p}}\end{array}}{\begin{array}{l}\Gamma_{\mathsf{p}}, k[\mathtt{A}]\colon \oplus\, \mathtt{B}.o(U); T \\ \qquad \vdash_{\mathtt{min}} k\colon \mathsf{p}[\mathtt{A}].e \longrightarrow\!\!\triangleright\ \mathtt{B}.o; [\![C']\!]_{\mathsf{p}}\end{array}}\ \lfloor^{\mathsf{Min}}|_{\mathsf{Send}}\rceil \qquad \dfrac{\begin{array}{c}\Gamma_{\mathsf{q}} \vdash \mathsf{p}\colon k[\mathtt{B}] \qquad \mathsf{p}\colon k[\mathtt{A}] \notin \Gamma_{\mathsf{q}} \\ \Gamma_{\mathsf{q}}, \mathsf{q}.x\colon U, k[\mathtt{B}]\colon T' \vdash_{\mathtt{min}} [\![C']\!]_{\mathsf{q}}\end{array}}{\begin{array}{l}\Gamma_{\mathsf{p}}, k[\mathtt{B}]\colon \&\mathtt{A}.o(U); T' \\ \qquad \vdash_{\mathtt{min}} k\colon \mathtt{A} \longrightarrow\!\!\triangleright\ \mathsf{q}[\mathtt{B}].o(x); [\![C']\!]_{\mathsf{p}}\end{array}}\ \lfloor^{\mathsf{Min}}|_{\mathsf{Recv}}\rceil}{\begin{array}{l}\Gamma_{\mathsf{p}}, k[\mathtt{A}]\colon \oplus\, \mathtt{B}.o(U); T, \Gamma_{\mathsf{q}}, k[\mathtt{B}]\colon \oplus\, \mathtt{A}.o(U); T' \\ \qquad \vdash_{\mathtt{min}} k\colon \mathsf{p}[\mathtt{A}].e \longrightarrow\!\!\triangleright\ \mathtt{B}.o; [\![C']\!]_{\mathsf{p}} \mid k\colon \mathtt{A} \longrightarrow\!\!\triangleright\ \mathsf{q}[\mathtt{B}].o(x); [\![C']\!]_{\mathsf{q}}\end{array}}\ \lfloor^{\mathsf{Min}}|_{\mathsf{Par}}\rceil}{\begin{array}{l}\Gamma'', \Gamma_{\mathsf{p}}, k[\mathtt{A}]\colon \oplus\, \mathtt{B}.o(U); T, \Gamma_{\mathsf{q}}, k[\mathtt{B}]\colon \oplus\, \mathtt{A}.o(U); T' \\ \qquad \vdash_{\mathtt{min}} k\colon \mathsf{p}[\mathtt{A}].e \longrightarrow\!\!\triangleright\ \mathtt{B}.o; [\![C']\!]_{\mathsf{p}} \mid k\colon \mathtt{A} \longrightarrow\!\!\triangleright\ \mathsf{q}[\mathtt{B}].o(x); [\![C']\!]_{\mathsf{q}} \mid C''\end{array}}\ \lfloor^{\mathsf{Min}}|_{\mathsf{Par}}\rceil$$

Case $\lfloor^{\mathsf{Min}}|_{\mathsf{Send}}\rceil$
Analogous to case $\lfloor^{\mathsf{Min}}|_{\mathsf{Com}}\rceil$

Case $\lfloor^{\mathsf{Min}}|_{\mathsf{Recv}}\rceil$
Analogous to case $\lfloor^{\mathsf{Min}}|_{\mathsf{Com}}\rceil$.

Case $\lfloor^{\mathsf{Min}}|_{\mathsf{Par}}\rceil$
From the premises we know that $C = C_1 \mid C_2$ on which we can apply the typing derivation

$$\frac{\Gamma_1 \vdash_{\mathtt{min}} C_1 \qquad \Gamma_2 \vdash_{\mathtt{min}} C_2}{\Gamma_1, \Gamma_2 \vdash_{\mathtt{min}} C_1 \mid C_2} \lfloor^{\mathsf{Min}}|_{\mathsf{Par}}\rceil$$

the case is proved applying the induction hypothesis.

Case $\lfloor^{\mathsf{Min}}|_{\mathsf{Def}}\rceil$
From the premises we know that $C = \mathtt{def}\ X = C''\ \mathtt{in}\ C'$ on which we can apply the typing derivation, with $\Gamma = (\Gamma' \triangledown \Gamma''), \mathsf{solve}(\widetilde{k[\mathtt{A}] : T} \triangledown \widetilde{k'[\mathtt{A}'] : T'}, \mathbf{t}_X)$

$$\frac{\begin{array}{c}\Gamma_x(X) = \Gamma'_x(X) \text{ if } X \in \mathbf{dom}(\Gamma_x) \cap \mathbf{dom}(\Gamma'_x) \qquad \nexists\, k''[\mathtt{A}''] \in \mathbf{dom}(\Gamma \triangledown \Gamma') \\ X \notin \mathbf{dom}(\Gamma \triangledown \Gamma') \quad \Gamma'_x \rhd_X (\Gamma'', \widetilde{k'[\mathtt{A}'] : T'}), C'' \qquad \Gamma_x \rhd_X (\Gamma', \widetilde{k[\mathtt{A}] : T}), C' \qquad \Gamma'|_{\mathbf{locs}} \subseteq \Gamma'\end{array}}{(\Gamma' \triangledown \Gamma''), \mathsf{solve}(\widetilde{k[\mathtt{A}] : T} \triangledown \widetilde{k'[\mathtt{A}'] : T'}, \mathbf{t}_X) \vdash_{\mathtt{min}} \mathtt{def}\ X = C''\ \mathtt{in}\ C'} \lfloor^{\mathsf{Min}}|_{\mathsf{Def}}\rceil$$

To prove $[\![\Gamma]\!]^C \vdash_{\mathtt{min}} [\![C]\!]$, we consider the processes $\mathsf{p} \in \tilde{\mathsf{p}} = \mathbf{pn}([\![C]\!])$ with cardinality $[1, n]$ and we let

- $[\![C]\!] = \prod_{\mathsf{p}} C_{\mathsf{p}}$
- $C_{\mathsf{p}} = \mathtt{def}\ X_{\mathsf{p}} = [\![C''[X_{\mathsf{p}}/X]]\!]_{\mathsf{p}}\ \mathtt{in}\ [\![C'[X_{\mathsf{p}}/X]]\!]_{\mathsf{p}}$
- $\Gamma_c = [\![\Gamma]\!]^C$
- $\widetilde{k_{\mathsf{p}}[\mathtt{A}] : T} = \left\{ k[\mathtt{A}] : T \mid \{\mathsf{p} : k[\mathtt{A}], k[\mathtt{A}] : T\} \subseteq \Gamma_c \wedge k[\mathtt{A}] : T \in \widetilde{k[\mathtt{A}] : T} \right\}$
- $\widetilde{k'_{\mathsf{p}}[\mathtt{A}'] : T'} = \left\{ k'[\mathtt{A}'] : T' \mid \{\mathsf{p} : k'[\mathtt{A}'], k'[\mathtt{A}'] : T'\} \subseteq \Gamma_c \wedge k'[\mathtt{A}'] : T' \in \widetilde{k'[\mathtt{A}'] : T'} \right\}$

The case is proved by the derivation Δ_1 where

$$\Delta_i = \frac{\pi_{\mathsf{p}_i} \qquad \dfrac{\Delta_{i+1}}{\bigcup_{\mathsf{p} \in \{\mathsf{p}_{i+1}, \ldots, \mathsf{p}_n\}} \Gamma_c|_{\mathsf{p}} \vdash_{\mathtt{min}} \prod_{\mathsf{p} \in \{\mathsf{p}_{i+1}, \ldots, \mathsf{p}_n\}} C_{\mathsf{p}}} \lfloor^{\mathsf{Min}}|_{\mathsf{Par}}\rceil}{\Gamma_c|_{\mathsf{p}_i}, \bigcup_{\mathsf{p} \in \{\mathsf{p}_{i+1}, \ldots, \mathsf{p}_n\}} \Gamma_c|_{\mathsf{p}} \vdash_{\mathtt{min}} C_{\mathsf{p}_i} \mid \prod_{\mathsf{p} \in \{\mathsf{p}_{i+1}, \ldots, \mathsf{p}_n\}} C_{\mathsf{p}}} \lfloor^{\mathsf{Min}}|_{\mathsf{Par}}\rceil$$

and

$$\pi_{\mathsf{p}} = \frac{\begin{array}{c}\Gamma_x(X_{\mathsf{p}}) = \Gamma'_x(X_{\mathsf{p}}) \text{ if } X_{\mathsf{p}} \in \mathbf{dom}(\Gamma_x) \cap \mathbf{dom}(\Gamma'_x) \\ \nexists k''[\mathtt{A}'']: \in \mathbf{dom}\left([\![\Gamma']\!]^C\Big|_{\mathsf{p}} \triangledown [\![\Gamma'']\!]^C\Big|_{\mathsf{p}} \right) \qquad \Gamma'_x \rhd \left([\![\Gamma'']\!]^C\Big|_{\mathsf{p}}, \widetilde{k'_{\mathsf{p}}[\mathtt{A}'] : T'} \right), [\![C''[X_{\mathsf{p}}/X]]\!]_{\mathsf{p}} \\ \Gamma_x \rhd \left([\![\Gamma']\!]^C\Big|_{\mathsf{p}}, \widetilde{k_{\mathsf{p}}[\mathtt{A}] : T} \right), [\![C''[X_{\mathsf{p}}/X]]\!]_{\mathsf{p}} \qquad [\![\Gamma'']\!]^C\Big|_{\mathsf{p}}\Big|_{\mathsf{locs}} \subseteq [\![\Gamma']\!]^C\Big|_{\mathsf{p}}\end{array}}{[\![\Gamma']\!]^C\Big|_{\mathsf{p}} \triangledown [\![\Gamma'']\!]^C\Big|_{\mathsf{p}}, \mathsf{solve}\left(\widetilde{k_{\mathsf{p}}[\mathtt{A}] : T} \triangledown \widetilde{k'_{\mathsf{p}}[\mathtt{A}'] : T'}, \mathbf{t}_{X_{\mathsf{p}}} \right) \vdash_{\mathtt{min}} \mathtt{def}\ X_{\mathsf{p}} = [\![C''[X_{\mathsf{p}}/X]]\!]_{\mathsf{p}}\ \mathtt{in}\ [\![C'[X_{\mathsf{p}}/X]]\!]_{\mathsf{p}}} \lfloor^{\mathsf{Min}}|_{\mathsf{Def}}\rceil$$

Essentially, using the filtrations $\Gamma|_{\mathsf{p}}$ and the partitions $\widetilde{k_{\mathsf{p}}[\mathtt{A}] : T}$ and $\widetilde{k'_{\mathsf{p}}[\mathtt{A}'] : T'}$ in Δ_i, we shape $[\![\Gamma]\!]^C$ in such a way that its partitions contain all and only the typings (variable, ownership, definitions)

that minimally type the endpoint choreography C_{p}, with the exception of service typings, which are duplicated in all filtrations (as per its definition). However, this is not a problem, as we consider a weakened form of minimal typing that allows for additional, unused service typings.

Such a partitioning of $[\![\Gamma]\!]^C$ is possible by the definitions of $[\![\Gamma]\!]^C$ and $\triangledown$ (and $\prec$ by extension):

$$\begin{aligned}
[\![\Gamma]\!]^C &= \left[\!\!\left[(\Gamma' \triangledown \Gamma''), \mathsf{solve}(\widetilde{k[\mathtt{A}] : T} \triangledown \widetilde{k'[\mathtt{A}'] : T'}, \mathbf{t}_X)\right]\!\!\right]^C = \\
&= \bigcup_{\mathsf{p} \in \tilde{\mathsf{p}}} \left(\left([\![\Gamma']\!]^C \triangledown [\![\Gamma'']\!]^C \right), \left[\!\!\left[\mathsf{solve}(\widetilde{k[\mathtt{A}] : T} \triangledown \widetilde{k'[\mathtt{A}'] : T'}, \mathbf{t}_X)\right]\!\!\right]^C \right)\Big|_{\mathsf{p}} = \\
&= \bigcup_{\mathsf{p} \in \tilde{\mathsf{p}}} \left(\left([\![\Gamma']\!]^C \triangledown [\![\Gamma'']\!]^C \right), \mathsf{solve}(\widetilde{k[\mathtt{A}] : T} \triangledown \widetilde{k'[\mathtt{A}'] : T'}, \mathbf{t}_X) \right)\Big|_{\mathsf{p}}
\end{aligned}$$

Finally, we simply rename $\mathbf{t}_X$ to $\mathbf{t}_{X_{\mathsf{p}}}$ (in each filtration $\mathsf{p} \in \tilde{\mathsf{p}}$).

Then in π_{p} we prove the partition $\Gamma_c|_{\mathsf{p}}$ to minimally type the endpoint choreography C_{p}. All preconditions in π_{p} hold as the environments $[\![\Gamma']\!]^C$, $[\![\Gamma'']\!]^C$, $\widetilde{k_{\mathsf{p}}[\mathtt{A}] : T}$, and $\widetilde{k'_{\mathsf{p}}[\mathtt{A}'] : T'}$, contain those and only definition, ownership, variable, and session types related to process p (with the exception of duplicated service typings) and originally contained in Γ', Γ'',$\widetilde{k[\mathtt{A}] : T}$, and $\widetilde{k'[\mathtt{A}'] : T'}$. Definition typing identifiers are properly renamed to be unique for p (i.e., from X to X_{p}).

Case $\lfloor^{\mathsf{Min}}|_{\mathsf{End}}\rceil$

Trivial.

Case $\lfloor^{\mathsf{Min}}|_{\mathsf{Call}}\rceil$

From the premises we know that $C = X$, on which we can apply the typing derivation

$$\frac{\begin{array}{c}\Gamma' = \mathsf{vars} \cup \mathsf{ownerships} \qquad \widetilde{k'[\mathtt{A}']} = \mathsf{sessions} \setminus \{\widetilde{k[\mathtt{A}]}\} \\ \Gamma'' = \mathsf{vars}(X) \cup \mathsf{ownerships}(X) \quad \widetilde{k[\mathtt{A}]} = \mathsf{sessions}(\mathsf{X}) \qquad \Gamma'' \subseteq \Gamma'\end{array}}{\Gamma', \widetilde{k[\mathtt{A}]} : \mathbf{t}_X, \widetilde{k'[\mathtt{A}']} : \mathsf{end}, X \colon (\Gamma'', \widetilde{k[\mathtt{A}]} : \mathbf{t}_X) \vdash_{\mathtt{min}} X} \lfloor^{\mathsf{Min}}|_{\mathsf{Call}}\rceil$$

Thus, in the case, $\Gamma = \Gamma', \widetilde{k[\mathtt{A}]} : \mathbf{t}_X, \widetilde{k'[\mathtt{A}']} : \mathsf{end}, X \colon (\Gamma'', \widetilde{k[\mathtt{A}]} : \mathbf{t}_X)$. Given our assumption of well sortedness, we can consider as EPP of X the composition

$$[\![X]\!] = \prod_{\mathsf{p} \in \tilde{\mathsf{p}}} X_{\mathsf{p}}$$

Where processes $\tilde{\mathsf{p}}$ are a subset of the processes present both in the prefix of procedure call X in C and in the typing environment Γ (we recall, Γ contains typings that are coalesced in $[\![\Gamma]\!]^C$). From the definition of $[\![\Gamma]\!]^C$, we can write

$$\Gamma_c = [\![\Gamma]\!]^C = [\![\Gamma']\!]^C, \widetilde{k_{\mathsf{p}}[\mathtt{A}]} : \mathbf{t}_X, \widetilde{k'_{\mathsf{p}}[\mathtt{A}']} : \mathsf{end}, \bigcup_{\mathsf{p} \in \tilde{\mathsf{p}}} X_{\mathsf{p}} \colon ([\![\Gamma'']\!]_{\mathsf{p}}, \widetilde{k_{\mathsf{p}}[\mathtt{A}]} : \mathbf{t}_{X_{\mathsf{p}}})$$

where

- $\widetilde{k_{\mathsf{p}}[\mathtt{A}]} \colon \mathbf{t}_{X_{\mathsf{p}}} = \left\{ k_{\mathsf{p}}[\mathtt{A}] \colon \mathbf{t}_{X_{\mathsf{p}}} \mid \{\mathsf{p} \colon k[\mathtt{A}], k[\mathtt{A}] \colon \mathbf{t}_X\} \subseteq \Gamma \right\}$
- $\widetilde{k'_{\mathsf{p}}[\mathtt{A}']} \colon \mathsf{end} = \left\{ k'_{\mathsf{p}}[\mathtt{A}'] \colon \mathsf{end} \mid \{\mathsf{p} \colon k'[\mathtt{A}'], k'[\mathtt{A}'] \colon \mathsf{end}\} \subseteq \Gamma \right\}$

Finally, let the cardinality of $\tilde{\mathsf{p}}$ be $[1, n]$. The case is proved by the derivation Δ_1 where

$$\Delta_i = \cfrac{\pi_{\mathsf{p}_i} \quad \cfrac{\Delta_{i+1}}{\bigcup_{\mathsf{p} \in \{\mathsf{p}_{i+1},\ldots,\mathsf{p}_n\}} \Gamma_c|_{\mathsf{p}} \vdash_{\mathtt{min}} \prod_{\mathsf{p}\in\{\mathsf{p}_{i+1},\ldots,\mathsf{p}_n\}} X_{\mathsf{p}}} \lfloor^{\mathsf{Min}}|_{\mathsf{Par}}\rceil}{\Gamma_c|_{\mathsf{p}_i}, \bigcup_{\mathsf{p} \in \{\mathsf{p}_{i+1},\ldots,\mathsf{p}_n\}} \Gamma_c|_{\mathsf{p}} \vdash_{\mathtt{min}} X_{\mathsf{p}_i} \mid \prod_{\mathsf{p}\in\{\mathsf{p}_{i+1},\ldots,\mathsf{p}_n\}} X_{\mathsf{p}}} \lfloor^{\mathsf{Min}}|_{\mathsf{Par}}\rceil$$

and

$$\pi_{\mathsf{p}} = \cfrac{\begin{array}{c}[\![\Gamma']\!]^C\Big|_{\mathsf{p}} = \mathsf{vars} \cup \mathsf{ownerships} \qquad \widetilde{k'_{\mathsf{p}}[\mathtt{A}']} = \mathsf{sessions} \setminus \{\widetilde{k_{\mathsf{p}}[\mathtt{A}]}\} \\ [\![\Gamma']\!]^C\Big|_{\mathsf{p}} = \mathsf{vars}(X_{\mathsf{p}}) \cup \mathsf{ownerships}(X_{\mathsf{p}}) \quad \widetilde{k_{\mathsf{p}}[\mathtt{A}]} = \mathsf{sessions}(\mathsf{X}_{\mathsf{p}}) \quad [\![\Gamma'']\!]_{\mathsf{p}} \subseteq [\![\Gamma']\!]^C\Big|_{\mathsf{p}}\end{array}}{[\![\Gamma']\!]^C\Big|_{\mathsf{p}}, \widetilde{k_{\mathsf{p}}[\mathtt{A}]} : \mathbf{t}_{X_{\mathsf{p}}}, \widetilde{k'_{\mathsf{p}}[\mathtt{A}']} : \mathsf{end}, X_{\mathsf{p}} : ([\![\Gamma'']\!]_{\mathsf{p}}, \widetilde{k_{\mathsf{p}}[\mathtt{A}]} : \mathbf{t}_{X_{\mathsf{p}}}) \vdash_{\mathtt{min}} X_{\mathsf{p}}} \lfloor^{\mathsf{Min}}|_{\mathsf{Call}}\rceil$$

Where in π_p we consider the usage of auxiliary functions vars, owenerships, and sessions on the projection $[\![C]\!]_{\mathsf{p}}$.

□

We finally prove Theorem 5.

Proof of EPP Typing Preservation From Theorem 5, we have that $\Gamma = \Gamma_d, \Gamma_c$ and we need to prove that we can apply rule $\lfloor^{\mathsf{Min}}|_{\mathsf{DC}}\rceil$ on $\Gamma_d, [\![\Gamma_c]\!]^C \vdash_{\mathtt{min}} D, [\![C]\!]$

$$\cfrac{\mathbf{pco}(\Gamma_d, [\![\Gamma_c]\!]^C) \quad \Gamma_d \vdash D \quad [\![\Gamma_c]\!]^C \vdash_{\mathtt{min}} [\![C]\!]}{\Gamma_d, [\![\Gamma_c]\!]^C \vdash_{\mathtt{min}} D, [\![C]\!]} \lfloor^{\mathsf{Min}}|_{\mathsf{DC}}\rceil$$

where

- $\mathbf{pco}(\Gamma_d, [\![\Gamma]\!]^C)$ holds as, regarding session typings, $[\![\Gamma]\!]^C$ just coalesces session typings and their related ownerships of service processes;
- $\Gamma_d \vdash_{\mathtt{min}} D$ holds as per premises of Theorem 5;
- $[\![\Gamma]\!] \vdash_{\mathtt{min}} [\![C]\!]$ holds from Lemma 8 and the assumption of well-sortedness on C (if C is well-sorted also $[\![C]\!]$ is well-sorted and typable by $[\![\Gamma]\!]^C$).

□

3.6 EPP Theorem, continued

Before proving the two remaining properties of Theorem 2 of the article, we define some auxiliary concepts to establish a correspondence between a choreography and its projection.

Lemma 9 (EPP Swap Invariance)**.** Let $C \simeq_{\mathsf{C}} C'$ then $[\![C]\!] \simeq_{\mathsf{C}} [\![C']\!]$.

Proof Sketch In the proof, we show that the projection is invariant under the rules for the swapping relation $\simeq_{\mathsf{C}}$ defined in Figure 9 of the article. $\lfloor^{\mathsf{CS}}|_{\mathsf{EtaEta}}\rceil$ is trivial. For rule $\lfloor^{\mathsf{CS}}|_{\mathsf{EtaCnd}}\rceil$ we need to check that the projections of the processes in the swapped interaction η do not change, which holds by the definition of EPP for $(cond)$ terms and the merging operator (merging the same η returns η). The same reasoning on the EPP and the merging operator applies to all other cases. □

Lemma 10 (EPP under $\equiv$)**.** Let $C \equiv_{\mathsf{C}} C'$ then $[\![C]\!] \equiv_{\mathsf{C}} [\![C']\!]$.

Proof Easy by cases on the rules of $\equiv_{\mathsf{C}}$. □

Lemma 11 (Compositional EPP)**.** Let C be well-typed and $C = C_1 \mid C_2$ then $[\![C]\!] \equiv_{\mathsf{C}} [\![C_1]\!] \mid [\![C_2]\!]$.

Proof By definition of EPP

$$[\![C]\!] = \prod_{\mathsf{p} \in \mathbf{fp}(C)} [\![C]\!]_{\mathsf{p}} \mid \prod_{l} \left(\bigsqcup_{\mathsf{s} \in \lfloor C \rfloor_l} [\![C]\!]_{\mathsf{s}} \right)$$

Since C is well-typed and $C = C_1 \mid C_2$, rule $\lfloor^{\mathsf{T}}|_{\mathsf{Par}}\rceil$ applies and by definition of Γ_1, Γ_2 there cannot be a process p such that $\mathsf{p} \in \mathbf{fp}(C_1) \cap \mathbf{fp}(C_2)$. Therefore we can write

$$[\![C]\!] \equiv_{\mathsf{C}} \prod_{\mathsf{p} \in \mathbf{fp}(C_1)} [\![C_1]\!]_{\mathsf{p}} \mid \prod_{\mathsf{q} \in \mathbf{fp}(C_2)} [\![C_2]\!]_{\mathsf{q}} \mid \prod_{l} \left(\bigsqcup_{\mathsf{s} \in \lfloor C \rfloor_l} [\![C]\!]_{\mathsf{s}} \right)$$

By the definition of service typing we know that *i*) locations can implement only one role in a choreography and *ii*) a location can appear only in one service typing. Therefore there cannot be two service processes at the same location in C_1 and C_2. Thus we can write

$$[\![C]\!] \equiv_{\mathsf{C}} \underbrace{\prod_{\mathsf{p} \in \mathbf{fp}(C_1)} [\![C_1]\!]_{\mathsf{p}}}_{C_1^a} \mid \underbrace{\prod_{\mathsf{q} \in \mathbf{fp}(C_2)} [\![C_2]\!]_{\mathsf{q}}}_{C_2^a} \mid \underbrace{\prod_{l} \left(\bigsqcup_{\mathsf{r} \in \lfloor C_1 \rfloor_l} [\![C_1]\!]_{\mathsf{r}} \right)}_{C_1^s} \mid \underbrace{\prod_{l'} \left(\bigsqcup_{\mathsf{s} \in \lfloor C_2 \rfloor_{l'}} [\![C_2]\!]_{\mathsf{s}} \right)}_{C_2^s}$$

where $[\![C_1]\!] = C_1^a \mid C_1^s$ and $[\![C_2]\!] = C_2^a \mid C_2^s$ by definition of EPP. □

3.6.1 Pruning

Following our definition of EPP, the projection of $(start)$ terms on service processes yield a parallel composition of (acc) terms on the locations subject of the $(start)$. However, the reduction of a $(start)$ term might remove the availability to start new processes on the locations subject of the $(start)$ (i.e., if the reductum does not contain another $(start)$ term on the same locations). Contrarily, (acc) terms remain always available.

A similar observation can be drawn between conditional branches that contain (com) terms whose projection merges all possible communications into $(recv)$ and $(send)$ terms. Also in this case, reducing the condition and projecting the result we obtain a subset of all possible branches for the considered communication.

Similarly to Montesi and Yoshida (2013) and Carbone et al. (2012), we deal with these asymmetries by introducing the *pruning relation* (see Definition 6 of the article), which allows us to ignore unused *i*) endpoint services and *ii*) input branches.

Before continuing with the last auxiliary results and the proof of Theorem 2 of the article we need to extend the labels of the semantics of annotated Frontend Choreographies (see Section 3.1.1) with the identifiers of the processes involved in a reduction

$$\beta ::= k\colon \mathsf{p}[\mathtt{A}] \multimap \mathtt{B}.o \mid \mathtt{A}\rangle \mathsf{q}[\mathtt{B}].o(x) \mid \tau @\mathsf{p} \mid \tau$$

and the annotation of the reduction with rule $\lfloor^{\mathsf{C}}|_{\mathsf{Cond}}\rceil$ as

$$\frac{i = 1 \text{ if } \mathbf{eval}(e, D(\mathsf{p})) = \text{ true}, i = 2 \text{ otherwise}}{D,\ \mathtt{if}\ \mathsf{p}.e\ \{C_1\}\ \mathtt{else}\ \{C_2\} \ \xrightarrow{\tau @\mathsf{p}}\ D,\ C_i} \ \lfloor^{\mathsf{C}}|_{\mathsf{Cond}}\rceil$$

Let also $\mathbf{pn}(k\colon \mathsf{p}[\mathtt{A}] \multimap \mathtt{B}.o) = \{\mathsf{p}\}$, $\mathbf{pn}(\mathtt{A}\rangle \mathsf{q}[\mathtt{B}].o(x)) = \{\mathsf{q}\}$, $\mathbf{pn}(\tau @\mathsf{p}) = \{\mathsf{p}\}$, and $\mathbf{pn}(\tau) = \varnothing$

Lemma 12 (Passive Processes Pruning Invariance)**.** $D, C \xrightarrow{\beta} D', C'$ implies that for all $\mathsf{p} \in \mathbf{fp}(C) \setminus \mathbf{pn}(\beta)$, $[\![C']\!]_{\mathsf{p}} \prec [\![C]\!]_{\mathsf{p}}$.

Proof Sketch By cases on the derivation of C. The only interesting case is $\lfloor^{\mathsf{C}}|_{\mathsf{Cond}}\rceil$ in which the projection of the processes receiving selections are merged. The thesis follows directly from Definition 6 of the article and Lemmas 9 and 10.

□

3.7 Proof of Theorem 2 of the article

We restate items (2) and (3) of Theorem 2 of the article to include annotated reductions.

Theorem 2 of the article (EPP Operational Correspondence)
Let D, C be well-typed and well-annotated. Then,

1. *(Completeness) $D, C \xrightarrow{\beta} D', C'$ implies $D, [\![C]\!] \xrightarrow{\beta} D', C''$ and $[\![C']\!] \prec C''$.*
2. *(Soundness) $D, [\![C]\!] \xrightarrow{\beta} D', C''$ implies $D, C \xrightarrow{\beta} D', C'$ and $[\![C']\!] \prec C''$.*

We report below the respective proofs of *(Completeness)* and *(Soundness)* separately.

Proof (Completeness)

Proof by induction on the derivation of $D, C \xrightarrow{\beta} D', C'$.

Case $\lfloor^{\mathsf{C}}|_{\mathsf{Send}}\rceil$
we know that $C = k\colon \mathtt{p}[\mathtt{A}].e \longrightarrow\!\!\triangleright\, \mathtt{B}.o; C_c$ and we can write the derivation

$$\dfrac{\eta = k\colon \mathtt{p}[\mathtt{A}].e \longrightarrow\!\!\triangleright\, \mathtt{B}.o \qquad D, k\colon \mathtt{p}[\mathtt{A}].e \longrightarrow\!\!\triangleright\, \mathtt{B}.o \blacktriangleright D'}{D,\ \eta; C \xrightarrow{k\colon \mathtt{p}[\mathtt{A}] \longrightarrow\!\!\triangleright\, \mathtt{B}.o} D',\ C_c}\ \lfloor^{\mathsf{C}}|_{\mathsf{Send}}\rceil$$

and $C' = C_c$.

From the definition of EPP we have that $[\![C]\!] = C_{act} \mid C_s$ such that

$$C_{act} = k\colon \mathtt{p}[\mathtt{A}].e \longrightarrow\!\!\triangleright\, \mathtt{B}.o; [\![C_c]\!]_{\mathsf{p}} \mid \prod_{\mathsf{r}\, \in\, \mathbf{fp}(C) \setminus \{\mathsf{p}\}} [\![C_c]\!]_{\mathsf{r}}$$

and

$$C_s = \prod_{l} \left(\bigsqcup_{\mathsf{s}\, \in\, \lfloor C \rfloor_l} [\![C_c]\!]_{\mathsf{s}} \right)$$

While $[\![C']\!] \equiv_{\mathsf{C}} C'_{act} \mid C_s$

$$C'_{act} = [\![C_c]\!]_{\mathsf{p}} \mid \prod_{\mathsf{r}\, \in\, \mathbf{fp}(C') \setminus \{\mathsf{p}\}} [\![C_c]\!]_{\mathsf{r}}$$

We can apply Rules $\lfloor^{\mathsf{C}}|_{\mathsf{Par}}\rceil$, $\lfloor^{\mathsf{C}}|_{\mathsf{Eq}}\rceil$, and $\lfloor^{\mathsf{C}}|_{\mathsf{Send}}\rceil$ on $D, [\![C]\!]$ such that

$$\dfrac{\eta = k\colon \mathtt{p}[\mathtt{A}].e \longrightarrow\!\!\triangleright\, \mathtt{B}.o \qquad D, \eta \blacktriangleright D''}{D,\ [\![C]\!] \xrightarrow{k\colon \mathtt{p}[\mathtt{A}] \longrightarrow\!\!\triangleright\, \mathtt{B}.o} D'',\ C''}\ \vdots\ \lfloor^{\mathsf{C}}|_{\mathsf{Par}}\rceil$$

for which it holds that $D' = D''$ by rule $\lfloor^{\mathsf{D}}|_{\mathsf{Send}}\rceil$.

$$C'' = [\![C_c]\!]_{\mathsf{p}} \mid \prod_{\mathsf{r}\, \in\, \mathbf{fp}(C') \setminus \{\mathsf{p}\}} [\![C_c]\!]_{\mathsf{r}} \mid C_s$$

for which it holds that $[\![C']\!] \prec C''$.

Case $\lfloor^{\mathsf{C}}|_{\mathsf{Recv}}\rceil$

we know that $D, C = D,\ k\!:\!\mathtt{A} \multimap \mathsf{q}[\mathtt{B}].\{o_i(x_i);C_i\}_{i\in I}$ and we can write the derivation

$$\dfrac{j \in I \quad D, k\!:\!\mathtt{A} \multimap \mathsf{q}[\mathtt{B}].o_j(x_j) \blacktriangleright D'}{D,\ k\!:\!\mathtt{A} \multimap \mathsf{q}[\mathtt{B}].\{o_i(x_i);C_i\}_{i\in I} \xrightarrow{k:\mathtt{A}\rangle\mathsf{q}[\mathtt{B}].o_j(x_j)} D',\ C_j}\ \lfloor^{\mathsf{C}}|_{\mathsf{Recv}}\rceil$$

for $\beta = k : \mathtt{A}\rangle\mathsf{q}[\mathtt{B}].o_j(x_j)$ and $C' = C_j$.

By the definition of EPP we have

$$[\![C]\!] \equiv_{\mathsf{C}} k\!:\!\mathtt{A} \multimap \mathsf{q}[\mathtt{B}].\left\{o_i(x_i);[\![C_i]\!]_{\mathsf{q}}\right\}_{i\in I} \mid \prod_{\mathsf{p}\,\in\,\mathbf{fp}(C)\setminus\{\mathsf{q}\}} \left(\bigsqcup_{i\,\in\,I} [\![C_i]\!]_{\mathsf{p}}\right) \mid \prod_{l} \left(\bigsqcup_{\mathsf{r}\,\in\,\lfloor C\rfloor_l} [\![C]\!]_{\mathsf{r}}\right)$$

Then we can apply rules $\lfloor^{\mathsf{C}}|_{\mathsf{Par}}\rceil$, $\lfloor^{\mathsf{C}}|_{\mathsf{Eq}}\rceil$, and $\lfloor^{\mathsf{C}}|_{\mathsf{Recv}}\rceil$ such that

$$\dfrac{\dfrac{j \in I \quad D, k\!:\!\mathtt{A} \multimap \mathsf{q}[\mathtt{B}].o_j(x_j) \blacktriangleright D''}{\vdots\ \lfloor^{\mathsf{C}}|_{\mathsf{Par}}\rceil}}{D,\ [\![C]\!] \xrightarrow{k:\mathtt{A}\rangle\mathsf{q}[\mathtt{B}].o_j(x_j)} D'',\ [\![C_j]\!]_{\mathsf{q}} \mid \prod_{\mathsf{p}\,\in\,\mathbf{fp}(C)\setminus\{\mathsf{q}\}} \left(\bigsqcup_{i\,\in\,I} [\![C_i]\!]_{\mathsf{p}}\right) \mid \prod_{l} \left(\bigsqcup_{\mathsf{r}\,\in\,\lfloor C\rfloor_l} [\![C]\!]_{\mathsf{r}}\right)}$$

and

$$C'' = [\![C_j]\!]_{\mathsf{q}} \mid \prod_{\mathsf{p}\,\in\,\mathbf{fp}(C)\setminus\{\mathsf{q}\}} \left(\bigsqcup_{i\,\in\,I} [\![C_i]\!]_{\mathsf{p}}\right) \mid \prod_{l} \left(\bigsqcup_{\mathsf{r}\,\in\,\lfloor C\rfloor_l} [\![C]\!]_{\mathsf{r}}\right)$$

From rule $\lfloor^{\mathsf{D}}|_{\mathsf{Recv}}\rceil$ we know that $D'' = D'$. Finally $[\![C']\!] \prec C''$ by Definition 6 of the article and Lemma 12.

Case $\lfloor^{\mathsf{C}}|_{\mathsf{Start}}\rceil$

we know that $C = \mathtt{start}\ k : \mathsf{p}[\mathtt{A}] \Leftrightarrow \widetilde{l.\mathsf{q}[\mathtt{B}]};C_c$ and we can write the derivation

$$\dfrac{D\#k', \tilde{\mathsf{r}} \quad \delta = \mathtt{start}\ k' : \mathsf{p}[\mathtt{A}] \Leftrightarrow \widetilde{l.\mathsf{q}[\mathtt{B}]} \quad D,\delta \blacktriangleright D'}{D,\ \mathtt{start}\ k : \mathsf{p}[\mathtt{A}] \Leftrightarrow \widetilde{l.\mathsf{q}[\mathtt{B}]};C \quad \rightarrow \quad D',\ C[k'/k][\tilde{\mathsf{r}}/\tilde{\mathsf{q}}]}\ \lfloor^{\mathsf{C}}|_{\mathsf{Start}}\rceil$$

and $C' = C_c[k'/k][\tilde{\mathsf{r}}/\tilde{\mathsf{q}}]$.

From the definition of EPP we have

$$[\![C']\!] = \prod_{\mathsf{q}\,\in\,\mathbf{fp}(C')} [\![C']\!]_{\mathsf{q}} \mid \prod_{l} \left(\bigsqcup_{\mathsf{s}\,\in\,\lfloor C'\rfloor_l} [\![C']\!]_{\mathsf{s}}\right)$$

and

$$[\![C]\!] \equiv_{\mathsf{C}} \begin{cases} \quad \mathtt{req}\ k : \mathsf{p}[\mathtt{A}] \Leftrightarrow \widetilde{l.\mathtt{B}};[\![C_c]\!]_{\mathsf{p}} \\ \mid \displaystyle\prod_{l.\mathsf{q}[\mathtt{B}]\,\in\,\widetilde{l.\mathsf{q}[\mathtt{B}]}} \mathtt{acc}\ k : l.\mathsf{q}[\mathtt{B}];[\![C_c]\!]_{\mathsf{q}} \\ \mid \displaystyle\prod_{\mathsf{r}\,\in\,\mathbf{fp}(C)\setminus\{\mathsf{p}\}} [\![C]\!]_{\mathsf{r}} \\ \mid \displaystyle\prod_{l'\notin\tilde{l}} \left(\prod_{\mathsf{s}\,\in\,\lfloor C\rfloor_{l'}} [\![C]\!]_{\mathsf{s}}\right) \end{cases}$$

we can apply rules $\lfloor^{\mathsf{C}}|_{\mathsf{Par}}\rceil$, $\lfloor^{\mathsf{C}}|_{\mathsf{Eq}}\rceil$, $\lfloor^{\mathsf{C}}|_{\mathsf{PStart}}\rceil$ such that

$$\dfrac{\begin{array}{c} i \in \{1,\dots,n\} \qquad D\#k'',\tilde{\mathsf{r}}' \qquad \{\widetilde{l.\mathtt{B}}\} = \biguplus_i \{\widetilde{l_i.\mathtt{B}_i}\}_i \qquad \{\tilde{\mathsf{r}}'\} = \bigcup_i \{\tilde{\mathsf{r}}'_i\} \\ \delta = \mathtt{start}\ k'' : \mathsf{p}[\mathtt{A}] \twoheadleftarrow\!\!\twoheadrightarrow \widetilde{l_1.\mathsf{r}'_1[\mathtt{B}_1]},\dots,\widetilde{l_n.\mathsf{r}'_n[\mathtt{B}_n]} \qquad D,\delta \blacktriangleright D'' \\ \vdots\ \lfloor^{\mathsf{C}}|_{\mathsf{Par}}\rceil \end{array}}{D, [\![C]\!] \xrightarrow{\tau} D'',C''}$$

where

$$C'' \equiv_{\mathsf{C}} \begin{cases} [\![C_c]\!]_{\mathsf{p}}[k''/k] \\ |\ \prod_{(\mathsf{q},\mathsf{r}') \in \left\{(\mathsf{q}_1,\mathsf{r}'_1),\dots,(\mathsf{q}_n,\mathsf{r}'_n)\right\}} [\![C_c]\!]_{\mathsf{q}}[k''/k][\mathsf{q}/\mathsf{r}'] \\ |\ \prod_{\mathsf{r} \in \mathbf{fp}(C_c)\setminus\{\mathsf{p},\tilde{\mathsf{q}}\}} [\![C_c]\!]_{\mathsf{r}} \\ |\ \prod_{l.\mathsf{q}[\mathtt{B}] \in \widetilde{l.\mathsf{q}[\mathtt{B}]}} \mathsf{acc}\ k : l.\mathsf{q}[\mathtt{B}]; [\![C_c]\!]_{\mathsf{q}} \\ |\ \prod_{l' \notin \tilde{l}} \left(\prod_{\mathsf{s} \in \lfloor C_c \rfloor_{l'}} [\![C_c]\!]_{\mathsf{s}} \right) \end{cases}$$

Observe that we can α-rename k'' to k' and $\tilde{\mathsf{r}}'$ to $\tilde{\mathsf{r}}$ as k'', k', $\tilde{\mathsf{r}}'$, and $\tilde{\mathsf{r}}$ are all fresh wrt D,C. From the application of rule $\lfloor^{\mathsf{D}}|_{\mathsf{Start}}\rceil$ we can find Γ such that

$$\Gamma \vdash_{\mathtt{min}} (\ D'',C''\ )[k'/k''][\tilde{\mathsf{r}}/\tilde{\mathsf{r}}']$$

and

$$\Gamma \vdash_{\mathtt{min}} (D',C'')[k'/k''][\tilde{\mathsf{r}}/\tilde{\mathsf{r}}']$$

and by α-renaming we have that

$$D, [\![C]\!] \xrightarrow{\tau} D', C''[k'/k''][\tilde{\mathsf{r}}/\tilde{\mathsf{r}}']$$

Finally $[\![C']\!] \prec C''[k'/k''][\tilde{\mathsf{r}}/\tilde{\mathsf{r}}']$ by Lemma 12.

Case $\lfloor^{\mathsf{C}}|_{\mathsf{PStart}}\rceil$

Similar to (in particular the second part of) the proof of case $\lfloor^{\mathsf{C}}|_{\mathsf{Start}}\rceil$.

Case $\lfloor^{\mathsf{C}}|_{\mathsf{Cond}}\rceil$

we know that $C \equiv_{\mathsf{C}} \mathtt{if}\ \mathsf{p}.e\ \{C_1\}\ \mathtt{else}\ \{C_2\}$ and we can write the derivation

$$\dfrac{i = 1 \text{ if } \mathbf{eval}(e, D(\mathsf{p})) = \text{ true}, i = 2 \text{ otherwise}}{D,\ \mathtt{if}\ \mathsf{p}.e\ \{C_1\}\ \mathtt{else}\ \{C_2\} \quad \xrightarrow{\tau@\mathsf{p}} \quad D,\ C_i}\ \lfloor^{\mathsf{C}}|_{\mathsf{Cond}}\rceil$$

We only consider the case for $\mathbf{eval}(e, D(\mathsf{p})) = \text{true}$ as $\mathbf{eval}(e, D(\mathsf{p})) = \text{false}$ is similar. $C' = C_1$ and by the definition of EPP

$$[\![C]\!] \equiv_{\mathsf{C}} \mathtt{if}\ \mathsf{p}.e\ \{[\![C_1]\!]_{\mathsf{p}}\}\ \mathtt{else}\ \{[\![C_2]\!]_{\mathsf{p}}\}\ |\ \prod_{\mathsf{q} \in \mathbf{fp}(C')\setminus\{\mathsf{p}\}} [\![C_1]\!]_{\mathsf{q}} \sqcup [\![C_2]\!]_{\mathsf{q}}\ |\ \prod_{l} \left(\bigsqcup_{\mathsf{r} \in \lfloor C \rfloor_l} [\![C]\!]_{\mathsf{r}} \right)$$

and

$$[\![C']\!] \equiv_{\mathsf{C}} [\![C_1]\!]_{\mathsf{p}} \mid \prod_{\mathsf{q} \in \mathbf{fp}(C')\setminus\{\mathsf{p}\}} [\![C_1]\!]_{\mathsf{q}} \mid \prod_{l} \left(\bigsqcup_{\mathsf{r} \in \lfloor C_1 \rfloor_l} [\![C_1]\!]_{\mathsf{r}} \right)$$

We can apply rules $\lfloor^{\mathsf{C}}|_{\mathsf{Par}}\rceil$, $\lfloor^{\mathsf{C}}|_{\mathsf{Eq}}\rceil$, and $\lfloor^{\mathsf{C}}|_{\mathsf{Cond}}\rceil$ such that $D,[\![C]\!] \xrightarrow{\tau@\mathsf{p}} D,C''$ where

$$C'' = [\![C_1]\!]_{\mathsf{p}} \mid \prod_{\mathsf{q} \in \mathbf{fp}(C')\setminus\{\mathsf{p}\}} [\![C_1]\!]_{\mathsf{q}} \sqcup [\![C_2]\!]_{\mathsf{q}} \mid \prod_{l} \left(\bigsqcup_{\mathsf{r} \in \lfloor C \rfloor_l} [\![C]\!]_{\mathsf{r}} \right)$$

and $[\![C']\!] \prec C''$ by Lemma 12.

Case $\lfloor^{\mathsf{C}}|_{\mathsf{Ctx}}\rceil$ and **Case** $\lfloor^{\mathsf{C}}|_{\mathsf{Par}}\rceil$

proved by the definition of EPP and the induction hypothesis.

Case $\lfloor^{\mathsf{C}}|_{\mathsf{Eq}}\rceil$

We can write the derivation

$$\frac{\mathcal{R} \in \{\equiv_{\mathsf{C}}, \simeq_{\mathsf{C}}\} \quad C_1 \mathcal{R} C_1' \quad D,C_1' \xrightarrow{\beta} D',C_2' \quad C_2' \mathcal{R} C_2}{D,C_1 \xrightarrow{\beta} D',C_2} \lfloor^{\mathsf{C}}|_{\mathsf{Eq}}\rceil$$

For $\mathcal{R} = \equiv_{\mathsf{C}}$, proved by the definition of EPP, Lemma 10, and the induction hypothesis.
For $\mathcal{R} = \simeq_{\mathsf{C}}$, proved by the definition of EPP, Lemma 9, and the induction hypothesis.

□

Proof (Soundness) Proof by induction on the structure of C.

Case $C = k\colon \mathsf{p}[\mathtt{A}].e \longrightarrow \mathsf{q}[\mathtt{B}].o(x);C_c$
From the definition of EPP we have

$$[\![C]\!] \equiv_{\mathsf{C}} k\colon \mathsf{p}[\mathtt{A}].e \longrightarrow \mathtt{B}.o;[\![C_c]\!]_{\mathsf{p}} \mid k\colon \mathtt{A} \longrightarrow \mathsf{q}[\mathtt{B}].o(x);[\![C_c]\!]_{\mathsf{q}} \mid \prod_{\mathsf{r} \in \mathbf{fp}(C)} [\![C_c]\!]_{\mathsf{r}} \mid \prod_{l} \left(\bigsqcup_{\mathsf{s} \in \lfloor C \rfloor_l} [\![C]\!]_{\mathsf{s}} \right)$$

we proceed by subcases on the last applied rule in the derivation of $D,[\![C]\!] \xrightarrow{\beta} D',C''$.

Case $\lfloor^{\mathsf{C}}|_{\mathsf{Send}}\rceil$

Divided into subcases whether $\beta = k\colon \mathsf{p}[\mathtt{A}] \longrightarrow \mathtt{B}.o$ holds or not.

Case $\beta = k\colon \mathsf{p}[\mathtt{A}] \longrightarrow \mathtt{B}.o$
$D,[\![C]\!]$ reduces to D',C'' with rules $\lfloor^{\mathsf{C}}|_{\mathsf{Par}}\rceil$, $\lfloor^{\mathsf{C}}|_{\mathsf{Eq}}\rceil$, ending with rule $\lfloor^{\mathsf{C}}|_{\mathsf{Send}}\rceil$ such that

$$C'' = [\![C_c]\!]_{\mathsf{p}} \mid k\colon \mathtt{A} \longrightarrow \mathsf{q}[\mathtt{B}].o(x);[\![C_c]\!]_{\mathsf{q}} \mid \prod_{\mathsf{r} \in \mathbf{fp}(C)\setminus\{\mathsf{p},\mathsf{q}\}} [\![C_c]\!]_{\mathsf{r}} \mid \prod_{l} \left(\bigsqcup_{\mathsf{s} \in \lfloor C \rfloor_l} [\![C]\!]_{\mathsf{s}} \right)$$

D,C mimics $D,[\![C]\!]$ with rules $\lfloor^{\mathsf{C}}|_{\mathsf{Eq}}\rceil$ and $\lfloor^{\mathsf{C}}|_{\mathsf{Send}}\rceil$ for which $D,C \xrightarrow{\beta} D'',C'$, $D' = D''$ by rule $\lfloor^{\mathsf{D}}|_{\mathsf{Send}}\rceil$,

$$[\![C']\!] \equiv_{\mathsf{C}} [\![C_c]\!]_{\mathsf{p}} \mid k\colon \mathtt{A} \longrightarrow \mathsf{q}[\mathtt{B}].o(x); [\![C_c]\!]_{\mathsf{q}} \mid \prod_{\mathsf{r} \in \mathbf{fp}(C)\setminus\{\mathsf{p},\mathsf{q}\}} [\![C_c]\!]_{\mathsf{r}} \mid \prod_{l} \left(\bigsqcup_{\mathsf{s} \in \lfloor C \rfloor_l} [\![C]\!]_{\mathsf{s}} \right)$$

and $[\![C']\!] \prec C''$.

Case $\beta \neq k\colon \mathsf{p}[\mathtt{A}] \longrightarrow \mathtt{B}.o$

In this case D,C can mimic $D,[\![C]\!]$ with the application of rules $\lfloor^{\mathsf{C}}|_{\mathsf{Eq}}\rceil$, $\lfloor^{\mathsf{C}}|_{\mathsf{Par}}\rceil$, and $\lfloor^{\mathsf{C}}|_{\mathsf{Send}}\rceil$ and the thesis follows by the induction hypothesis.

Case $\lfloor^{\mathsf{C}}|_{\mathsf{Recv}}\rceil$, $\lfloor^{\mathsf{C}}|_{\mathsf{PStart}}\rceil$, or $\lfloor^{\mathsf{C}}|_{\mathsf{Cond}}\rceil$

In this case $D,[\![C]\!]$ reduces with rules $\lfloor^{\mathsf{C}}|_{\mathsf{Eq}}\rceil$, $\lfloor^{\mathsf{C}}|_{\mathsf{Par}}\rceil$, and respectively ends the derivation with either $\lfloor^{\mathsf{C}}|_{\mathsf{Recv}}\rceil$, $\lfloor^{\mathsf{C}}|_{\mathsf{PStart}}\rceil$, or $\lfloor^{\mathsf{C}}|_{\mathsf{Cond}}\rceil$, i.e., some process $\mathsf{r} \in \mathbf{fp}(C)$ (p and q included) either receives a message, starts a new session with some service processes, or reduces to some branch. D,C can mimic $D,[\![C]\!]$ applying rules $\lfloor^{\mathsf{C}}|_{\mathsf{Eq}}\rceil$, $\lfloor^{\mathsf{C}}|_{\mathsf{Par}}\rceil$ and terminates the derivation with either rules $\lfloor^{\mathsf{C}}|_{\mathsf{Recv}}\rceil$, $\lfloor^{\mathsf{C}}|_{\mathsf{PStart}}\rceil$ (or $\lfloor^{\mathsf{C}}|_{\mathsf{Start}}\rceil$, depending on the form of C) or $\lfloor^{\mathsf{C}}|_{\mathsf{Cond}}\rceil$. The thesis follows by the induction hypothesis.

Case $C = k\colon \mathsf{p}[\mathtt{A}].e \longrightarrow \mathtt{B}.o; C_c$

Similar to case $C = k\colon \mathsf{p}[\mathtt{A}].e \longrightarrow \mathsf{q}[\mathtt{B}].o(x); C_c$.

Case $C = k\colon \mathtt{A} \longrightarrow \mathsf{q}[\mathtt{B}].\{o_i(x_i); C_i\}_{i \in I}$

From the definition of EPP we have

$$[\![C]\!] \equiv_{\mathsf{C}} k\colon \mathtt{A} \longrightarrow \mathsf{q}[\mathtt{B}].\{o_i(x_i); [\![C_i]\!]_{\mathsf{q}}\}_{i \in I} \mid \prod_{i \in I} \left(\bigsqcup_{\mathsf{p} \in \mathbf{fp}(C_i)} [\![C_i]\!]_{\mathsf{p}} \right) \mid \prod_{k} \left(\bigsqcup_{\mathsf{r} \in \lfloor C \rfloor_l} [\![C]\!]_{\mathsf{r}} \right)$$

we proceed by subcases on the last applied rule in the derivation of $D,[\![C]\!] \xrightarrow{\beta} D',C''$.

Case $\lfloor^{\mathsf{C}}|_{\mathsf{Recv}}\rceil$

Divided into subcases whether $\beta = k\colon \mathtt{A}\rangle \mathsf{q}[\mathtt{B}].o_j,\ j \in I$ or not.

Case $\beta = k\colon \mathtt{A}\rangle \mathsf{q}[\mathtt{B}].o_j,\ j \in I$

$D,[\![C]\!]$ reduces to D',C'' with rules $\lfloor^{\mathsf{C}}|_{\mathsf{Par}}\rceil$, $\lfloor^{\mathsf{C}}|_{\mathsf{Eq}}\rceil$, and terminates with rule $\lfloor^{\mathsf{C}}|_{\mathsf{Recv}}\rceil$ such that

$$C'' = [\![C_j]\!]_{\mathsf{q}} \mid \prod_{i \in I} \left(\bigsqcup_{\mathsf{p} \in \mathbf{fp}(C_i)\setminus\{\mathsf{q}\}} [\![C_i]\!]_{\mathsf{p}} \right) \mid \prod_{k} \left(\bigsqcup_{\mathsf{r} \in \lfloor C \rfloor_l} [\![C]\!]_{\mathsf{r}} \right)$$

D,C mimics $D,[\![C]\!]$ with rule $\lfloor^{\mathsf{C}}|_{\mathsf{Recv}}\rceil$ for which $D,C \xrightarrow{\beta} D'',C'$ where $D'' = D'$ by rule $\lfloor^{\mathsf{D}}|_{\mathsf{Recv}}\rceil$ and

$$[\![C']\!] = [\![C_j]\!]_{\mathsf{q}} \mid \prod_{\mathsf{p} \in \mathbf{fp}(C_j)\setminus\{\mathsf{q}\}} [\![C_j]\!]_{\mathsf{p}} \mid \prod_{k} \left(\bigsqcup_{\mathsf{r} \in \lfloor C_j \rfloor_l} [\![C_j]\!]_{\mathsf{r}} \right)$$

and $[\![C']\!] \prec C''$ by Lemma 12.

Case $\beta \neq k\colon \mathtt{A}\rangle \mathsf{q}[\mathtt{B}].o_j$

For any β of this case D,C can mimic $D,[\![C]\!]$ with the application of rules $\lfloor^{\mathsf{C}}|_{\mathsf{Eq}}\rceil$ and $\lfloor^{\mathsf{C}}|_{\mathsf{Par}}\rceil$, terminating with rule $\lfloor^{\mathsf{C}}|_{\mathsf{Recv}}\rceil$ and the thesis follows by the induction hypothesis.

Case $\lfloor^{\mathsf{C}}|_{\mathsf{Send}}\rceil$, $\lfloor^{\mathsf{C}}|_{\mathsf{PStart}}\rceil$, or $\lfloor^{\mathsf{C}}|_{\mathsf{Cond}}\rceil$

is similar to subcase **Case** $\lfloor^{\mathsf{C}}|_{\mathsf{Recv}}\rceil$, $\lfloor^{\mathsf{C}}|_{\mathsf{PStart}}\rceil$, or $\lfloor^{\mathsf{C}}|_{\mathsf{Cond}}\rceil$ of **Case** $C = k\colon \mathsf{p}[\mathtt{A}].e \longrightarrow \mathsf{q}[\mathtt{B}].o(x);C_c$.

Case $C = \mathtt{start}\ k : \mathsf{p}[\mathtt{A}] \Leftrightarrow \widetilde{l.\mathsf{q}[\mathtt{B}]};C_c$

$$[\![C]\!] \equiv_{\mathsf{C}} \mathtt{req}\ k : \mathsf{p}[\mathtt{A}] \Leftrightarrow \widetilde{l.\mathtt{B}};C_c \mid \prod_{\mathsf{r} \in \mathbf{fp}(C_c)\setminus\{\mathsf{p}\}} [\![C_c]\!]_{\mathsf{r}} \mid \prod_{l} \left(\bigsqcup_{\mathsf{s} \in \lfloor C \rfloor_l} [\![C]\!]_{\mathsf{s}} \right)$$

we proceed by subcases on the last applied rule in the derivation of $D,[\![C]\!] \xrightarrow{\beta} D,C''$.

Case $\lfloor^{\mathsf{C}}|_{\mathsf{PStart}}\rceil$

$D,[\![C]\!]$ can reduce to D',C'' with a process r (including p) that starts a new session with some service processes. D,C can reduce to D'',C' mimicking $D,[\![C]\!]$ by applying rules $\lfloor^{\mathsf{C}}|_{\mathsf{Eq}}\rceil$, $\lfloor^{\mathsf{C}}|_{\mathsf{Par}}\rceil$, terminating with either rule $\lfloor^{\mathsf{C}}|_{\mathsf{PStart}}\rceil$ or $\lfloor^{\mathsf{C}}|_{\mathsf{Start}}\rceil$.

Case $\lfloor^{\mathsf{C}}|_{\mathsf{Send}}\rceil$, $\lfloor^{\mathsf{C}}|_{\mathsf{Recv}}\rceil$, and $\lfloor^{\mathsf{C}}|_{\mathsf{Cond}}\rceil$

are similar to the corresponding proof for the previous cases.

Case $C = \mathtt{if}\ \mathsf{p}.e\ \{C_1\}\ \mathtt{else}\ \{C_2\}$

From the definition of EPP we have

$$[\![C]\!] \equiv_{\mathsf{C}} \mathtt{if}\ \mathsf{p}.e\ \{[\![C_1]\!]_{\mathsf{p}}\}\ \mathtt{else}\ \{[\![C_2]\!]_{\mathsf{p}}\} \mid \prod_{\mathsf{q} \in \mathbf{fp}(C_1) \cup \mathbf{fp}(C_2)\setminus\{\mathsf{p}\}} [\![C_1]\!]_{\mathsf{q}} \sqcup [\![C_2]\!]_{\mathsf{q}} \mid \mid \prod_{l} \left(\bigsqcup_{\mathsf{r} \in \lfloor C \rfloor_l} [\![C]\!]_{\mathsf{r}} \right)$$

we proceed by subcases on the derivation of $D,[\![C]\!] \xrightarrow{\beta} D',C''$.

Case $\lfloor^{\mathsf{C}}|_{\mathsf{Cond}}\rceil$

$D,[\![C]\!]$ can reduce to D',C'' with:

Case $\beta = \tau@\mathsf{p}$

that reduces to a branch. D,C can mimic $D,[\![C]\!]$ applying rules $\lfloor^{\mathsf{C}}|_{\mathsf{Eq}}\rceil$, $\lfloor^{\mathsf{C}}|_{\mathsf{Par}}\rceil$, and terminating the derivation with rule $\lfloor^{\mathsf{C}}|_{\mathsf{Cond}}\rceil$. The case is proved by Lemma 12.

Case $\beta = \tau@\mathsf{r},\ \mathsf{r} \neq \mathsf{p}$

where process r reduced to a branch. The case follows the proof of the previous case and the thesis follows by the induction hypothesis.

Case $\lfloor^{\mathsf{C}}|_{\mathsf{Recv}}\rceil$, $\lfloor^{\mathsf{C}}|_{\mathsf{Send}}\rceil$, $\lfloor^{\mathsf{C}}|_{\mathsf{PStart}}\rceil$

are similar to the corresponding proof for the previous cases.

Case $C = \mathtt{req}\ k : \mathsf{p}[\mathtt{A}] \Leftrightarrow \widetilde{l.\mathtt{B}};C_c$

Case not allowed by the hypothesis that $D,[\![C]\!] \xrightarrow{\beta} D,C''$.

Case $C = \mathtt{acc}\ k : \widetilde{l.\mathsf{q}[\mathtt{B}]};C_c$

Case not allowed by the hypothesis that $D,[\![C]\!] \xrightarrow{\beta} D,C''$.

Case $C = \mathtt{def}\ X = C''\ \mathtt{in}\ C'$

proved by Lemma 10 and the induction hypothesis.

Case $C = X$

Case not allowed by the hypothesis that C is well-sorted.

Case $C = C_1 \mid C_2$

$[\![C]\!] \equiv_{\mathsf{C}} [\![C_1]\!] \mid [\![C_2]\!]$ by Lemma 11.

we proceed by subcases for n equal to the length of the derivation of $D, [\![C]\!] \xrightarrow{\beta} D', C''$

Case $n = 1$

In this case the only applicable rule is $\lfloor^{\mathsf{C}}|_{\mathsf{PStart}}\rceil$ where, Since both $[\![C_1]\!]$ and $[\![C_2]\!]$ reduce, we can infer, let

$$\widetilde{l.\mathsf{q}[\mathtt{B}]} = l_1.\mathsf{q}_1[\mathtt{B}_1], \ldots, l_i.\mathsf{q}_i[\mathtt{B}_i], l_{i+1}.\mathsf{q}_{i+1}[\mathtt{B}_{i+1}] \ldots, l_n.\mathsf{q}_n[\mathtt{B}_n]$$

that

$$C_1 \equiv_{\mathsf{C}} \mathsf{req}\ k : \mathsf{p}[\mathtt{A}] \Leftrightarrow \widetilde{l.\mathtt{B}}; C_1^r \mid \prod_{j=1}^{i} \mathsf{acc}\ k : l_j.\mathsf{q}_j[\mathtt{B}_j]; C_1^j \mid C_c^1$$

$$C_2 \equiv_{\mathsf{C}} \prod_{j=i+1}^{n} \mathsf{acc}\ k : l_j.\mathsf{q}_j[\mathtt{B}_j]; C_2^j \mid C_c^2$$

and by the definition of EPP that

$$[\![C_1]\!] \equiv_{\mathsf{C}} \mathsf{req}\ k : \mathsf{p}[\mathtt{A}] \Leftrightarrow \widetilde{l.\mathtt{B}}; [\![C_1^r]\!]_{\mathsf{p}} \mid \prod_{j=1}^{i} \mathsf{acc}\ k : l_j.\mathsf{q}_j[\mathtt{B}_j]; \left[\!\!\left[C_1^j\right]\!\!\right]_{\mathsf{q}_j} \mid \left[\!\!\left[C_c^1\right]\!\!\right]$$

$$[\![C_2]\!] \equiv_{\mathsf{C}} \prod_{j=i+1}^{n} \mathsf{acc}\ k : l_j.\mathsf{q}_j[\mathtt{B}_j]; \left[\!\!\left[C_2^j\right]\!\!\right]_{\mathsf{q}_j} \mid \left[\!\!\left[C_c^2\right]\!\!\right]$$

Observe that we can proceed without loss of generality as the symmetric case (with $\mathsf{p} \in \mathbf{fp}(C_2)$) follows the same structure.

$$\frac{\begin{array}{c} i \in \{1,\ldots,n\} \qquad D\#k', \tilde{\mathsf{r}} \qquad \{\widetilde{l.\mathtt{B}}\} = \biguplus_i \{\widetilde{l_i.\mathtt{B}_i}\}_i \qquad \{\tilde{\mathsf{r}}\} = \bigcup_i \{\tilde{\mathsf{r}}_i\} \\ \delta = \mathsf{start}\ k' : \mathsf{p}[\mathtt{A}] \Leftrightarrow \widetilde{l_1.\mathsf{r}_1[\mathtt{B}_1]}, \ldots, \widetilde{l_n.\mathsf{r}_n[\mathtt{B}_n]} \qquad D, \delta \blacktriangleright D'' \end{array}}{D, [\![C_1]\!] \mid [\![C_2]\!] \quad \xrightarrow{\tau} \quad D'', C''} \lfloor^{\mathsf{C}}|_{\mathsf{PStart}}\rceil$$

where

$$C'' \equiv_{\mathsf{C}} \begin{cases} [\![C_1^r]\!]_{\mathsf{p}}[k'/k] \mid \begin{pmatrix} \prod_{j=1}^{i} \left[\!\!\left[C_1^j\right]\!\!\right]_{\mathsf{q}_j} \\ \mid \prod_{j=i+1}^{n} \left[\!\!\left[C_2^j\right]\!\!\right]_{\mathsf{q}_j} \end{pmatrix} [k'/k][\tilde{\mathsf{r}}/\tilde{\mathsf{q}}] \\ \mid \begin{pmatrix} \prod_{j=1}^{i} \mathsf{acc}\ k : l_j.\mathsf{q}_j[\mathtt{B}_j]; \left[\!\!\left[C_1^j\right]\!\!\right]_{\mathsf{q}_j} \\ \mid \prod_{j=i+1}^{n} \mathsf{acc}\ k : l_j.\mathsf{q}_j[\mathtt{B}_j]; \left[\!\!\left[C_2^j\right]\!\!\right]_{\mathsf{q}_j} \end{pmatrix} \mid [\![C_c^1]\!] \mid [\![C_c^2]\!] \end{cases}$$

Then D, C can mimic $D, [\![C]\!]$ applying rule $\lfloor^{\mathsf{C}}|_{\mathsf{PStart}}\rceil$ with reduction

$$\frac{\begin{array}{c} i \in \{1,\ldots,n\} \quad D\#k'',\tilde{\mathsf{r}}' \quad \{\widetilde{l.\mathtt{B}}\} = \biguplus_i \{\widetilde{l_i.\mathtt{B}_i}\}_i \quad \{\tilde{\mathsf{r}}'\} = \bigcup_i \{\tilde{\mathsf{r}}'_i\} \\ \delta = \mathtt{start}\ k'' : \mathsf{p}[\mathtt{A}] \twoheadleftarrow\!\!\twoheadrightarrow \widetilde{l_1.\mathsf{r}'_1[\mathtt{B}_1]},\ldots,\widetilde{l_n.\mathsf{r}'_n[\mathtt{B}_n]} \quad D,\delta \blacktriangleright D'' \end{array}}{D, C_1 \mid C_2 \quad \xrightarrow{\tau} \quad D'', C'} \lfloor^{\mathsf{C}}|_{\mathsf{PStart}}\rceil$$

where

$$C' \equiv_{\mathsf{C}} C_1^r[k''/k] \mid \begin{pmatrix} \prod_{j=1}^{i} C_1^j \mid \\ \prod_{j=i+1}^{n} C_2^j \end{pmatrix} [k''/k][\tilde{\mathsf{r}}'/\tilde{\mathsf{q}}] \mid \begin{pmatrix} \prod_{j=1}^{i} \mathsf{acc}\ k : l_j.\mathsf{q}_j[\mathtt{B}_j]; C_1^j \\ \mid \prod_{j=i+1}^{n} \mathsf{acc}\ k : l_j.\mathsf{q}_j[\mathtt{B}_j]; C_2^j \end{pmatrix} \mid [\![C_c^1]\!] \mid [\![C_c^2]\!]$$

Following the structure of the second part of the proof of **Case** $\lfloor^{\mathsf{C}}|_{\mathsf{Start}}\rceil$ for the proof of *Completeness* of Theorem 2 of the article, by α-renaming we have $D'' = D'$ and $[\![C']\!] \prec C''$.

Case $n > 1$

For $n > 1$ we have a derivation similar to

$$\frac{\begin{array}{c} R \\ \vdots \quad n-1 \text{ times, each either } \lfloor^{\mathsf{C}}|_{\mathsf{Par}}\rceil \text{ or } \lfloor^{\mathsf{C}}|_{\mathsf{Eq}}\rceil \end{array}}{D, [\![C_1]\!] \mid [\![C_2]\!] \quad \xrightarrow{\beta} \quad D', C_1'' \mid [\![C_2]\!]} \lfloor^{\mathsf{C}}|_{\mathsf{Par}}\rceil$$

where R is the last applied rule, $R \in \{\lfloor^{\mathsf{C}}|_{\mathsf{Send}}\rceil, \lfloor^{\mathsf{C}}|_{\mathsf{Recv}}\rceil, \lfloor^{\mathsf{C}}|_{\mathsf{PStart}}\rceil, \lfloor^{\mathsf{C}}|_{\mathsf{Cond}}\rceil\}$. The thesis follows from the induction hypothesis.

The proof for the mirror case $D, [\![C_1]\!] \mid [\![C_2]\!] \xrightarrow{\beta} D', [\![C_1]\!] \mid C_2''$ follows the same structure.

Case $C = \mathbf{0}$
trivial.

□

3.8 Proof of Compilation from Frontend Choreographies to DCC Networks

We first define some auxiliary results used in the proof of Theorem 3 of the article.

We provide some results on DCC variable substitution. Note that the only bound names in DCC are the variables in (acc) terms (e.g., x in $!(x);B$). However, the following lemmas prove that renaming free variables with fresh names in processes (and, by extension, in services) preservers bisimilarity.

In the following, we abuse the notation for α-renaming to denote variable renaming in running processes. We define the variable renaming operator for DCC processes $P[x'/x]$.

Definition 12 (DCC Variable Renaming Operator)**.** Let $B \cdot t$ be a DCC process, then $(B \cdot t)[x'/x] = B[x'/x] \cdot t \triangleleft (\, x', x(t)\,) \triangleleft (\, x, \varnothing \,)$ where $B[x'/x]$ substitutes every occurrence of x with x'.

Lemma 13 (DCC Process Variable Renaming)**.** Let $\langle \mathfrak{B}, P \mid P_c, M\rangle_l$ be a DCC service where $P = B \cdot t$. Let $P' = P[x'/x]$ where x' is fresh in B. Then $\langle \mathfrak{B}, P \mid P_c, M\rangle_l \to \langle \mathfrak{B}, P'' \mid P_c, M\rangle_l \iff \langle \mathfrak{B}, P' \mid P_c, M\rangle_l \to \langle \mathfrak{B}, P''[x'/x] \mid P_c, M\rangle_l$.

Proof The proof is by induction on the form of P. We report the most interesting cases. Below we consider $t' = t \triangleleft (\, x', x(t) \,) \triangleleft (\, x, \varnothing \,)$.

Case $P = o(y)\ \texttt{from}\ e; B' \cdot t$
The only applicable rule is $\lfloor^{\mathsf{DCC}}|_{\mathsf{Recv}}\rceil$, hence we consider the interesting case in which M contains a message for the queue defined by e. In the other case the Lemma trivially holds as services cannot reduce on P and P'. The case unfolds on the combinations of whether *i)* $y \neq x$ and *ii)* expression e contains x. Below we consider the comprehensive case for $y = x$ and e that contains x. The proof of the other cases is either trivial or a slight modification of the reported one.

Since we assume we can apply rule $\lfloor^{\mathsf{DCC}}|_{\mathsf{Recv}}\rceil$ we take $t_c = \mathbf{eval}(e, t)$ and $M(t_c) = (o, t') :: \tilde{m}$. From Definition 12 we have that $t_c = \mathbf{eval}(e[x'/x], t')$.

Meaningful reductions on P and P' are of the form $P \to B' \cdot t \triangleleft (\, x, t_m \,)$ and $P' \to B'[x'/x] \cdot t' \triangleleft (\, x', t_m \,)$ and the thesis follow by induction hypothesis.

Case $P = \sum_{i \in I} [o_i(x_i)\ \texttt{from}\ e]\ \{B_i\} \cdot t$
The only applicable rule on both P and P' is $\lfloor^{\mathsf{DCC}}|_{\mathsf{Recv}}\rceil$. The most comprehensive case is for M that contains a message for operation o_j, $j \in I$ where $x_j = x$ and expression e contains x. The remainder of the proof follows that of the previous case.

Case $P = \texttt{if}\ e\ \{B_1\}\ \texttt{else}\ \{B_2\} \cdot t$
Trivial by Definition 12 for which $\mathbf{eval}(e, t) = \mathbf{eval}(e[x'/x], t')$.

Case $P = y = e; B \cdot t$
The only applicable rule on both P and P' is $\lfloor^{\mathsf{DCC}}|_{\mathsf{Assign}}\rceil$. The most comprehensive case is for $y = x$ and expression e that contains x. The case is proved considering that, by Definition 12, it holds that $\mathbf{eval}(e, t) = \mathbf{eval}(e[x'/x], t')$.

Case $P = \texttt{def}\ X = B_1\ \texttt{in}\ B \cdot t$
The thesis follows from the application of rule $\lfloor^{\mathsf{DCC}}|_{\mathsf{Ctx}}\rceil$ and the induction hypothesis.

Case $P = \nu\rangle x; B' \cdot t$
Let $t_c \notin M$. We have the reduction on rule $\lfloor^{\mathsf{DCC}}|_{\mathsf{Newque}}\rceil$

$$S \to \left\langle \mathfrak{B}, B' \cdot t \triangleleft (\, x, t_c \,) \mid P_c, M[t_c \mapsto \varepsilon] \right\rangle_l$$

Let service S' be equal to S with P replaced with P'. S' can mimic the behaviour of S by taking the fresh value $t'_c = t_c$, obtaining the reduction

$$S' \to \left\langle \mathfrak{B}, B'[x'/x] \cdot t' \triangleleft (\, x', t'_c \,) \mid P_c, M[t'_c \mapsto \varepsilon] \right\rangle_l$$

The same holds if we let S' reduce and prove that S can mimic it.

Case $P = o@e_1(e_2)\ \texttt{to}\ e_3; B' \cdot t$
We consider the comprehensive case in which expressions e_1, e_2 and e_3 contain x. From Definition 12 we know that $\mathbf{eval}(e_1, t) = \mathbf{eval}(e_1[x'/x], t')$. Similarly the couples e_2 and $e_2[x'/x]$ and e_3 and $e_3[x'/x]$ enjoy the same property when evaluated respectively on t and t'.

We analyse the case in which P moves and $P[x'/x]$ mimics it. The other case, for $P[x'/x]$ that reduces and P that mimics it, follows the same structure.

$$\frac{\begin{array}{c} B = o@e_1(e_2)\ \texttt{to}\ e_3; B' \qquad \mathbf{eval}(e_1, t) = l \\ \mathbf{eval}(e_3, t) = t_c \qquad \mathbf{eval}(e_2, t) = t_m \qquad t_c \in \mathbf{dom}(M) \end{array}}{\langle \mathfrak{B},\ B \cdot t \mid P, M \rangle_l \quad \to \quad \langle \mathfrak{B},\ B' \cdot t \mid P, M[t_c \mapsto M(t_c) :: (o, t_m)] \rangle_l}\ \lfloor^{\mathsf{DCC}}|_{\mathsf{InSend}}\rceil$$

and

$$\frac{\begin{array}{c} B[x'/x] = o@e_1[x'/x](e_2[x'/x])\ \texttt{to}\ e_3[x'/x]; B'[x'/x] \qquad \mathbf{eval}(e_1[x'/x], t') = l \\ \mathbf{eval}(e_3[x'/x], t') = t_c \qquad \mathbf{eval}(e_2[x'/x], t') = t_m \qquad t_c \in \mathbf{dom}(M) \end{array}}{\langle \mathfrak{B},\ B[x'/x] \cdot t' \mid P, M \rangle_l \quad \to \quad \langle \mathfrak{B},\ B'[x'/x] \cdot t' \mid P, M[t_c \mapsto M(t_c) :: (o, t_m)] \rangle_l}\ \lfloor^{\mathsf{DCC}}|_{\mathsf{InSend}}\rceil$$

Case $?@e_1(e_2);B''\cdot t$
We consider the comprehensive case where expressions e_1 and e_2 contain x. From Definition 12 we know that $\mathbf{eval}(e_1,t) = \mathbf{eval}(e_1[x'/x],t')$. Similarly e_2 and $e_2[x'/x]$ enjoy the same property when evaluated respectively on t and t'.

Below we describe the case in which P moves and $P[x'/x]$ mimics it. The other case, for $P[x'/x]$ that reduces and P that mimics it, follows the same structure. We assume the start behaviour $\mathfrak{B} = !(y);B'$.

$$\frac{B = ?@e_1(e_2);B'' \quad Q = B'\cdot\varnothing \lhd (y, \mathbf{eval}(e_2,t))}{\langle !(y);B',\ B\cdot t \mid P_c,\ M\rangle_l \quad \to \quad \langle !(y);B',\ Q \mid B''\cdot t \mid P_c,\ M\rangle_l} \lfloor^{\mathsf{DCC}}|_{\mathsf{InStart}}\rceil$$

and

$$\frac{B[x'/x] = ?@e_1[x'/x](e_2[x'/x]);B''[x'/x] \quad Q = B'\cdot\varnothing \lhd (y, \mathbf{eval}(e_2[x'/x],t'))}{\langle !(y);B',\ B[x'/x]\cdot t' \mid P_c,\ M\rangle_l \quad \to \quad \langle !(y);B',\ Q \mid B''[x'/x]\cdot t' \mid P_c,\ M\rangle_l} \lfloor^{\mathsf{DCC}}|_{\mathsf{InStart}}\rceil$$

□

Lemma 14 (DCC Network Variable Renaming)**.** Let S and S' be two DCC networks such that $S = \langle \mathfrak{B}, P \mid Q, M\rangle_l \mid S_*$ and $S' = \langle \mathfrak{B}, P[x'/x] \mid Q, M\rangle_l \mid S_*$ then

$$S \to \langle \mathfrak{B}, P' \mid Q', M'\rangle_l \mid S'_* \iff S' \to \langle \mathfrak{B}, P'[x'/x] \mid Q', M'\rangle_l \mid S'_*$$

.

Proof Sketch The proof is by induction on the derivation of S. The main observation is that the most part of cases are already considered in Lemma 13. The cases not considered in Lemma 13 regard derivations on rules:

- $\lfloor^{\mathsf{DCC}}|_{\mathsf{Send}}\rceil$ whose proof follows the same steps of case $P = o@e_1(e_2)$ $\texttt{to}$ $e_3;B'\cdot t$ in Lemma 13;
- $\lfloor^{\mathsf{DCC}}|_{\mathsf{Start}}\rceil$ proved following the same steps of case $P = ?@e_1(e_2);B''\cdot t$ in Lemma 13;
- $\lfloor^{\mathsf{DCC}}|_{\mathsf{Eq}}\rceil$ and $\lfloor^{\mathsf{DCC}}|_{\mathsf{Par}}\rceil$ where the thesis follows from the application of the induction hypothesis.

□

We report below the statement of Theorem 6 of the article, enriched with annotation on the transitions of D,C.

Theorem 6 *(Applied Choreographies)*
Let D,C be a Frontend choreography where C is projectable and $\Gamma \vdash D,C$ for some Γ. Then:

1. (Completeness) $D,C \xrightarrow{\beta} D',C'$ implies

   (a) $\boxed{\langle\!\langle D\rangle\!\rangle^{\Gamma}, [\![C]\!]}^{\Gamma} \to^{+} \boxed{\langle\!\langle D'\rangle\!\rangle^{\Gamma'}, C''}^{\Gamma'}$

   (b) $[\![C']\!] \prec C''$

   (c) for some Γ', $\Gamma' \vdash D',C'$

2. (Soundness) $\boxed{\langle\!\langle D\rangle\!\rangle^{\Gamma}, [\![C]\!]}^{\Gamma} \to^{*} S$ implies

   (a) $D,C \to^{*} D',C'$

(b) $S \to^* \boxed{\langle\!\langle D' \rangle\!\rangle^{\Gamma'}, C''}^{\Gamma'}$

(c) $[\![C']\!] \prec C''$

(d) for some Γ', $\Gamma' \vdash D', C'$

Proof (Completeness) We proceed by induction on the derivation of $D, C \xrightarrow{\beta} D', C'$. The general strategy is to:

- apply Theorem 1 of the article from which, let $\mathbb{D} = \langle\!\langle D \rangle\!\rangle^{\Gamma}$, we have that $\mathbb{D}, C \xrightarrow{\beta} \mathbb{D}', C'$, $\mathbb{D}' = \langle\!\langle D \rangle\!\rangle^{\Gamma'}$;
- since C is *projectable*, we can always apply Theorem 2 of the article, from which, $D, [\![C]\!] \xrightarrow{\beta} D', C''$ and $[\![C']\!] \prec C''$;
- we compile the Backend Endpoint choreography $\mathbb{D}, [\![C]\!]$ into the DCC network $\boxed{\mathbb{D}, [\![C]\!]}^{\Gamma}$ and prove that we can reduce it in such a way that its reductum is $\equiv_{\mathsf{D}}$-equivalent to the compilation of the reductum $\boxed{\langle\!\langle D' \rangle\!\rangle^{\Gamma'}, C''}^{\Gamma'}$.

  Case $\lfloor^{\mathsf{C}}|_{\mathsf{Send}}\rceil$
  We know that

  – $[\![C]\!] \equiv_{\mathsf{C}} C_{\mathsf{p}} \mid C_c$ with $C_{\mathsf{p}} = k\colon \mathsf{p}[\mathtt{A}].e \longrightarrow \mathtt{B}.o; C'_p$;

  – $D, [\![C]\!] \xrightarrow{\beta} D', C''$ with $\lfloor^{\mathsf{C}}|_{\mathsf{Send}}\rceil$ being the last applied rule, where $\beta = k\colon \mathsf{p}[\mathtt{A}].e \longrightarrow \mathtt{B}.o$ and $C'' = C'_{\mathsf{p}} \mid C_c$;

  – let $\tilde{m} = D(k[\mathtt{A}\rangle\mathtt{B}])$ and $v = \mathbf{eval}(e, D(\mathsf{p}))$ we have, by rule $\lfloor^{\mathsf{D}}|_{\mathsf{Send}}\rceil$,

$$D' = D\big[\, k[\mathtt{A}\rangle\mathtt{B}] \mapsto \tilde{m} :: (o, v) \,\big]$$

  which, by Theorem 1 of the article, corresponds to $\mathbb{D}' = \mathbb{D}\big[\, l^* : t_c \mapsto \mathbb{D}(l^* : t_c) :: (o, t_m) \,\big]$ by $\lfloor^{\mathbb{D}}|_{\mathsf{Send}}\rceil$ where l^* is the location of the receiving process playing role $\mathtt{B}$ and t_c is the correlation key used by the process playing $\mathtt{A}$ to send to the process playing role $\mathtt{B}$. The tree t_m corresponds to value v exchanged in rule $\lfloor^{\mathsf{D}}|_{\mathsf{Send}}\rceil$.

  We have two cases, whether the receiving process q is in the same location of the sender p or not. Formally, let $\mathsf{p} \in \mathbb{D}(l)$ we consider the exhaustive cases:

  Case $\mathsf{q} \in \mathbb{D}(l)$

  From Definition 8 of the article we have that $\boxed{\mathbb{D}, [\![C]\!]}^{\Gamma} \equiv_{\mathsf{D}} S \mid S_c$ where, let $t_{\mathsf{p}} = \mathbb{D}(\mathsf{p})$ and $M = \mathbb{D}|_l$

  * $S = \left\langle \boxed{C_c|_l}^{\Gamma}, P \mid Q, M \right\rangle_l$
  * $P = o@\underline{k.B.l}(e)$ $\mathtt{to}$ $\underline{k.A.B}; \boxed{C'_{\mathsf{p}}}^{\Gamma} \cdot t_{\mathsf{p}}$
  * $Q = \prod_{\mathsf{q} \,\in\, \mathbb{D}(l) \setminus \{\mathsf{p}\}} \boxed{C_c|_{\mathsf{q}}}^{\Gamma} \cdot \mathbb{D}(\mathsf{q})$
  * $S_c = \prod_{l' \,\in\, \Gamma \setminus \{l\}} \left\langle \boxed{C_c|_{l'}}^{\Gamma}, \prod_{\mathsf{r} \,\in\, \mathbb{D}(l')} \boxed{C_c|_{\mathsf{r}}}^{\Gamma} \cdot \mathbb{D}(\mathsf{s}), \mathbb{D}|_{l'} \right\rangle_{l'}$

In this case $\boxed{\mathbb{D}, [\![C]\!]}^{\Gamma}$ can mimic D, C applying rules $\lfloor^{\mathsf{DCC}}|_{\mathsf{Eq}}\rceil$, $\lfloor^{\mathsf{DCC}}|_{\mathsf{SPar}}\rceil$, and $\lfloor^{\mathsf{DCC}}|_{\mathsf{InSend}}\rceil$ where $S \mid S_c \rightarrow S' \mid S_c$ with $\lfloor^{\mathsf{DCC}}|_{\mathsf{SPar}}\rceil$ and $S \rightarrow S'$ with

$$\frac{\begin{array}{c} P = o@\underline{k.B.l}(e) \texttt{ to } \underline{k.A.B}; \boxed{C'_{\mathsf{p}}}^{\Gamma} \cdot t_{\mathsf{p}} \qquad \mathbf{eval}(\underline{k.B.l}, t_{\mathsf{p}}) = l \\ \mathbf{eval}(\underline{k.A.B}, t_{\mathsf{p}}) = t_c \qquad \mathbf{eval}(e, t_{\mathsf{p}}) = t_m \qquad t_c \in \mathbf{dom}(M) \end{array}}{\langle \mathfrak{B}, P \mid Q, M \rangle_l \quad \rightarrow \quad \langle \mathfrak{B}, P' \mid Q, M[t_c \mapsto M(t_c) :: (o, t_m)] \rangle_l} \; \lfloor^{\mathsf{DCC}}|_{\mathsf{InSend}}\rceil$$

where $P' = \boxed{C'_{\mathsf{p}}}^{\Gamma} \cdot t_{\mathsf{p}}$. Since by Definition 8 of the article, l, t_c, and t_m result from the evaluation of the state of process p, $\mathbb{D}(\mathsf{p})$, we have that $M[t_c \mapsto M(t_c) :: (o, t_m)] = \mathbb{D}'|_l$. This corresponds to the compilation of the reduction D', C', i.e,

$$\boxed{\langle\!\langle D' \rangle\!\rangle^{\Gamma'}, C''}^{\Gamma'} \equiv_{\mathsf{D}} \begin{array}{l} \left. \left\langle \boxed{C_c|_l}^{\Gamma'}, \overbrace{\boxed{C'_{\mathsf{p}}}^{\Gamma'} \cdot \mathbb{D}'(\mathsf{p})}^{P'} \mid \overbrace{\prod_{\mathsf{q} \in \mathbb{D}'(l) \setminus \{\mathsf{p}\}} \boxed{C_c|_{\mathsf{q}}}^{\Gamma'} \cdot \mathbb{D}'(\mathsf{q})}^{Q}, \mathbb{D}'|_l \right\rangle_l \right\} S' \\ \mid \\ \left. \prod_{l' \in \Gamma' \setminus \{l\}} \left\langle \boxed{C_c|_{l'}}^{\Gamma'}, \prod_{\mathsf{r} \in \mathbb{D}'(l')} \boxed{C_c|_{\mathsf{r}}}^{\Gamma'} \cdot \mathbb{D}'(\mathsf{r}), \mathbb{D}'|_{l'} \right\rangle_{l'} \right\} S_c \end{array}$$

Where the changes in D' and Γ' affect only the compilation of the queue in $\mathbb{D}'|_l$ identified by t_c, while for all other terms $\boxed{\cdot}^{\Gamma} = \boxed{\cdot}^{\Gamma'}$ and $\mathbb{D}'|_{l'} = \mathbb{D}|_{l'}$.

Case $\mathsf{q} \notin \mathbb{D}(l)$

Similar to **Case** $\mathsf{q} \in \mathbb{D}(l)$ except the last applied rule in the reduction of $\boxed{\mathbb{D}, [\![C]\!]}^{\Gamma}$ is $\lfloor^{\mathsf{DCC}}|_{\mathsf{Send}}\rceil$.

Case $\lfloor^{\mathsf{C}}|_{\mathsf{Recv}}\rceil$
We know that

- $[\![C]\!] \equiv_{\mathsf{C}} C_{\mathsf{q}} \mid C_c$ with $C_{\mathsf{q}} = k\colon \mathtt{A} \longrightarrow \mathsf{q}[\mathtt{B}].\{o_i(x_i); C_i\}_{i \in I}$
- $D, [\![C]\!] \xrightarrow{\beta} D', C''$ with rule $\lfloor^{\mathsf{C}}|_{\mathsf{Recv}}\rceil$ where $\beta = k\colon \mathtt{A}\rangle\mathsf{q}[\mathtt{B}].o_j(x_j)$, $C' \equiv_{\mathsf{C}} C_j \mid C_c$. Let $\mathbb{D} = \langle\!\langle D \rangle\!\rangle^{\Gamma}$ and $D(k[\mathtt{A}\rangle\mathtt{B}]) = (o_j, v) :: \tilde{m}$, we have

  $$D' = D[\, \mathsf{q} \mapsto D(\mathsf{q})[x_j \mapsto v] \,][\, k[\mathtt{A}\rangle\mathtt{B}] \mapsto \tilde{m} \,]$$

  By Theorem 1 of the article, let $\mathbb{D}(t_c : l^*) = (o_j, t_m) :: \tilde{m}^*$ we have

  $$\mathbb{D}' = \mathbb{D}[\, \mathsf{q} \mapsto \mathbb{D}(\mathsf{q})[x_j \rightarrow t_m]][\, l^* : t_c \mapsto \tilde{m}^* \,]$$

  by $\lfloor^{\mathbb{D}}|_{\mathsf{Recv}}\rceil$ where l^* is the location of the receiving process playing role $\mathtt{B}$ and t_c is the correlation key used by the process playing $\mathtt{A}$ to send to the process playing role $\mathtt{B}$. The tree t_m corresponds to the encoding of value v in the queue.

Let $\mathsf{q}@l \in \Gamma$, $t_{\mathsf{q}} = \mathbb{D}(\mathsf{q})$, and $M = \mathbb{D}|_l$, from Definition 8 of the article we have $\boxed{\mathbb{D}, [\![C]\!]}^{\Gamma} \equiv_{\mathsf{D}} S \mid S_c$ where

- $S = \left\langle \boxed{C_c|_l}^{\Gamma}, Q \mid R, M \right\rangle_l$
- $Q = \sum_{i \in I} [o_i(x_i) \texttt{ from } \underline{k.A.B}] \{ \boxed{C_i}^{\Gamma} \} \cdot t_{\mathsf{q}}$

– $R = \prod_{\mathsf{r} \in \mathbb{D}(l) \setminus \{\mathsf{q}\}} \boxed{C_c|_{\mathsf{r}}}^{\Gamma} \cdot \mathbb{D}(\mathsf{p})$

– $S_c = \prod_{l' \in \Gamma \setminus \{l\}} \left\langle \boxed{C_c|_{l'}}^{\Gamma}, \prod_{\mathsf{s} \in \mathbb{D}(l')} \boxed{C_c|_{\mathsf{s}}}^{\Gamma} \cdot t_{\mathsf{s}}, \mathbb{D}|_{l'} \right\rangle_{l'}$

In this case $\boxed{\mathbb{D}, [\![C]\!]}^{\Gamma}$ can mimic D, C applying rules $\lfloor^{\mathsf{DCC}}|_{\mathsf{Eq}}\rceil$, $\lfloor^{\mathsf{DCC}}|_{\mathsf{SPar}}\rceil$, and $\lfloor^{\mathsf{DCC}}|_{\mathsf{Recv}}\rceil$.

$$\dfrac{\dfrac{Q = \sum_{i \in I} [o_i(x_i) \ \mathtt{from} \ \underline{k.A.B}] \{ \boxed{C_i}^{\Gamma} \} \cdot t_{\mathsf{q}} \qquad j \in I \qquad t_c = \mathbf{eval}(e, t_{\mathsf{q}}) \qquad M(t_c) = (o_j, t_m) :: \tilde{m}^*}{\left\langle \boxed{C_c|_l}^{\Gamma}, Q \mid R, M \right\rangle_l \quad \to \quad \left\langle \boxed{C_c|_l}^{\Gamma}, \boxed{C_j}^{\Gamma} \cdot t_{\mathsf{q}} \triangleleft (x_j, t_m) \mid R, M[t_c \mapsto \tilde{m}^*] \right\rangle_l} \lfloor^{\mathsf{DCC}}|_{\mathsf{Recv}}\rceil}{S \mid S_c \quad \to \quad S' \mid S_c} \lfloor^{\mathsf{DCC}}|_{\mathsf{SPar}}\rceil$$

Where $S' = \left\langle \boxed{C_c|_l}^{\Gamma}, \boxed{C_j}^{\Gamma} \cdot t_{\mathsf{q}} \triangleleft (x_j, t_m) \mid R, M[t_c \mapsto \tilde{m}^*] \right\rangle_l$. Let $t'_{\mathsf{q}} = t_{\mathsf{q}} \triangleleft (x_j, t_m)$, $Q' = \boxed{C_j}^{\Gamma} \cdot t'_{\mathsf{q}}$, and $M' = M[t_c \mapsto \tilde{m}]$.

Since by Definition 8 of the article, t_c and t_m respectively result from the evaluation of the state of process q, $\mathbb{D}(\mathsf{q})$ and the encoding of value v, we have that $M' = \mathbb{D}'|_l$ and $t'_{\mathsf{q}} = \mathbb{D}'(\mathsf{q})$.

This corresponds to the compilation of the reduction D', C'', i.e,

$$\boxed{\langle\!\langle D' \rangle\!\rangle^{\Gamma'}, C''}^{\Gamma'} \equiv_{\mathsf{D}} \overbrace{\left\langle \boxed{C_c|_l}^{\Gamma'}, \overbrace{\boxed{C_j|_{\mathsf{q}}}^{\Gamma'} \cdot t'_{\mathsf{q}}}^{Q'} \mid \overbrace{\prod_{\mathsf{r} \in \mathbb{D}'(l) \setminus \{\mathsf{q}\}} \boxed{C_c|_{\mathsf{r}}}^{\Gamma'} \cdot \mathbb{D}'(\mathsf{r})}^{R}, M' \right\rangle_l}^{S'} \mid \underbrace{\prod_{l' \in \Gamma \setminus \{l\}} \left\langle \boxed{C_c|_{l'}}^{\Gamma'}, \prod_{\mathsf{s} \in D(l')} \boxed{C_c|_{\mathsf{s}}}^{\Gamma'} \cdot t_{\mathsf{s}}, \mathbb{D}'|_l \right\rangle_{l'}}_{S_c}$$

Where the changes in D' and Γ' affect only the compilation of the queue in $\mathbb{D}'|_l$ identified by t_c and the state of q; while for all other terms $\boxed{\cdot}^{\Gamma} = \boxed{\cdot}^{\Gamma'}$ and $\mathbb{D}'|_{l'} = \mathbb{D}|_{l'}$.

Case $\lfloor^{\mathsf{C}}|_{\mathsf{PStart}}\rceil$
We know that

– $[\![C]\!] \equiv_{\mathsf{C}} C_r \mid C_a \mid C_c$ where, let $\tilde{l} \colon G\langle \mathtt{A}|\tilde{\mathtt{B}}|\tilde{\mathtt{B}}\rangle \in \Gamma$

– $C_r = \mathsf{req}\ k : \mathsf{p}[\mathtt{A}] \Leftrightarrow \widetilde{l.\mathtt{B}}; C'_r$

– let $l_1.\mathtt{B}_1, \ldots, l_n.\mathtt{B}_n = \widetilde{l.\mathtt{B}}$, $C_a = \prod_{i=1}^{n} \mathsf{acc}\ k : l_i.\mathsf{q}_i[\mathtt{B}_i]; C_{\mathsf{q}_i}$

We can apply rules $\lfloor^{\mathsf{C}}|_{\mathsf{Par}}\rceil$ and $\lfloor^{\mathsf{C}}|_{\mathsf{Eq}}\rceil$ and lastly rule $\lfloor^{\mathsf{C}}|_{\mathsf{PStart}}\rceil$ such that

$$\dfrac{\begin{array}{c} i \in \{1, \ldots, n\} \qquad D \# k', \tilde{\mathsf{r}} \qquad \{\widetilde{l.\mathtt{B}}\} = \biguplus_i \{\widetilde{l_i.\mathtt{B}_i}\}_i \qquad \{\tilde{\mathsf{r}}\} = \bigcup_i \{\tilde{\mathsf{r}}_i\} \\ \delta = \mathsf{start}\ k' : \mathsf{p}[\mathtt{A}] \Leftrightarrow \widetilde{l_1.\mathsf{r}_1[\mathtt{B}_1]}, \ldots, \widetilde{l_n.\mathsf{r}_n[\mathtt{B}_n]} \qquad D, \delta \blacktriangleright D' \end{array}}{D, C_r \mid C_a \to D', C'_r[k'/k] \mid \prod_i \left(C'_{\mathsf{q}_i}[k'/k][\mathsf{r}_i/\mathsf{q}_i] \right) \mid C_a} \lfloor^{\mathsf{C}}|_{\mathsf{PStart}}\rceil$$

and

$$D, C_r \mid C_a \mid C_c \quad \xrightarrow{\tau} \quad D', C'_r[k'/k] \mid \prod_i \left(C_{\mathsf{q}_i}[k'/k][\mathsf{r}_i/\mathsf{q}_i] \right) \mid C_a \mid C_c$$

thus $C'' = C'_r[k'/k] \mid \prod_i \left(C_{\mathsf{q}_i}[k'/k][\mathsf{r}_i/\mathsf{q}_i] \right) \mid C_a \mid C_c$

We can find $\Gamma' = \Gamma, \mathbf{init}(k', (\mathsf{p}[\mathtt{A}], \widetilde{\mathsf{q}[\mathtt{B}]}), G)$ and $\Gamma' \vdash D', C'$.

Remark 1. We have two cases for, let $p@l \in \Gamma$, whether $l \in \{\tilde{l}\}$ or not. For a clearer treatment of the case we proceed considering that $l \notin \{\tilde{l}\}$ (i.e., no service process is created in the same location—service—of the requester p). The other case follows the same structure of $l \notin \{\tilde{l}\}$ although the service located at l has $\boxed{C_a|_l}^\Gamma$ as start behaviour and $\boxed{\mathbb{D}, [\![C]\!]}^\Gamma$ applies rule $\lfloor^{\mathsf{DCC}}|_{\mathsf{InStart}}\rceil$ in place of the $\lfloor^{\mathsf{DCC}}|_{\mathsf{Start}}\rceil$ for starting the DCC process located at l.

Henceforth we proceed analysing the case for $l \notin \{\tilde{l}\}$.

Let $\mathbb{D}^* = \langle\!\langle D' \rangle\!\rangle^{\Gamma'}$ and $M^* = \mathbb{D}^*|_l$ and $M_i^* = \mathbb{D}^*|_{l_i}$. Then, from Definition 8 of the article, we have

$$\boxed{\mathbb{D}^*, C''}^{\Gamma'} = \left\langle \boxed{C_c|_l}^{\Gamma'}, P'' \mid R', M^* \right\rangle_l \mid \prod_{i=1}^{n} \left\langle Q_i'', Q_i^* \mid R_{l_i}', M_i^* \right\rangle_{l_i} \mid S_c'$$

In the following, we use the abbreviation $t_{\mathsf{s}}^* = \mathbb{D}^*(\mathsf{s})$ for process s in $\mathbb{D}^*$.

- $P'' = \boxed{C_r'[k'/k]}^{\Gamma'} \cdot t_{\mathsf{p}}^*$
- $R' = \prod_{\mathsf{p}' \in \mathbb{D}^*(l) \setminus \{\mathsf{p}\}} \boxed{C_c|_{\mathsf{p}'}}^{\Gamma'} \cdot t_{\mathsf{p}'}^*$
- $Q_i'' = \mathtt{accept}(k, \mathtt{B}_i, G\langle \mathtt{A}|\tilde{\mathtt{B}}|\tilde{\mathtt{B}}\rangle); \boxed{C_{\mathsf{q}_i}}^{\Gamma'}$
- $Q_i^* = \boxed{C_{\mathsf{q}_i}[k'/k][\mathsf{r}_i/\mathsf{q}_i]}^{\Gamma'} \cdot t_{q_i}^*$
- $R_{l_i}' = \prod_{\mathsf{s} \in \mathbb{D}^*(l_i)} \boxed{C_c|_{\mathsf{s}}}^{\Gamma'} \cdot t_{\mathsf{s}}^*$
- $S_c' = \prod_{l' \in \Gamma \setminus \{l, \tilde{l}\}} \left\langle \boxed{C_c|_{l'}}^{\Gamma'}, \prod_{\mathsf{s}' \in \mathbb{D}^*(l')} \boxed{C_c|_{\mathsf{s}'}}^{\Gamma'} \cdot t_{\mathsf{s}'}^*, \mathbb{D}^*|_{l'} \right\rangle_{l'}$

From Theorem 1 of the article we can apply rule $\lfloor^{\mathbb{D}}|_{\mathsf{Start}}\rceil$ on $\mathbb{D}, [\![C]\!] \to \mathbb{D}^*, C''$ such that we know that

$$\underline{k'}(t_{\mathsf{p}}^*) = \underline{k'}(t_{\mathsf{q}_1}^*) = \ldots = \underline{k'}(t_{\mathsf{q}_n}^*) = t_{k'}$$

for some $t_{k'}$ session descriptor of session k'.

We proceed by proving that we can reduce $\boxed{\mathbb{D}, [\![C]\!]}^\Gamma \to^+ S$.

Let $t_{\mathsf{p}} = \mathbb{D}(\mathsf{p})$, $M = \mathbb{D}|_l$, and $M_i = \mathbb{D}|_{l_i}$. Then, from Definition 8 of the article, we have

$$\boxed{\mathbb{D}, [\![C]\!]}^\Gamma \equiv_{\mathsf{D}} \left\langle \boxed{C_c|_l}^\Gamma, P \mid R, M \right\rangle_l \mid \prod_{i=1}^{n} \langle Q_i, R_{l_i}, M_i \rangle_{l_i} \mid S_c$$

where

- $$\begin{aligned} P = \; & \mathtt{start}(\,k,\ (l.\mathtt{A}, \widetilde{l.\mathtt{B}})\,); \boxed{C_r'}^\Gamma \cdot t_{\mathsf{p}} = \\ = \; & \left(\begin{array}{l} \bigodot_{I \in \{\mathtt{A}, \tilde{\mathtt{B}}\}} \underline{k.I.l} = l_{\mathtt{I}}\ ; \\ \bigodot_{I \in \{\tilde{\mathtt{B}}\}} \left(\nu\rangle \underline{k.I.A}; ?@\underline{k.I.l}(\underline{k}); sync(\underline{k})\ \mathtt{from}\ \underline{k.I.A} \right); \\ \bigodot_{I \in \{\tilde{\mathtt{B}}\}} start@\underline{k.I.l}(\underline{k})\ \mathtt{to}\ \underline{k.A.I}; \boxed{C_r'}^\Gamma \end{array} \right) \cdot t_{\mathsf{p}} \end{aligned}$$
- $$Q_i = \mathtt{accept}(k, \mathtt{B}_i, G\langle \mathtt{A}|\tilde{\mathtt{B}}|\tilde{\mathtt{B}}\rangle); \boxed{C_{\mathsf{q}_i}}^\Gamma = \begin{array}{l} !(\underline{k}); \quad \bigodot_{I \in \{\mathtt{A}, \tilde{\mathtt{B}}\} \setminus \{\mathtt{B}_i\}} \nu\rangle \underline{k.I.B_i}\ ; \\ sync@\underline{k.A.l}(\underline{k})\ \mathtt{to}\ \underline{k.B_i.A}\ ; \\ start(\underline{k})\ \mathtt{from}\ \underline{k.A.B_i}\ ; \boxed{C_{\mathsf{q}_i}}^\Gamma \end{array}$$

– $R = \prod_{\mathsf{p}' \,\in\, \mathbb{D}(l) \setminus \{\mathsf{p}\}} \boxed{C_c|_{\mathsf{p}'}}^{\Gamma} \cdot t_{\mathsf{p}'}$

– $R_{l_i} = \prod_{\mathsf{s} \,\in\, \mathbb{D}(l_i)} \boxed{C_c|_{\mathsf{s}}}^{\Gamma} \cdot t_{\mathsf{s}}$

– $S_c = \prod_{l' \,\in\, \Gamma \setminus \{l, \tilde{l}\}} \left\langle \boxed{C_c|_{l'}}^{\Gamma}, \prod_{\mathsf{s}' \,\in\, \mathbb{D}(l')} \boxed{C_c|_{\mathsf{s}'}}^{\Gamma} \cdot t_{\mathsf{s}'}, \mathbb{D}|_{l'} \right\rangle_{l'}$

$\boxed{\mathbb{D}, [\![C]\!]}^{\Gamma}$ can mimic D, C with the following sequence of reductions. Note that we make use of renaming on (acc) terms in $Q_1, \dots, Q_n$ and variable renaming on P (as of Definition 12) to align the evolution of $\boxed{\mathbb{D}, [\![C]\!]}^{\Gamma}$ with the evolution of D, C, in which k has been renamed with the fresh name k'. Since the renamed DCC network and the original one are bisimilar, as per Lemma 14, we can proceed to prove our results on the original DCC network using the DCC renamed network as a proxy.

Therefore we take $S_0^* \sim \boxed{\mathbb{D}, [\![C]\!]}^{\Gamma}$

$$S_0^* = \left\langle \boxed{C_c|_l}^{\Gamma}, P[\underline{k'}/\underline{k}] \mid R, M \right\rangle_l \mid \prod_{i=1}^{n} \langle Q_i[\underline{k'}/\underline{k}], R_{l_i}, M_i \rangle_{l_i} \mid S_c$$

$$S_0^* \rightarrow \left.\begin{array}{l} \left.\xrightarrow{\lfloor \mathsf{DCC}|_{\mathsf{SEq}} \rceil \;\; \lfloor \mathsf{DCC}|_{\mathsf{SPar}} \rceil \;\; \lfloor \mathsf{DCC}|_{\mathsf{PPar}} \rceil \;\; \lfloor \mathsf{DCC}|_{\mathsf{Assign}} \rceil}\right\} (1)\ n+1 \text{ times} \\ \left.\begin{array}{l} (2.1) \xrightarrow{\lfloor \mathsf{DCC}|_{\mathsf{SEq}} \rceil \;\; \lfloor \mathsf{DCC}|_{\mathsf{SPar}} \rceil \;\; \lfloor \mathsf{DCC}|_{\mathsf{Newque}} \rceil} \\ (2.2) \xrightarrow{\lfloor \mathsf{DCC}|_{\mathsf{SEq}} \rceil \;\; \lfloor \mathsf{DCC}|_{\mathsf{SPar}} \rceil \;\; \lfloor \mathsf{DCC}|_{\mathsf{Start}} \rceil} \\ \left.(2.3) \xrightarrow{\lfloor \mathsf{DCC}|_{\mathsf{SEq}} \rceil \;\; \lfloor \mathsf{DCC}|_{\mathsf{SPar}} \rceil \;\; \lfloor \mathsf{DCC}|_{\mathsf{Newque}} \rceil}\right\} n \text{ times} \\ (2.4) \xrightarrow{\lfloor \mathsf{DCC}|_{\mathsf{SEq}} \rceil \;\; \lfloor \mathsf{DCC}|_{\mathsf{SPar}} \rceil \;\; \lfloor \mathsf{DCC}|_{\mathsf{Send}} \rceil} \\ (2.5) \xrightarrow{\lfloor \mathsf{DCC}|_{\mathsf{SEq}} \rceil \;\; \lfloor \mathsf{DCC}|_{\mathsf{SPar}} \rceil \;\; \lfloor \mathsf{DCC}|_{\mathsf{Recv}} \rceil} \end{array}\right\} (2)\ \text{n times} \\ \left.\begin{array}{l} (3.1) \xrightarrow{\lfloor \mathsf{DCC}|_{\mathsf{SEq}} \rceil \;\; \lfloor \mathsf{DCC}|_{\mathsf{SPar}} \rceil \;\; \lfloor \mathsf{DCC}|_{\mathsf{Send}} \rceil} \\ (3.2) \xrightarrow{\lfloor \mathsf{DCC}|_{\mathsf{SEq}} \rceil \;\; \lfloor \mathsf{DCC}|_{\mathsf{SPar}} \rceil \;\; \lfloor \mathsf{DCC}|_{\mathsf{Recv}} \rceil} \end{array}\right\} (3)\ n \text{ times} \end{array}\right. \rightarrow S_1^*$$

We briefly comment on the numbered transitions.

– In (1) $P[k'/k]$ proceeds to store (for $n+1$ times, l plus $l_i, i \in \{1, \dots, n\}$) the locations of all roles under $\underline{k'}$.

– In (2), for each location $l_i, i \in \{1, \dots, n\}$ (for each service process):

  * P creates its receiving queue for the service process (2.1);
  * in (2.2) P synchronises with the service at location l_i starting ($\lfloor \mathsf{DCC}|_{\mathsf{Start}} \rceil$) a new service process;
  * in (2.3) the service process creates its own queues for all other roles in the session (hence n times);
  * in (2.4) the service process sends the correlation values to P;
  * finally P receives the message in (2.5).

– In (3) for each service process (n times) (3.1) the starter sends a message to the service process to start the session and (3.2) the addressee receives it.

Finally we have

$$S_1^* = \left\langle \boxed{C_c|_l}^{\Gamma} \mid P' \mid R, M' \right\rangle_l \mid \prod_{i=1}^{n} \langle Q_i[\underline{k'}/\underline{k}], Q_i' \mid R_{l_i}, M_i' \rangle_{l_i} \mid S_c$$

where

- $P' = \boxed{C_r'}^{\Gamma} [\underline{k'}/\underline{k}] \cdot t_p'$, and
- $Q_i' = \boxed{\mathsf{C}_{\mathsf{q}_i}}^{\Gamma} [\underline{k'}/\underline{k}] \cdot t_{k'}$

From the transitions presented above we know that there exists $t_{k'}'$ such that $t_{\mathsf{p}}' = t_{\mathsf{p}} \lhd (\underline{k'}, t_{k'}')$, where $t_{k'}'$ is a session descriptor for session k' (i.e., it contains all the locations and correlation keys used by the processes in session k'). In this case, we take $t_k' = t_{k'}$ obtained from the derivation $\mathbb{D}, C \to \mathbb{D}^*, C'$.

Similarly, M' and $M_1', \ldots, M_n'$ contain the necessary (empty) queues to support communication in session k'.

$$M' = M[\underline{k'.B_1.A}(t_{k'}) \mapsto \varepsilon] \ \ldots \ [\underline{k'.B_n.A}(t_{k'}) \mapsto \varepsilon]$$

and ($\varnothing$ being a totally undefined function on $Val \rightharpoonup \mathcal{M}$)

$$M_i = \varnothing \begin{array}{l} [\underline{k'.A.B_i}(t_k) \mapsto \varepsilon][\underline{k'.B_1.B_i}(t_k) \mapsto \varepsilon] \ \ldots \ [\underline{k'.B_{i-1}.B_i}(t_k) \mapsto \varepsilon] \ \ldots \\ \ldots \ [\underline{k'.B_{i+1}.B_i}(t_k) \mapsto \varepsilon] \ \ldots \ [\underline{k'.B_n.B_i}(t_k) \mapsto \varepsilon] \end{array}$$

We proceed with the proof taking $S \sim S_1^*$ as S is simply the renaming of $\underline{k'}$ to $\underline{k}$ on start behaviour $Q_i, i \in \{1, \ldots, n\}$ (trivially $Q_i[\underline{k'}/\underline{k}][\underline{k}/\underline{k'}] = Q_i$)

$$S = \left\langle \boxed{C_c|_l}^{\Gamma} \mid P' \mid R, M' \right\rangle_l \mid \prod_{i=1}^{n} \langle Q_i, Q_i' \mid R_{l_i}, M_i' \rangle_{l_i} \mid S_c$$

We now proceed to prove that $\boxed{\mathbb{D}, [\![C]\!]}^{\Gamma} \to^{+} \boxed{\mathbb{D}^*, C''}^{\Gamma'}$, i.e. that $\boxed{\mathbb{D}^*, C''}^{\Gamma'} = S$ with $\Gamma' \vdash D', C'$.

We prove that

$$\overbrace{\left\langle \boxed{C_c|_l}^{\Gamma'}, P'' \mid R', M^* \right\rangle_l \mid \prod_{i=1}^{n} \left\langle Q_i'', Q_i^* \mid R_{l_i}', M_i^* \right\rangle_{l_i} \mid S_c'}^{\boxed{\mathbb{D}^*, C''}^{\Gamma'}} = \overbrace{\left\langle \boxed{C_c|_l}^{\Gamma} \mid P' \mid R, M' \right\rangle_l \mid \prod_{i=1}^{n} \langle Q_i, Q_i' \mid R_{l_i}, M_i' \rangle_{l_i} \mid S_c}^{S}$$

- M^* and M' are equal and similarly M_i^* and M_i are pair-wise equal by construction and rule $\lfloor^{\mathbb{D}}|_{\mathsf{Start}}\rceil$;
- $\boxed{C_c|_l}^{\Gamma} = \boxed{C_c|_l}^{\Gamma'}$ as $\Gamma|_{\mathtt{locs}} = \Gamma'|_{\mathtt{locs}}$ by construction;
- $P'' = P'$ is proved by

  $$\boxed{C_r'[k'/k]}^{\Gamma'} \cdot t_{\mathsf{p}}^* = \boxed{C_r'}^{\Gamma} [\underline{k'}/\underline{k}] \cdot t_p'$$

  which holds as

  i) $\boxed{C_r'[k'/k]}^{\Gamma'} = \boxed{C_r'}^{\Gamma} [\underline{k'}/\underline{k}]$ since

    (a) Γ' does not contain any new process used in C_r';

    (b) by renaming, and Lemma 14.

  ii) $t_{\mathsf{p}}^* = t_{\mathsf{p}}'$ by construction and rule $\lfloor^{\mathbb{D}}|_{\mathsf{Start}}\rceil$.

- $Q_i'^* = Q_i'$ proved by

$$\boxed{C_{\mathsf{q}_i}[k'/k][\mathsf{r}_i/\mathsf{q}_i]}^{\Gamma'} \cdot t^*_{q_i} = \boxed{C_{\mathsf{q}_i}}^{\Gamma}[\underline{k}'/\underline{k}] \cdot t_{k'}$$

whose proof of equivalence follows that of $P'' = P'$, except that Γ' contains the location of the process (r_i) used in $C_{\mathsf{q}_i}[k'/k][\mathsf{r}_i/\mathsf{q}_i]$.

- $Q_i'' = Q_i$ proved by

$$\mathtt{accept}(k, \mathtt{B}_i, G\langle \mathtt{A}|\tilde{\mathtt{B}}|\tilde{\mathtt{B}}\rangle); \boxed{C_{\mathsf{q}_i}}^{\Gamma'} = \mathtt{accept}(k, \mathtt{B}_i, G\langle \mathtt{A}|\tilde{\mathtt{B}}|\tilde{\mathtt{B}}\rangle); \boxed{C_{\mathsf{q}_i}}^{\Gamma}$$

which holds as $\boxed{C_{\mathsf{q}_i}}^{\Gamma'} = \boxed{C_{\mathsf{q}_i}}^{\Gamma}$ because Γ and Γ' contain the same service typings.

- $R' = R$ is proved by

$$\prod_{\mathsf{p}' \in \mathbb{D}^*(l) \setminus \{\mathsf{p}\}} \boxed{C_c|_{\mathsf{p}'}}^{\Gamma'} \cdot t^*_{\mathsf{p}'} = \prod_{\mathsf{p}' \in \mathbb{D}(l) \setminus \{\mathsf{p}\}} \boxed{C_c|_{\mathsf{p}'}}^{\Gamma} \cdot t_{\mathsf{p}'}$$

for which

i) $\boxed{C_c|_{\mathsf{p}'}}^{\Gamma'} = \boxed{C_c|_{\mathsf{p}'}}^{\Gamma}$ as Γ' does not contain any new process used in C_c.

ii) $t^*_{\mathsf{p}'} = t_{\mathsf{p}'}$ unchanged by the reductions of $\mathbb{D}, C$ and $\boxed{\mathbb{D}, [\![C]\!]}^{\Gamma}$.

- $R'_{l_i} = R_{l_i}$ whose proof follows that of $R' = R$.

- $S'_c = S_c$ following the proof of $\boxed{C_c|_l}^{\Gamma} = \boxed{C_c|_l}^{\Gamma'}$ and $R'_{l_i} = R_{l_i}$.

Case $\lfloor^{\mathsf{C}}|_{\mathsf{Start}}\rceil$

While the original FC program reduces applying rule $\lfloor^{\mathsf{C}}|_{\mathsf{Start}}\rceil$, the endpoint projection $D, [\![C]\!]$ will mimic it applying rule $\lfloor^{\mathsf{C}}|_{\mathsf{PStart}}\rceil$, as per Theorem 2 of the article. Hence, to prove this case, we can follow the same proof of case $\lfloor^{\mathsf{C}}|_{\mathsf{PStart}}\rceil$.

Case $\lfloor^{\mathsf{C}}|_{\mathsf{Cond}}\rceil$

We have $[\![C]\!] = C_{\mathsf{p}} \mid C_c$ where $C_{\mathsf{p}} = \mathtt{if}\ \mathsf{p}.e\ \{C_1\}\ \mathtt{else}\ \{C_2\}$. Let $\mathsf{p}@l \in \Gamma$ and

- $t_{\mathsf{p}} = \mathbb{D}(\mathsf{p})$;
- $P = \mathtt{if}\ e\ \{\boxed{C_1}^{\Gamma}\}\ \mathtt{else}\ \{\boxed{C_2}^{\Gamma}\} \cdot t_{\mathsf{p}}$;
- $R = \prod_{\mathsf{r} \in \mathbb{D}(l) \setminus \{\mathsf{p}\}} \boxed{C_c|_{\mathsf{r}}}^{\Gamma} \cdot t_{\mathsf{r}}$
- $S_c = \prod_{l' \in \Gamma \setminus \{l\}} \left\langle \boxed{C_c|_{l'}}^{\Gamma}, \prod_{\mathsf{r} \in \mathbb{D}(l')} \boxed{C_c|_{\mathsf{r}}}^{\Gamma} \cdot t_{\mathsf{r}}, \mathbb{D}|_{l'} \right\rangle_{l'}$

Let $M = \mathbb{D}|_l$. Then, from Definition 8 of the article, we have

$$\boxed{\mathbb{D}, [\![C]\!]}^{\Gamma} \equiv_{\mathsf{D}} \left\langle \boxed{C_c|_l}^{\Gamma}, P \mid R, M \right\rangle_l \mid S_c$$

we reduce $\mathbb{D}, [\![C]\!]$ applying rules $\lfloor^{\mathsf{C}}|_{\mathsf{Par}}\rceil$, $\lfloor^{\mathsf{C}}|_{\mathsf{Eq}}\rceil$ and lastly rule $\lfloor^{\mathsf{C}}|_{\mathsf{Cond}}\rceil$. We analyse only the case for $\mathbf{eval}(e, t_{\mathsf{p}}) = \mathtt{true}$ as the other case for $\mathbf{eval}(e, t_{\mathsf{p}}) = \mathtt{false}$ follows the same structure.

$$\mathbb{D}, [\![C]\!] \quad \xrightarrow{\tau} \quad \mathbb{D}', C''$$

and $C'' = C_1 \mid C_c$ and $\mathbb{D}' = \mathbb{D}$ by the definition of $\lfloor^{\mathsf{C}}|_{\mathsf{Cond}}\rceil$. We can choose $\Gamma = \Gamma'$, for which it holds that $\Gamma \vdash D', C'$.

From Definition 8 of the article we have

$$\boxed{\mathbb{D}', C''}^{\Gamma'} = \boxed{\mathbb{D}, C''}^{\Gamma} = \left\langle \boxed{C_c|_l}^{\Gamma}, \boxed{C_1}^{\Gamma} \cdot t_{\mathsf{p}} \mid R, M \right\rangle_l \mid S_c$$

$\boxed{\mathbb{D}, [\![C]\!]}^{\Gamma}$ can mimic D, C applying rules $\lfloor^{\mathsf{DCC}}|_{\mathsf{Eq}}\rceil$, $\lfloor^{\mathsf{DCC}}|_{\mathsf{SPar}}\rceil$, $\lfloor^{\mathsf{DCC}}|_{\mathsf{PPar}}\rceil$, and lastly $\lfloor^{\mathsf{DCC}}|_{\mathsf{Cond}}\rceil$ for which

$$\boxed{\mathbb{D}, [\![C]\!]}^{\Gamma} \to \left\langle \boxed{C_c|_l}^{\Gamma}, \boxed{C_1}^{\Gamma} \cdot t_{\mathsf{p}} \mid R, M \right\rangle_l \mid S_c$$

Case $\lfloor^{\mathsf{C}}|_{\mathsf{Ctx}}\rceil$
The thesis follows from the induction hypothesis as D, C applies rule $\lfloor^{\mathsf{C}}|_{\mathsf{Ctx}}\rceil$ and $\boxed{\mathbb{D}, [\![C]\!]}^{\Gamma}$ can mimic it with rule $\lfloor^{\mathsf{DCC}}|_{\mathsf{Ctx}}\rceil$.

Case $\lfloor^{\mathsf{C}}|_{\mathsf{Par}}\rceil$
The thesis follows from the induction hypothesis.

Case $\lfloor^{\mathsf{C}}|_{\mathsf{Eq}}\rceil$
The thesis follows from the induction hypothesis. Starting from any configuration of D, C, $\boxed{\mathbb{D}, [\![C]\!]}^{\Gamma}$ can always mimic the evolution of D, C when it applies rule $\lfloor^{\mathsf{C}}|_{\mathsf{Eq}}\rceil$: in both cases that $\mathcal{R} = \equiv$ or $\mathcal{R} = \simeq_{\mathsf{C}}$, $\boxed{\mathbb{D}, [\![C]\!]}^{\Gamma}$ can apply $\lfloor^{\mathsf{DCC}}|_{\mathsf{Eq}}\rceil$, $\lfloor^{\mathsf{DCC}}|_{\mathsf{SPar}}\rceil$, and $\lfloor^{\mathsf{DCC}}|_{\mathsf{PPar}}\rceil$ to mimic D, C.

□

Before proceeding with the proof of (Soundness) of Theorem 3 of the article, we extend the semantics of DCC by annotating its transitions with the variable paths (of the kind $x = \underline{x.y.z}$) on which DCC operations execute. We range over DCC transition labels with λ.

$$\lambda ::= x \mid v\rangle x \mid ?(x) \mid o \;\mathtt{from}\; x \mid o \;\mathtt{to}\; x \mid \tau$$

We report in Figure 12 the annotated semantics of DCC.

We also introduce two operators on sequences of DCC transition labels. Let $\lambda, \tilde{\lambda}$ be a sequence of DCC labels, the filtering of $\lambda, \tilde{\lambda}$ on k , written $(\lambda, \tilde{\lambda})\big|_k$ is defined as

$$(\lambda, \tilde{\lambda})\big|_k = \begin{cases} \lambda, (\tilde{\lambda}\big|_k) & \text{if } \lambda \in \left\{ \begin{array}{c} \underline{k.x.y},\ v\rangle\underline{k.x.y},\ ?(\underline{k}), \\ \mathit{sync}\;\mathtt{from}\;\underline{k.x.y},\ \mathit{sync}@\underline{k.x.y}, \\ \mathit{start}\;\mathtt{from}\;\underline{k.x.y},\ \mathit{start}@\underline{k.x.y} \end{array} \right\} \\ \tilde{\lambda}\big|_k & \text{otherwise} \end{cases}$$

Let $\lambda_1, \tilde{\lambda}_1$ and $\lambda_2, \tilde{\lambda}_2$ be two sequences of DCC labels, the complement of $\lambda_1, \tilde{\lambda}_1$ on $\lambda_2, \tilde{\lambda}_2$, written $(\lambda_1, \tilde{\lambda}_1) \setminus (\lambda_2, \tilde{\lambda}_2)$ is defined as

$$(\lambda_1, \varepsilon) \setminus (\lambda_2, \tilde{\lambda}_2) = \begin{cases} \varepsilon & \text{if } \lambda_1 = \lambda_2 \\ \lambda_1 & \text{otherwise} \end{cases}$$

$$(\lambda_1, \tilde{\lambda}_1) \setminus (\lambda_2, \tilde{\lambda}_2) = \begin{cases} \tilde{\lambda}_1 \setminus \tilde{\lambda}_2 & \text{if } \lambda_1 = \lambda_2 \\ \lambda_1, (\tilde{\lambda}_1 \setminus (\lambda_2, \tilde{\lambda}_2)) & \text{otherwise} \end{cases}$$

Below we state Lemma 15 that proves that, given a DCC system S and a sequence of reductions $\tilde{\lambda}$ for which $S \xrightarrow{\tilde{\lambda}} S'$, if the first action is the initiation of a session k, then we can reorder the execution of the subsequent actions in $\tilde{\lambda}$ such that we first execute all transitions related to the start of k and then all the remaining actions, obtaining the same final system S'.

Lemma 15 (DCC Start Permutation)**.** Let S be a composition of DCC services such that $S \xrightarrow{\tilde{\lambda}} S'$ where $\tilde{\lambda} = \underline{k.C.l}, \tilde{\lambda}'$, then let $\tilde{\lambda}_k = \tilde{\lambda}\big|_k$ and $\tilde{\lambda}_* = \tilde{\lambda} \setminus \tilde{\lambda}_k$ we have $S \xrightarrow{\tilde{\lambda}_k} S_1$ and $S_1 \xrightarrow{\tilde{\lambda}_*} S'$.

$$\frac{t' = \mathbf{eval}(x,t)}{x = e\,; B \cdot t \quad \xrightarrow{x} \quad B \cdot t \triangleleft (\,x, t'\,)} \;\lfloor\mathsf{DCC}|_{\mathsf{Assign}}\rceil \qquad \frac{B \cdot t \xrightarrow{\lambda} B' \cdot t'}{\mathtt{def}\; X = B_1 \;\mathtt{in}\; B \cdot t \quad \xrightarrow{\lambda} \quad \mathtt{def}\; X = B_1 \;\mathtt{in}\; B' \cdot t'} \;\lfloor\mathsf{DCC}|_{\mathsf{Ctx}}\rceil$$

$$\frac{i = 1 \text{ if } \mathbf{eval}(e,t) = \text{true}, i = 2 \text{ otherwise}}{\mathtt{if}\; e\; \{B_1\}\; \mathtt{else}\; \{B_2\} \cdot t \quad \xrightarrow{\tau} \quad B_i \cdot t} \;\lfloor\mathsf{DCC}|_{\mathsf{Cond}}\rceil \qquad \frac{P \equiv_{\mathsf{D}} P_1 \mid P_2 \quad P_1 \xrightarrow{\lambda} P_1' \quad P_1' \mid P_2 \equiv_{\mathsf{D}} P'}{\langle \mathfrak{B},\, P,\, M \rangle_l \quad \xrightarrow{\lambda} \quad \langle \mathfrak{B},\, P',\, M \rangle_l} \;\lfloor\mathsf{DCC}|_{\mathsf{PEq}}\rceil$$

$$\frac{B = \nu\rangle x; B \quad t_c \notin \mathbf{dom}(M) \quad M' = M[t_c \mapsto \varepsilon]}{\langle \mathfrak{B},\, B \cdot t \mid P,\, M \rangle_l \quad \xrightarrow{\nu\rangle x} \quad \langle \mathfrak{B},\, B \cdot t \triangleleft (\,x, t_c\,) \mid P,\, M' \rangle_l} \;\lfloor\mathsf{DCC}|_{\mathsf{Newque}}\rceil$$

$$\frac{\begin{array}{c} B \in \{\; o_j(x_j)\; \mathtt{from}\; e; B_j\;,\; \sum_{i \in I} [o_i(x_i)\; \mathtt{from}\; e]\, \{B_i\}\;\} \\ j \in I \qquad t_c = \mathbf{eval}(e,t) \qquad M(t_c) = (o_j, t_m) :: \tilde{m} \end{array}}{\langle \mathfrak{B},\, B \cdot t \mid P,\, M \rangle_l \quad \xrightarrow{o_j\;\mathtt{from}\;e} \quad \big\langle \mathfrak{B},\, B_j \cdot t \triangleleft (\,x_j, t_m\,) \mid P,\, M[t_c \mapsto \tilde{m}] \big\rangle_l} \;\lfloor\mathsf{DCC}|_{\mathsf{Recv}}\rceil$$

$$\frac{\begin{array}{c} B = o@e_1(e_2)\; \mathtt{to}\; e_3; B' \qquad \mathbf{eval}(e_1,t) = l \\ \mathbf{eval}(e_3,t) = t_c \qquad \mathbf{eval}(e_2,t) = t_m \qquad t_c \in \mathbf{dom}(M) \end{array}}{\langle \mathfrak{B},\, B \cdot t \mid P, M \rangle_l \quad \xrightarrow{o\;\mathtt{to}\;e_3} \quad \langle \mathfrak{B},\, B' \cdot t \mid P, M[t_c \mapsto M(t_c) :: (o, t_m)] \rangle_l} \;\lfloor\mathsf{DCC}|_{\mathsf{InSend}}\rceil$$

$$\frac{B = ?@e_1(e_2); B'' \quad Q = B' \cdot \varnothing \triangleleft (\,x, \mathbf{eval}(e_2,t)\,)}{\langle !(x); B',\, B \cdot t \mid P,\, M \rangle_l \quad \xrightarrow{?(e_2)} \quad \langle !(x); B',\, Q \mid B'' \cdot t \mid P,\, M \rangle_l} \;\lfloor\mathsf{DCC}|_{\mathsf{InStart}}\rceil$$

$$\frac{\begin{array}{c} B = o@e_1(e_2)\; \mathtt{to}\; e_3; B'' \qquad \mathbf{eval}(e_1,t) = l' \qquad \mathbf{eval}(e_3,t) = t_c \\ \mathbf{eval}(e_2,t) = t_m \qquad t_c \in \mathbf{dom}(M') \qquad M'' = M'[t_c \mapsto M'(t_c) :: (o, t_m)] \end{array}}{\langle \mathfrak{B}, B \cdot t \mid P,\, M \rangle_l \mid \langle \mathfrak{B}', P',\, M' \rangle_{l'} \quad \xrightarrow{o\;\mathtt{to}\;e_3} \quad \langle \mathfrak{B}, B'' \cdot t \mid P,\, M \rangle_l \mid \langle \mathfrak{B}', P', M'' \rangle_{l'}} \;\lfloor\mathsf{DCC}|_{\mathsf{Send}}\rceil$$

$$\frac{B = ?@e_1(e_2); B'' \quad \mathfrak{B}' = !(x); B' \quad \mathbf{eval}(e_1,t) = l' \quad Q = B' \cdot \varnothing \triangleleft (\,x, \mathbf{eval}(e_2,t)\,)}{\langle \mathfrak{B},\, B \cdot t \mid P, M \rangle_l \mid \langle \mathfrak{B}',\, P',\, M' \rangle_{l'} \quad \xrightarrow{?(e_2)} \quad \langle \mathfrak{B},\, B'' \cdot t \mid P,\, M \rangle_l \mid \langle \mathfrak{B}', Q \mid P',\, M' \rangle_{l'}} \;\lfloor\mathsf{DCC}|_{\mathsf{Start}}\rceil$$

$$\frac{S \xrightarrow{\lambda} S'}{S \mid S_1 \quad \xrightarrow{\lambda} \quad S' \mid S_1} \;\lfloor\mathsf{DCC}|_{\mathsf{SPar}}\rceil \qquad \frac{S \equiv_{\mathsf{D}} S_1 \quad S_1 \xrightarrow{\lambda} S_1' \quad S_1' \equiv_{\mathsf{D}} S'}{S \quad \xrightarrow{\lambda} \quad S'} \;\lfloor\mathsf{DCC}|_{\mathsf{SEq}}\rceil$$

Figure 12. Dynamic Correlation Calculus, annotated semantics.

Proof Sketch The proof is by induction on the length of $\tilde{\lambda}$. The main intuition is that, since the first action is the start of the new session k, all other actions in $\tilde{\lambda}'$ either are related to the initiation of k or do not affect it. Hence, we can reorder the execution of actions in $\tilde{\lambda}$ such that first we execute all actions regarding the start of the session[3] contained in $\tilde{\lambda}_k$ and then all the other actions in $\tilde{\lambda}_*$. □

Next we state Lemma 16 that proves that, given

- a well-typed FC endpoint choreography D,C
- its DCC compilation S
- the DCC system S' that results from an arbitrary number of steps of reduction belonging to the start of a session k in S

we can execute the remaining steps of reduction in S' to complete the start of session k, obtaining the final system S'' and prove that S'' is the same DCC system as the one obtained from the compilation of D',C', the reductum of the source FC choreography D,C after the step of reduction to start session k.

Lemma 16 (DCC Start Completion)**.** Let $\Gamma \vdash D,C$, C a endpoint choreography

$$C = \mathtt{req}\,k : \mathtt{p}[\mathtt{A}] \mathrel{\triangleleft\!\!\!-\!\!\!\triangleright} l_1.[\mathtt{B}_1],\ldots,l_n.[\mathtt{B}_n];C_r \mid \prod_{i=1}^{n} \mathtt{acc}\,k : l_i.\mathtt{q}_i[\mathtt{B}_i];C_{\mathtt{q}_i}$$

and $\boxed{\langle\!\langle D\rangle\!\rangle^{\Gamma},C}^{\Gamma} = S$ such that $S \xrightarrow{\tilde{\lambda}} S'$ where $\tilde{\lambda}\big|_k = \tilde{\lambda}$ then *i*) $S' \xrightarrow{\tilde{\lambda}'} S''$, *ii*) $D,C \to D',C'$, and *iii*) there exists some Γ' s.t. $\Gamma' \vdash D',C'$ and $\boxed{\langle\!\langle D'\rangle\!\rangle^{\Gamma'},C'}^{\Gamma'} = S''$.

Proof Proof by case analysis on the length of $\tilde{\lambda}$.

Let $\mathtt{p}@l \in \Gamma$. To proceed, we have two subcases whether $l \in \{l_1,\ldots,l_n\}$, i.e., whether one of the service processes is at the same location of $\mathtt{p}$. Since the subcases follow the same structure, we detail only the proof for $l \notin \{l_1,\ldots,l_n\}$ which allows for a uniform treatment. In the other case, *i*) we should account for transitions on the same service of $\mathtt{p}$ with rules $\lfloor^{\mathsf{DCC}}|_{\mathsf{InStart}}\rceil$ and $\lfloor^{\mathsf{DCC}}|_{\mathsf{InSend}}\rceil$ and *ii*) we would have a newly created process in parallel with $\mathtt{p}$ in D,C and in the correspondent DCC system S''.

Provided n is the number of service processes involved in the start of the session k, from Definition 8 of the article we can count the number of transitions needed to complete the start of a session. Indeed, given a D,C with

$$C = \mathtt{req}\,k : \mathtt{p}[\mathtt{A}] \mathrel{\triangleleft\!\!\!-\!\!\!\triangleright} l_1.[\mathtt{B}_1],\ldots,l_n.[\mathtt{B}_n];C_r \mid \prod_{i=1}^{n} \mathtt{acc}\,k : l_i.\mathtt{q}_i[\mathtt{B}_i];C_{\mathtt{q}_i}$$

and $\boxed{\langle\!\langle D\rangle\!\rangle^{\Gamma},C}^{\Gamma} = S$ then we can write the sequence of transitions of the compiled DCC system

$$S \overbrace{\xrightarrow{\underline{k.I.I}}}^{(1)} \underbrace{\overbrace{\xrightarrow{\nu\rangle\underline{k.A.I}}}^{(2.1)} \overbrace{\xrightarrow{?(\underline{k})}}^{(2.2)} \overbrace{\xrightarrow{\nu\rangle\underline{k.I.I'}}}^{(2.3)} \overbrace{\xrightarrow{sync@\underline{k.I'.A}}}^{(2.4)} \overbrace{\xrightarrow{sync\ \mathtt{from}\ \underline{k.I'.A}}}^{(2.5)}}_{(2)} \underbrace{\overbrace{\xrightarrow{start@\underline{k.I'.A}}}^{(3.1)} \overbrace{\xrightarrow{start\ \mathtt{from}\ \underline{k.I'.A}}}^{(3.2)}}_{(3)} S''$$

and count the number of all the transitions to complete the start, let it be m, as the sum of:

(1) n+1 times, for $\underline{I} \in \{\mathtt{A},\tilde{\mathtt{B}}\}$, with last Rule $\lfloor^{\mathsf{DCC}}|_{\mathsf{Assign}}\rceil$;

[3]Note, this does not imply nor require that λ contains all actions needed to start session k.

② n times, for $\underline{I} \in \tilde{\mathtt{B}}$:

- (2.1) reduces with last applied rule $\lfloor^{\mathsf{DCC}}|_{\mathsf{Newque}}\rceil$;
- (2.2) reduces with last applied rule $\lfloor^{\mathsf{DCC}}|_{\mathsf{Start}}\rceil$;
- (2.3) n times for $\underline{I'} \in \{\mathtt{A},\tilde{\mathtt{B}}\} \setminus \{\underline{I}\}$, reduces with last applied rule $\lfloor^{\mathsf{DCC}}|_{\mathsf{Newque}}\rceil$;
- (2.4) reduces with last applied rule $\lfloor^{\mathsf{DCC}}|_{\mathsf{Send}}\rceil$;
- (2.5) reduces with last applied rule $\lfloor^{\mathsf{DCC}}|_{\mathsf{Recv}}\rceil$;

③ n times, for $\underline{I} \in \tilde{\mathtt{B}}$:

- (3.1) reduces with last applied rule $\lfloor^{\mathsf{DCC}}|_{\mathsf{Send}}\rceil$;
- (3.2) reduces with last applied rule $\lfloor^{\mathsf{DCC}}|_{\mathsf{Recv}}\rceil$;

and $m = n^2 + 7n + 1$. We proceed unfolding the proof on the length of $\tilde{\lambda}$.

Case $|\{\tilde{\lambda}\}| = 1$

Since the cardinality of $\tilde{\lambda}$ is one and that from the premises we know that $\tilde{\lambda}$ contains only transitions belonging to the start of session k, we can infer that $\tilde{\lambda} = \underline{k.C.l}$ where $\mathtt{C} \in \{\mathtt{A},\tilde{\mathtt{B}}\}$.

To prove the thesis we let S' do all the remaining transitions to start the session and show that D,C can mimic it. Let $\widetilde{l.\mathtt{B}} = l_1.\mathtt{B}_1,\ldots,l_n.\mathtt{B}_n$ and $\tilde{l}\colon G\langle \mathtt{A}|\tilde{\mathtt{B}}|\tilde{\mathtt{B}}\rangle \in \Gamma$.

Let $\mathbb{D} = \langle\!\langle D\rangle\!\rangle^{\Gamma}$, $M = \mathbb{D}(l)$, $M_i = \mathbb{D}(l_i)$, and $t_{\mathsf{p}} = \mathbb{D}(\mathsf{p})$. Then, from Definition 8 of the article and Theorem 1 of the article, we have

$$\boxed{\langle\!\langle D\rangle\!\rangle^{\Gamma}, C}^{\Gamma} \equiv_{\mathtt{D}} \left\langle \boxed{C_c|_l}^{\Gamma}, P \mid R, M\right\rangle_l \mid \prod_{i=1}^{n} \langle Q_i, R_{l_i}, M_i\rangle_{l_i} \mid S_c$$

where

$$P = \ \mathtt{start}(\,k,\ (l.\mathtt{A},\widetilde{l.\mathtt{B}})\,); \boxed{C_r}^{\Gamma} \cdot t_{\mathsf{p}} =$$

$$- \quad = \left(\begin{array}{l} \bigodot_{\underline{I} \in \{\mathtt{A},\tilde{\mathtt{B}}\}} \underline{k.I.l} = l_{\mathtt{I}}\ ; \\ \bigodot_{\underline{I} \in \{\tilde{\mathtt{B}}\}} \Big(\nu\rangle \underline{k.I.A}; ?@\underline{k.I.l}(\underline{k}); sync(\underline{k})\ \mathtt{from}\ \underline{k.I.A}\Big); \\ \bigodot_{\underline{I} \in \{\tilde{\mathtt{B}}\}} start@\underline{k.I.l}(\underline{k})\ \mathtt{to}\ \underline{k.A.I}; \end{array} \right); \boxed{C_r}^{\Gamma} \cdot t_{\mathsf{p}}$$

- $Q_i = \mathtt{accept}(k,\mathtt{B}_i,G\langle \mathtt{A}|\tilde{\mathtt{B}}|\tilde{\mathtt{B}}\rangle); \boxed{C_{\mathsf{q}_i}}^{\Gamma} = \begin{array}{l} !(\underline{k}); \quad \bigodot_{\underline{I} \in \{\mathtt{A},\tilde{\mathtt{B}}\} \setminus \{\mathtt{B}_i\}} \nu\rangle \underline{k.I.B_i}\ ; \\ sync@\underline{k.A.l}(\underline{k})\ \mathtt{to}\ \underline{k.B_i.A}\ ; \\ start(\underline{k})\ \mathtt{from}\ \underline{k.A.B_i}\ ;\ \boxed{C_{\mathsf{q}_i}}^{\Gamma} \end{array}$

- $R = \prod_{\mathsf{p}' \,\in\, \mathbb{D}(l) \setminus \{\mathsf{p}\}} \boxed{C_c|_{\mathsf{p}'}}^{\Gamma} \cdot t_{\mathsf{p}'}$

- $R_{l_i} = \prod_{\mathsf{s} \,\in\, \mathbb{D}(l_i)} \boxed{C_c|_{\mathsf{s}}}^{\Gamma} \cdot t_{\mathsf{s}}$

- $S_c = \prod_{l' \,\in\, \Gamma \setminus \{l,\tilde{l}\}} \left\langle \boxed{C_c|_{l'}}^{\Gamma}, \prod_{\mathsf{s}' \,\in\, D(l')} \boxed{C_c|_{\mathsf{s}'}}^{\Gamma} \cdot t_{\mathsf{s}'}, \mathbb{D}|_{l'} \right\rangle_{l'}$

The first transition, $\lambda = \underline{k.C.l}$ consumed the first assignment of location and assigned the location of role $\mathtt{C}$ to $\underline{k.C.l}$ in the state of the starter t_{p}.

Let us suppose, without loss of generality, that $\mathtt{C} = \mathtt{A}$, then we have

$$P' = \left(\begin{array}{l} \bigodot_{\underline{I} \in \{\tilde{\mathtt{B}}\}} \underline{k.I.l} = l_{\mathtt{I}} \, ; \\ \bigodot_{\underline{I} \in \{\tilde{\mathtt{B}}\}} \Big(\nu \rangle \underline{k.I.A}; ?@\underline{k.I.l}(\underline{k}); sync(\underline{k}) \; \mathtt{from} \; \underline{k.I.A} \Big); \\ \bigodot_{\underline{I} \in \{\tilde{\mathtt{B}}\}} start@\underline{k.I.l}(\underline{k}) \; \mathtt{to} \; \underline{k.A.I}; \end{array} \right) ; \boxed{C_r}^{\Gamma} \bullet t_{\mathsf{p}} \lhd (\, \underline{k.A.l}, l \,)$$

and $\boxed{\langle\!\langle D \rangle\!\rangle^{\Gamma}, C}^{\Gamma} \xrightarrow{\underline{k.A.l}} S'$ where

$$S' = \Big\langle \boxed{C_c|_l}^{\Gamma}, P' \mid R, M \Big\rangle_l \mid \prod_{i=1}^{n} \langle Q_i, R_{l_i}, M_i \rangle_{l_i} \mid S_c$$

Since in its reduction D, C renames the new session with a fresh name, we first rename session k, in P and the service processes Q_i, to k', which is fresh. We take

$$P'' = P'[k'/k] = \left(\begin{array}{l} \bigodot_{\underline{I} \in \{\tilde{\mathtt{B}}\}} \underline{k'.I.l} = l_{\mathtt{I}} \, ; \\ \bigodot_{\underline{I} \in \{\tilde{\mathtt{B}}\}} \Big(\nu \rangle \underline{k'.I.A}; ?@\underline{k'.I.l}(\underline{k'}); ?@\underline{k'.I.l}(\underline{k'}); \Big); \\ \bigodot_{\underline{I} \in \{\tilde{\mathtt{B}}\}} sync(\underline{k'}) \; \mathtt{from} \; \underline{k'.I.A} \end{array} \right) ; \boxed{C_r}^{\Gamma} [\underline{k'}/\underline{k}] \bullet t''_{\mathsf{p}}$$

where, let $t'_{\mathsf{p}} = t_{\mathsf{p}} \lhd (\, \underline{k.A.l}, l \,)$, $t''_{\mathsf{p}} = t'_{\mathsf{p}} \lhd (\, \underline{k'}, \underline{k}(t'_{\mathsf{p}}) \,) \lhd (\, \underline{k}, \varnothing \,)$.

We take

$$S^*_0 = \Big\langle \boxed{C_c|_l}^{\Gamma}, P'' \mid R, M \Big\rangle_l \mid \prod_{i=1}^{n} \langle Q_i[\underline{k'}/\underline{k}], R_{l_i}, M_i \rangle_{l_i} \mid S_c$$

and by Lemma 14 we have $S^*_0 \sim S'$.

Now we can proceed with the rest of the transitions of the start procedure as defined at the beginning of the proof, so that $S^*_0 \to^+ S^*_1$. Finally we have

$$S^*_1 \sim S'' = \Big\langle \boxed{C_c|_l}^{\Gamma}, P''' \mid R, M' \Big\rangle_l \mid \prod_{i=1}^{n} \langle Q_i[\underline{k'}/\underline{k}], Q'_i \mid R_{l_i}, M'_i \rangle_{l_i} \mid S_c$$

where $P''' = \boxed{C_r}^{\Gamma} [\underline{k'}/\underline{k}] \bullet t'_p$ and $Q'_i = \boxed{C_{\mathsf{q}_i}}^{\Gamma} [\underline{k'}/\underline{k}] \bullet t_{k'}$

From the transitions presented above we know that there exists $t'_{k'}$ such that $t'_{\mathsf{p}} = t_{\mathsf{p}} \lhd (\, \underline{k'}, t'_{k'} \,)$, where $t'_{k'}$ is a session descriptor for session k' (i.e., it contains all the locations and correlations keys used by the processes in session k').

We proceed by proving that D, C can mimic $\boxed{\langle\!\langle D \rangle\!\rangle^{\Gamma}, C}^{\Gamma}$.

We can apply rules $\lfloor^{\mathsf{C}}|_{\mathsf{Par}}\rceil$ and $\lfloor^{\mathsf{C}}|_{\mathsf{Eq}}\rceil$ and lastly rule $\lfloor^{\mathsf{C}}|_{\mathsf{PStart}}\rceil$ such that

$$\frac{\begin{array}{c} i \in \{1, \ldots, n\} \qquad D \# k', \tilde{\mathsf{r}} \qquad \{\widetilde{l.\mathtt{B}}\} = \biguplus_i \{\widetilde{l_i.\mathtt{B}_i}\}_i \qquad \{\tilde{\mathsf{r}}\} = \bigcup_i \{\tilde{\mathsf{r}}_i\} \\ \delta = \mathsf{start}\; k' : \mathsf{p}[\mathtt{A}] \Leftrightarrow \widetilde{l_1.\mathsf{r}_1[\mathtt{B}_1]}, \ldots, \widetilde{l_n.\mathsf{r}_n[\mathtt{B}_n]} \qquad D, \delta \blacktriangleright D' \end{array}}{\begin{array}{l} D, \mathsf{req}\; k : \mathsf{p}[\mathtt{A}] \Leftrightarrow \widetilde{l.\mathtt{B}}; C \mid \prod_i \big(\mathsf{acc}\; k : \widetilde{l_i.\mathsf{q}_i[\mathtt{B}_i]}; C_i \big) \quad \to \\ \qquad D', \; C[k'/k] \mid \prod_i \big(\, C_i[k'/k][\tilde{\mathsf{r}}_i/\tilde{\mathsf{q}}_i] \, \big) \mid \prod_i \big(\mathsf{acc}\; k : \widetilde{l_i.\mathsf{q}_i[\mathtt{B}_i]}; C_i \big) \end{array}} \; \lfloor^{\mathsf{C}}|_{\mathsf{PStart}}\rceil$$

and

$$D,C \mid C_c \quad \rightarrow \quad D',C_r[k'/k] \mid \prod_i \left(C_{\mathsf{q}_i}[k'/k][\mathsf{r}_i/\mathsf{q}_i] \right) \mid \prod_{i=1}^{n} \mathsf{acc}\ k : l_i.\mathsf{q}_i[\mathsf{B}_i]; C_{\mathsf{q}_i} \mid C_c$$

thus $C' = C_r[k'/k] \mid \prod_{i=1}^{n} \left(C_{\mathsf{q}_i}[k'/k][\mathsf{r}_i/\mathsf{q}_i] \right) \mid \prod_{i=1}^{n} \mathsf{acc}\ k : l_i.\mathsf{q}_i[\mathsf{B}_i]; C_{\mathsf{q}_i} \mid C_c$.

From the hypothesis we know that $\Gamma \vdash D,C$ and therefore that $\Gamma = \Gamma_1, \tilde{l} \colon G\langle \mathtt{A}|\tilde{\mathtt{B}}|\tilde{\mathtt{B}}\rangle$. We can find $\Gamma' = \Gamma, \mathbf{init}(k', (\mathsf{p}[\mathtt{A}], \widetilde{\mathsf{q}[\mathtt{B}]}), G)$ and $\Gamma' \vdash D',C'$.

Finally, we need to prove that $S_1^* = \boxed{\langle\!\langle D' \rangle\!\rangle^{\Gamma'}, C'}^{\Gamma'}$.

From Definition 8 of the article we have

$$\boxed{\langle\!\langle D' \rangle\!\rangle^{\Gamma'}, C'}^{\Gamma'} = \left\langle \boxed{C_c|_l}^{\Gamma'}, P^* \mid R', M^* \right\rangle_l \mid \prod_{i=1}^{n} \left\langle Q_i'', Q_i^* \mid R_{l_i}', M_i^* \right\rangle_{l_i} \mid S_c'$$

Let $\mathbb{D}^* = \langle\!\langle D' \rangle\!\rangle^{\Gamma'}$ we use the abbreviations $t_{\mathsf{s}}^* = \mathbb{D}^*(\mathsf{s})$, for s process in $\mathbb{D}^*$, and $M^* = \mathbb{D}|_l$, and $M_i^* = \mathbb{D}|_{l_i}$, in $\boxed{\mathbb{D}^*, C'}^{\Gamma'}$ we have

- $P^* = \boxed{C_r[k'/k]}^{\Gamma'} \cdot t_{\mathsf{p}}^*$
- $R' = \prod_{\mathsf{p}' \,\in\, \mathbb{D}^*(l) \setminus \{\mathsf{p}\}} \boxed{C_c|_{\mathsf{p}'}}^{\Gamma'} \cdot t_{\mathsf{p}'}^*$
- $Q_i'' = \mathtt{accept}(k, \mathtt{B}_i, G\langle \mathtt{A}|\tilde{\mathtt{B}}|\tilde{\mathtt{B}}\rangle); \boxed{C_{\mathsf{q}_i}}^{\Gamma'}$
- $Q_i^* = \boxed{C_{\mathsf{q}_i}[k'/k][\mathsf{r}_i/\mathsf{q}_i]}^{\Gamma'} \cdot t_{q_i}^*$
- $R_{l_i}' = \prod_{\mathsf{s} \,\in\, \mathbb{D}^*(l_i)} \boxed{C_c|_{\mathsf{s}}}^{\Gamma'} \cdot t_{\mathsf{s}}^*$
- $S_c' = \prod_{l' \,\in\, \Gamma \setminus \{l, \tilde{l}\}} \left\langle \boxed{C_c|_{l'}}^{\Gamma'}, \prod_{\mathsf{s}' \,\in\, \mathbb{D}^*(l')} \boxed{C_c|_{\mathsf{s}'}}^{\Gamma'} \cdot t_{\mathsf{s}'}^*, \mathbb{D}^*|_{l'} \right\rangle_{l'}$

From Rule $\lfloor^{\mathsf{D}}|_{\mathsf{Start}}\rceil$ we know that

$$\underline{k'}(t_{\mathsf{p}}^*) = \underline{k'}(t_{\mathsf{q}_1}^*) = \ldots = \underline{k'}(t_{\mathsf{q}_n}^*) = t_{k'}$$

for some $t_{k'}$ session descriptor of session k'.

We prove the case by taking $t_{k'} = t_{k'}'$, $t_{k'}'$ obtained from the derivation of $\boxed{\langle\!\langle D \rangle\!\rangle^{\Gamma}, C}^{\Gamma}$ and $M^* = M'$ and $M_i^* = M_i'$, $i \in \{1, \ldots, n\}$.

Case $1 < |\{\tilde{\lambda}\}| < m - 1$
The case follows the same structure of the previous case. We rename k to k' on p and all the newly created service processes. Then we let the system complete all the transitions and prove that the reductum corresponds to the compilation of D', C'.

Case $|\{\tilde{\lambda}\}| = m$
Since $|\{\tilde{\lambda}\}| = m$ then $S = S'$ where S' has terminated all the transitions to start the session. Here we only have to rename k to k', as per Lemma 14, for all the involved processes, proving $S' = \boxed{\langle\!\langle D' \rangle\!\rangle^{\Gamma'}, C'}^{\Gamma'}$.

□

We now proceed to prove the *(Soundness)* of Theorem 3 of the article, restated here below to consider annotated transitions:

- *(Soundness)* $\boxed{D,C}^{\Gamma} \xrightarrow{\tilde{\lambda}} S$ implies *i)* $D,C \to^* D',C'$ and *ii)* $S \to^* \boxed{D',C'}^{\Gamma'}$ for some D',C', and Γ' such that *iii)* $\Gamma' \vdash D',C'$

In the following we use the shortcut

$$C_{start} = \mathtt{req}\; k : \mathtt{p}[\mathtt{A}] \Leftrightarrow l_1.[\mathtt{B}_1],\ldots,l_n.[\mathtt{B}_n];C_r \mid \prod_{i=1}^{n} \mathtt{acc}\; k : l_i.\mathtt{q}_i[\mathtt{B}_i];C_{\mathtt{q}_i}$$

Proof (Soundness) We proceed by induction on the cardinality of $\tilde{\lambda}$. Then we consider sub-cases on the shape of C and the shape of $\tilde{\lambda}$.

Case $|\{\tilde{\lambda}\}| = 0$

Trivial, $\boxed{D,C}^{\Gamma} = S = \boxed{D',C'}^{\Gamma'}$, $D,C = D',C'$, and $\Gamma \vdash D',C'$.

Case $|\{\tilde{\lambda}\}| = 1$
Since the cardinality of $\tilde{\lambda}$ is one, we can directly consider the single annotated transition $\lambda = \tilde{\lambda}$. In the sub-cases of this case we omit to consider impossible cases for $\lambda = \nu\rangle x$ and $\lambda = ?(x)$ since these transitions (corresponding respectively to rules $\lfloor^{\mathsf{DCC}}|_{\mathsf{Newque}}\rceil$, and $\lfloor^{\mathsf{DCC}}|_{\mathsf{InStart}}\rceil$ or $\lfloor^{\mathsf{DCC}}|_{\mathsf{Start}}\rceil$) can happen only within of a start session sequence (i.e., not at the first position).

In the following we use the abbreviation *follows* (#) to indicate that the case unfolds following the proof of **Case** # for the same subcase for λ, with the thesis following by applying the induction hypothesis.

Case $C = k\colon\mathtt{A} \longrightarrow \mathtt{q}[\mathtt{B}].\{o_i(x_i);C_i\}_{i\in I};C_{\mathtt{q}} \mid C_c$

Case $\lambda = x$ *follows* ($C = C_{start} \mid C_c$).

Case $\lambda = o\ \mathtt{to}\ x$ *follows* ($C = k\colon\mathtt{p}[\mathtt{A}].e \longrightarrow \mathtt{B}.o;C_{\mathtt{p}} \mid C_c$).

Case $\lambda = \tau$ *follows* ($C = \mathtt{if}\ \mathtt{p}.e\ \{C_1\}\ \mathtt{else}\ \{C_2\} \mid C_c$).

Case $\lambda = o\ \mathtt{from}\ x$
Since receptions in compiled DCC systems can only happen on correlating queues within sessions, without loss of generality we can assume that $\lambda = o\ \mathtt{from}\ \underline{k.A.B}$ where $o_j \notin \{start, sync\}$, indeed these operation names are reserved for session initiation and cannot appear as first (in this case, only) reduction of a compiled system.
Let $\mathtt{q}@l \in \Gamma$, $\mathbb{D} = \langle\!\langle D \rangle\!\rangle^{\Gamma}$, $M = \mathbb{D}|_l$, and $t_{\mathtt{q}} = \mathbb{D}(\mathtt{q})$. Then, from Definition 8 of the article and Theorem 1 of the article, we have

$$\boxed{D,C}^{\Gamma} \equiv_{\mathsf{D}} \left\langle \boxed{C_c|_l}^{\Gamma}, Q \mid R, M \right\rangle_l \mid S_c$$

where

- $Q = \sum_{i\in I} [o_i(x_i)\ \mathtt{from}\ \underline{k.A.B}]\ \{\boxed{C_i}^{\Gamma}\} \cdot t_{\mathtt{q}}$
- $R = \prod_{\mathtt{r}\, \in\, \mathbb{D}(l)\setminus\{\mathtt{q}\}} \boxed{C_c|_{\mathtt{r}}}^{\Gamma} \cdot t_{\mathtt{r}}$
- $S_c = \prod_{l'\in\Gamma\setminus\{l\}} \left\langle \boxed{C_c|_{l'}}^{\Gamma}, \prod_{\mathtt{s}\, \in\, D(l')} \boxed{C_c|_{\mathtt{s}}}^{\Gamma} \cdot t_{\mathtt{s}} \right\rangle_{l'}$

Let $t_c = \mathbf{eval}(\underline{k.A.B}, t_{\mathtt{q}})$, $t_m = \mathbf{eval}(e, t_{\mathtt{q}})$, and $M(t_c) = (o_j, t_m) :: \tilde{m}$, then we can apply rules $\lfloor^{\mathsf{DCC}}|_{\mathsf{Eq}}\rceil$, $\lfloor^{\mathsf{DCC}}|_{\mathsf{SPar}}\rceil$, and $\lfloor^{\mathsf{DCC}}|_{\mathsf{Recv}}\rceil$ such that

$$\boxed{D,C}^{\Gamma} \xrightarrow{o_j\ \mathtt{from}\ \underline{k.A.B}} S$$

where

$$S = S' \mid S_c$$

and $S' = \left\langle \boxed{C_c|_l}^{\Gamma}, \boxed{C_j}^{\Gamma} \cdot t_{\mathsf{q}} \triangleleft (x_j, t_m) \mid R, M[t_c \mapsto \tilde{m}] \right\rangle_l$.
D, C can mimic $\boxed{D, C}^{\Gamma}$ with rules $\lfloor^{\mathsf{C}}|_{\mathsf{Eq}}\rceil$, $\lfloor^{\mathsf{C}}|_{\mathsf{Par}}\rceil$, and $\lfloor^{\mathsf{C}}|_{\mathsf{Recv}}\rceil$ for which

$$D, C \quad \rightarrow \quad D', C_{\mathsf{p}} \mid C_c$$

Let $D(k[\mathtt{A}\rangle\mathtt{B}]) = (o_j, v_m) :: \tilde{m}'$. Then, we have

$$D' = D[\mathsf{q} \mapsto t_{\mathsf{q}} \triangleleft (x_j, v_m)][k[\mathtt{A}\rangle\mathtt{B}] \mapsto \tilde{m}']$$

Since from the premises $\Gamma \vdash D, C$ then
$\Gamma = \Gamma_1, k[\mathtt{A}] : \&\mathtt{A}.\{o_i(U_i); T_i\}_{i \in I}, k[\mathtt{A}\rangle\mathtt{B}] : \&\mathtt{A}.o_j(U_j); T'$ and we can find
$\Gamma' = \Gamma_1, k[\mathtt{A}] : T_j, k[\mathtt{A}\rangle\mathtt{B}] : T'$ such that $\Gamma' \vdash D', C'$.

At the level of choreographies, since the changes in D' and Γ' and the related $\mathbb{D}' = \boxed{D'}^{\Gamma'}$ affect only the queue related to $\mathbb{D}'|_l$ and the state of q, for all other terms $\boxed{\cdot}^{\Gamma} = \boxed{\cdot}^{\Gamma'}$ and $\mathbb{D}'|_{l'} = \mathbb{D}|_{l'}$.
Hence we can write $\boxed{D', C'}^{\Gamma'} = S'' \mid S_c$ where $S'' = S'$ by Theorem 1 of the article.

Case $C = k : \mathsf{p}[\mathtt{A}].e \longrightarrow \mathtt{B}.o; C_{\mathsf{p}} \mid C_c$

Case $\lambda = x$ *follows* ($C = C_{start} \mid C_c$).

Case $\lambda = o \; \mathtt{from} \; x$ *follows* ($C = k : \mathtt{A} \longrightarrow \mathsf{q}[\mathtt{B}].\{o_i(x_i); C_i\}_{i \in I}; C_{\mathsf{q}} \mid C_c$).

Case $\lambda = \tau$ *follows* ($C = \mathtt{if} \; \mathsf{p}.e \; \{C_1\} \; \mathtt{else} \; \{C_2\} \mid C_c$).

Case $\lambda = o \; \mathtt{to} \; x$

As for Case $C = k : \mathtt{A} \longrightarrow \mathsf{q}[\mathtt{B}].\{o_i(x_i); C_i\}_{i \in I}; C_{\mathsf{q}} \mid C_c$, we know that all send actions in DCC systems compiled from FC programs happen on a session-related queues, hence we can assume $\lambda = o@\underline{k.A.B}$. Also, we know that $o \notin \{start, sync\}$ for the reasons explained in Case $C = k : \mathtt{A} \longrightarrow \mathsf{q}[\mathtt{B}].\{o_i(x_i); C_i\}_{i \in I}; C_{\mathsf{q}} \mid C_c$.
From Theorem 1 of the article, let $\mathbb{D} = \langle\!\langle D \rangle\!\rangle^{\Gamma}$, $t_{\mathsf{p}} = \mathbb{D}(\mathsf{p})$, and $M = \mathbb{D}|_l$. Now we consider two cases for which, let $\mathsf{p}@l \in \Gamma$, whether the location of the receiving process (stored under path $\underline{k.B.l}$ in the state of p) equals l, we either reduce the compiled DCC system by means of rule $\lfloor^{\mathsf{DCC}}|_{\mathsf{InSend}}\rceil$ or rule $\lfloor^{\mathsf{DCC}}|_{\mathsf{Send}}\rceil$. For brevity we just consider the case for $\lfloor^{\mathsf{DCC}}|_{\mathsf{InSend}}\rceil$ as the other case follows similarly.
Since $\lfloor^{\mathsf{DCC}}|_{\mathsf{InSend}}\rceil$ applies, we can infer that

$$\boxed{D, C}^{\Gamma} \equiv_{\mathsf{D}} \left\langle \boxed{C_c|_l}^{\Gamma}, P \mid Q \mid R, M \right\rangle_l \mid S_c$$

where

- $P = o@\underline{k.B.l} \; \mathtt{to} \; \underline{k.A.B}; \boxed{C_{\mathsf{p}}}^{\Gamma} \cdot t_{\mathsf{p}}$
- $Q = \boxed{C_c|_{\mathsf{q}}}^{\Gamma} \cdot t_{\mathsf{q}}$
- $R = \prod_{\mathsf{r} \in D(l) \setminus \{\mathsf{p}, \mathsf{q}\}} \boxed{C_c|_{\mathsf{r}}}^{\Gamma} \cdot t_{\mathsf{r}}$
- $S_c = \prod_{l' \in \Gamma \setminus \{l\}} \left\langle \boxed{C_c|_{l'}}^{\Gamma}, \prod_{\mathsf{s} \in \mathbb{D}(l')} \boxed{C_c|_{\mathsf{s}}}^{\Gamma} \cdot t_{\mathsf{s}}, \mathbb{D}|_{l'} \right\rangle_{l'}$

Let $t_c = \mathbf{eval}(\underline{k.A.B}, t_\mathsf{p})$, $t_m = \mathbf{eval}(e, t_\mathsf{p})$, and $M(t_c) = \tilde{m}$, then

$$\boxed{D,C}^{\Gamma} \xrightarrow{o@\underline{k.A.B}} S$$

where

$$S = S' \mid S_c$$

and $S' = \left\langle \boxed{C_c|_l}^{\Gamma}, \boxed{C_\mathsf{p}}^{\Gamma} \cdot t_\mathsf{p} \mid \boxed{C_c|_\mathsf{q}}^{\Gamma} \cdot t_\mathsf{q} \mid R, M[t_c \mapsto \tilde{m} :: (o, t_m)] \right\rangle_l$
D,C can mimic $\boxed{D,C}^{\Gamma}$ with rules $\lfloor^{C}|_{\mathsf{Eq}}\rceil$, $\lfloor^{C}|_{\mathsf{Par}}\rceil$, and $\lfloor^{C}|_{\mathsf{Send}}\rceil$ for which

$$D,C \quad \to \quad D', C_\mathsf{p} \mid C_c$$

where, let $v_m = \mathbf{eval}(e, D(\mathsf{p}))$ and $\tilde{m}' = D(k[\mathtt{A}\rangle\mathtt{B}])$, $D' = D[k[\mathtt{A}\rangle\mathtt{B}] \mapsto \tilde{m}' :: (o, v_m)]$. Since from the premises $\Gamma \vdash D,C$ then $\Gamma = \Gamma_1, k[\mathtt{A}]: \oplus\, \mathtt{B}.o(U); T, k[\mathtt{A}\rangle\mathtt{B}]: T'$ and we can find $\Gamma' = \Gamma_1, k[\mathtt{A}]: T, k[\mathtt{A}\rangle\mathtt{B}]: T'; \&\mathtt{A}.o(U)$ such that $\Gamma' \vdash D', C'$.

At the level of choreographies, since the changes in D' and Γ' and the related $\mathbb{D}' = \boxed{D'}^{\Gamma'}$ affect only the queue related to $\mathbb{D}'|_l$, for all other terms $\boxed{\cdot}^{\Gamma} = \boxed{\cdot}^{\Gamma'}$ and $\mathbb{D}'|_{l'} = \mathbb{D}|_{l'}$. Hence we can write $\boxed{D',C'}^{\Gamma'} = S'' \mid S_c$ where $S'' = S'$ by Theorem 1 of the article.

Case $C = C_{start} \mid C_c$

Case $\lambda = o\ \mathtt{from}\ x$ *follows* ($C = k\colon \mathtt{A} \longrightarrow \mathsf{q}[\mathtt{B}].\{o_i(x_i); C_i\}_{i \in I}; C_\mathsf{q} \mid C_c$).
Case $\lambda = o\ \mathtt{to}\ x$ *follows* ($C = k\colon \mathsf{p}[\mathtt{A}].e \longrightarrow \mathtt{B}.o; C_\mathsf{p} \mid C_c$)
Case $\lambda = \tau$ *follows* ($C = \mathtt{if}\ \mathsf{p}.e\ \{C_1\}\ \mathtt{else}\ \{C_2\} \mid C_c$).

Case $\lambda = x$
From Definition 8 of the article we know that assignments in DCC systems that are compiled from FC programs appear only within the starting of a session. In this case, since $\tilde{\lambda}$ contains only one action which corresponds to the first reduction of the compiled DCC system, it must be the first assignment for the creation of the session descriptor for some session k' in C.
Let $\mathtt{C} \in \{\mathtt{A}, \tilde{\mathtt{B}}\}$, we have two subcases whether $\tilde{\lambda} = \lambda = \underline{k.C.l}$ or $\tilde{\lambda} = \lambda = \underline{k''.C.l}$, i.e., whether we are starting session k or we are starting another session k''.

Case $\lambda = \underline{k.C.l}$
In this case $\boxed{D,C}^{\Gamma}$ is starting a new session on k. The case is proved applying Lemma 16.

Case $\lambda \neq \underline{k'.C.l}$
In this case we are starting a session on $k'' \neq k$. The case unfolds following the proof of case $C = C_{start} | C_c$ where C_c contains the endpoint choreographies for the starter process and the service processes for session k''. The thesis follows by applying the induction hypothesis.

Case $C = \mathtt{if}\ \mathsf{p}.e\ \{C_1\}\ \mathtt{else}\ \{C_2\} \mid C_c$

Case $\lambda = x$ *follows* ($C = C_{start} \mid C_c$).
Case $\lambda = o\ \mathtt{from}\ x$ *follows* ($C = k\colon \mathtt{A} \longrightarrow \mathsf{q}[\mathtt{B}].\{o_i(x_i); C_i\}_{i \in I}; C_\mathsf{q} \mid C_c$).
Case $\lambda = o\ \mathtt{to}\ x$ *follows* ($C = k\colon \mathsf{p}[\mathtt{A}].e \longrightarrow \mathtt{B}.o; C_\mathsf{p} \mid C_c$).

Case $\lambda = \tau$

In this case, the label does not allow us to establish a correspondence between the considered shape of C and the actual reduction annotated by the label.

However, since τ labels only correspond to the reduction of conditionals, without loss of generality, we can consider here only the case where the reduction acts on the considered term. The other case follows the unfolding of this case for the term reduced by the action and the induction hypothesis.

Let $\mathsf{p}@l \in \Gamma$. From Definition 8 of the article we have

$$\boxed{D,C}^{\Gamma} \equiv_{\mathsf{D}} \left\langle \boxed{C_c|_l}^{\Gamma}, P \mid R, M \right\rangle_l \mid S_c$$

where, let $\mathbb{D} = \langle\!\langle D \rangle\!\rangle^{\Gamma}$, $t_{\mathsf{p}} = \mathbb{D}(\mathsf{p})$, and $M = \mathbb{D}|_l$

- $P = \mathtt{if}\ \mathsf{p}.e\ \{\boxed{C_1}^{\Gamma}\}\ \mathtt{else}\ \{\boxed{C_2}^{\Gamma}\} \cdot t_{\mathsf{p}}$
- $R = \prod_{\mathsf{r} \in \mathbb{D}(l) \setminus \{\mathsf{p}\}} \boxed{C_c|_{\mathsf{r}}}^{\Gamma} \cdot t_{\mathsf{r}}$
- $S_c = \prod_{l' \in \Gamma \setminus \{l\}} \left\langle \boxed{C_c|_{l'}}^{\Gamma}, \prod_{\mathsf{s} \in \mathbb{D}(l')} \boxed{C_c|_{\mathsf{s}}}^{\Gamma} \cdot t_{\mathsf{s}}, \mathbb{D}|_{l'} \right\rangle_{l'}$

The case unfolds into two cases, on whether $\mathbf{eval}(e, \mathbb{D}(\mathsf{p})) = \mathtt{true}$. Here we proceed with the positive case. The other case follows the same structure.

We proceed considering that $\mathbf{eval}(e, \mathbb{D}(\mathsf{p})) = \mathtt{true}$. $\boxed{D,C}^{\Gamma}$ reduces with rules $\lfloor^{\mathsf{DCC}}|_{\mathsf{Eq}}\rceil$, $\lfloor^{\mathsf{DCC}}|_{\mathsf{SPar}}\rceil$, $\lfloor^{\mathsf{DCC}}|_{\mathsf{Par}}\rceil$, and $\lfloor^{\mathsf{DCC}}|_{\mathsf{Cond}}\rceil$ such that

$$\boxed{D,C}^{\Gamma} \quad \to \quad \left\langle \boxed{C_c|_l}^{\Gamma}, \boxed{C_c|_{\mathsf{p}}}^{\Gamma} \cdot t_{\mathsf{p}} \mid R, M \right\rangle_l \mid S_c$$

where $S = \left\langle \boxed{C_c|_l}^{\Gamma}, \boxed{C_c|_l}^{\Gamma} \cdot t_{\mathsf{p}} \mid R, M \right\rangle_l \mid S_c$. D,C can mimic $\boxed{D,C}^{\Gamma}$ with rules $\lfloor^{\mathsf{C}}|_{\mathsf{Eq}}\rceil$, $\lfloor^{\mathsf{C}}|_{\mathsf{Par}}\rceil$, and $\lfloor^{\mathsf{C}}|_{\mathsf{Cond}}\rceil$ such that

$$D,C \quad \to \quad D,C_1 \mid C_c$$

We choose $\Gamma' = \Gamma$ for which it holds that $\Gamma \vdash D, C_1 \mid C_c$.

Finally, $\boxed{D,C_1 \mid C_c}^{\Gamma} = S$ by Definition 8 of the article.

Finally, **Case**s $C = C_1 \mid C_2$, $C = \mathtt{def}\ X = C'\ \mathtt{in}\ C_{\mathsf{p}} \mid C_c$, $C = X \mid C_c$ and $C = \mathbf{0} \mid C_c$ unfold applying the induction hypothesis on the respective sub-cases **Case** $\lambda = x$ *follows* ($C = C_{start} \mid C_c$), **Case** $\lambda = o\ \mathtt{from}\ x$ *follows* ($C = k\colon \mathtt{A} \multimap \mathsf{q}[\mathtt{B}].\{o_i(x_i); C_i\}_{i \in I}; C_{\mathsf{q}} \mid C_c$), **Case** $\lambda = o\ \mathtt{to}\ x$ *follows* ($C = k\colon \mathsf{p}[\mathtt{A}].e \multimap \mathtt{B}.o; C_{\mathsf{p}} \mid C_c$), **Case** $\lambda = \tau$ *follows* ($C = \mathtt{if}\ \mathsf{p}.e\ \{C_1\}\ \mathtt{else}\ \{C_2\} \mid C_c$).

Case $|\{\tilde{\lambda}\}| > 1$

The case unfolds considering λ as the first action in $\tilde{\lambda} = \lambda, \tilde{\lambda}'$. For any shape of C and label $\lambda \neq x$ we can *i*) apply the same steps followed in the related case for the same C with $|\{\tilde{\lambda}\}| = 1$, $\tilde{\lambda} = \lambda$ and *ii*) inductively unfold the case on the remaining part $\tilde{\lambda}'$.

For $\lambda = x$ and C of shape $C_{start} \mid C_c$ (the case for other shapes of C can be re-conducted to this case), let $x = \underline{k.A.l}$ (other cases for $x = \underline{k'.B.l}$ are similar) and the thesis follows by applying Lemma 15, Lemma 16 and the induction hypothesis on the remaining transitions in $\tilde{\lambda} \setminus \tilde{\lambda}|_k$.

□

REFERENCES

Busi, N., Gorrieri, R., Guidi, C., Lucchi, R., and Zavattaro, G. (2006). Choreography and orchestration conformance for system design. In *Coordination Models and Languages: 8th International Conference, COORDINATION 2006, Bologna, Italy, June 14-16, 2006. Proceedings 8*, pages 63–81. Springer.

Carbone, M., Honda, K., and Yoshida, N. (2012). Structured communication-centered programming for web services. *ACM Transactions on Programming Languages and Systems (TOPLAS)*, 34(2):1–78.

Carbone, M. and Montesi, F. (2013). Deadlock-freedom-by-design: multiparty asynchronous global programming. In *POPL*, pages 263–274.

Gay, S. and Hole, M. (2005). Subtyping for session types in the pi calculus. *Acta Informatica*, 42(2-3):191–225.

Honda, K., Yoshida, N., and Carbone, M. (2016). Multiparty asynchronous session types. *Journal of the ACM (JACM)*, 63(1):1–67.

Hüttel, H., Lanese, I., Vasconcelos, V. T., Caires, L., Carbone, M., Deniélou, P.-M., Mostrous, D., Padovani, L., Ravara, A., Tuosto, E., Vieira, H. T., and Zavattaro, G. (2016). Foundations of session types and behavioural contracts. *ACM Comput. Surv.*, 49(1).

Montesi, F. and Yoshida, N. (2013). Compositional choreographies. In *CONCUR*, pages 425–439.

Pierce, B. C. (2002). *Types and Programming Languages*. MIT Press, MA, USA.

Qiu, Z., Zhao, X., Cai, C., and Yang, H. (2007). Towards the theoretical foundation of choreography. In *WWW*, pages 973–982. IEEE Computer Society Press.
